# Helicity Control in the Aggregation of Achiral Squaraine Dyes in Solution and Thin Films

**DOI:** 10.1002/chem.202002695

**Published:** 2020-11-09

**Authors:** Andreas T. Rösch, Qirong Zhu, Jorn Robben, Francesco Tassinari, Stefan C. J. Meskers, Ron Naaman, Anja R. A. Palmans, E. W. Meijer

**Affiliations:** ^1^ Laboratory of Macromolecular and Organic Chemistry, and Institute for Complex Molecular Systems Department of Chemical Engineering and Chemistry Eindhoven University of Technology P.O. Box 513, 5600 MB Eindhoven The Netherlands; ^2^ Department of Chemical and Biological Physics Weizmann Institute of Science Rehovot 76100 Israel; ^3^ Department of Applied Physics Eindhoven University of Technology P.O. Box 513, 5600 MB Eindhoven The Netherlands

**Keywords:** circular dichroism, helical structures, self-assembly, squaraine dyes, supramolecular chemistry

## Abstract

Squaraine dyes are well known for their strong absorption in the visible regime. Reports on chiral squaraine dyes are, however, scarce. To address this gap, we here report two novel chiral squaraine dyes and their achiral counterparts. The presented dyes are aggregated in solution and in thin films. A detailed chiroptical study shows that thin films formed by co‐assembling the chiral dye with its achiral counterpart exhibit exceptional photophysical properties. The circular dichroism (CD) of the co‐assembled structures reaches a maximum when just 25 % of the chiral dye are present in the mixture. The solid structures with the highest relative CD effect are achieved when the chiral dye is used solely as a director, rather than the structural component. The chiroptical data are further supported by selected spin‐filtering measurements using mc‐AFM. These findings provide a promising platform for investigating the relationship between the dissymmetry of a supramolecular structure and emerging material properties rather than a comparison between a chiral molecular structure and an achiral counterpart.

## Graphical abstract

**Helicity control**: An unexpected outcome of the co‐assembly of achiral squaraine dye **a‐SQ‐1** with its chiral counterpart ***S*** **‐SQ‐1** is reported. A maximum in optical activity is observed in the supramolecular aggregates of a 3:1 mixture with **a‐SQ‐1** as the major component. Intriguingly, the highest spin‐selective electron transport is observed for this mixing ratio as well, illustrating the direct relationship between optical activity and spin‐selective electron transport.

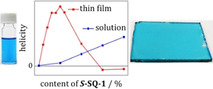

## Introduction

The high absorption of light in the visible and near‐infrared regime displayed by squaraine dyes has intrigued many research groups in the last 60 years.[^1^, ^2^, ^3^] The unique photophysical properties arise from the intramolecular charge‐transfer between the electron‐poor quadratic core and the fused electron‐rich arene substituents. Previous studies have shown that squaraine dyes can be used in various applications such as (bio‐)labelling,[^4^, ^5^, ^6^] photodynamic therapy,^[7]^ dye‐sensitised solar cells[^8^, ^9^, ^10^, ^11^] and organic bulk heterojunction solar cells.^[12]^ Despite numerous studies investigating the photophysical properties of squaraine dyes, the number of reports dealing with chiral analogues is limited.[^13^, ^14^, ^15^, ^16^, ^17^, ^18^, ^19^] Nonetheless, their potential in chiroptical applications such as detection[^20^, ^21^] and emission^[22]^ of circularly polarised light or organic spintronics^[23]^ is high. The paucity of these reports is likely due to the fact that these fields of application are of recent date.

The emerging research on organic spintronics focusses on improved fundamental understanding of the role of chirality and the electron spin or electromagnetic fields in both chemical[^24^, ^25^] and biological systems.[^26^, ^27^, ^28^] It has been shown that electrons with initially random electron spin‐orientation can be spin‐polarised by transmission through a highly ordered chiral layer of double stranded DNA.^[29]^ Manifestation of spin‐polarisation in organic materials is described by the so‐called chiral‐induced spin selectivity (CISS) effect.^[23]^ Even though the measured spin polarisation achieved in organic materials is never absolute in the systems described until now,^[30]^ various applications such as water‐splitting,[^24^, ^31^, ^32^, ^33^] enantio‐separation[^34^, ^35^] and enantioselective reactions^[36]^ have been realised.

**Scheme 1:**
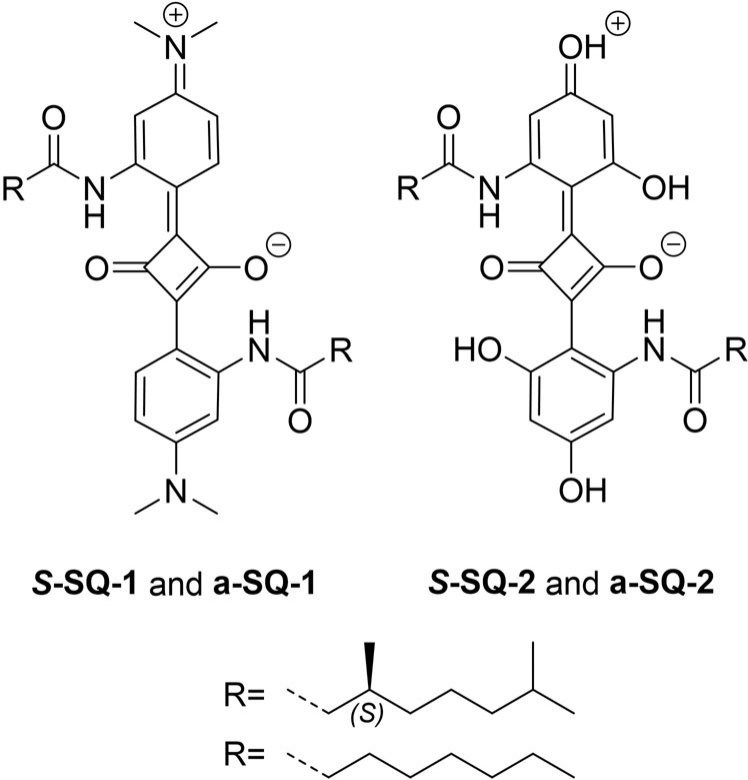
Molecular structures of amide functionalised squaraine dyes.

Inspired by reports on the giant intrinsic circular dichroism of prolinol‐derived squaraines in thin film,^[19]^ we designed two novel types of symmetrical dyes as depicted in Scheme 1, with the aim to explore the unique photophysical properties of squaraine dyes with application in organic electronics and spintronics. Previous studies on squaraine dyes have focused often on nitrogen‐based heterocycles[^4^, ^16^, ^37^, ^38^, ^39^] or *N*,*N*‐dialkylaminoaryl substituted derivatives[^6^, ^12^, ^40^] since the formation of the squaraine unit succeeds only when the nucleophilicity of the reagent used for the condensation reaction with squaric acid is sufficiently high.^[41]^ Squaraine dyes that comprise *para*‐hydroxy substitution have received less attention.[^42^, ^43^, ^44^] Replacement of the tertiary amine group in the squaraine precursor by a hydroxyl group renders the resulting squaraine dye an acid, which readily reacts with traces of water present in organic solvent.^[41]^

Both presented dyes combine squaraine motifs with amide functionalised side chains. The combination of amide functionalised side chains and planar motifs such as the benzene‐1,3,5‐tricarboxamide,^[45]^ porphyrin^[46]^ or naphthalene diimide (NDI)^[47]^ is well‐described in the literature to induce the formation of supramolecular aggregates. The successful attachment of amide substituents to the squaraine motif in order to induce supramolecular aggregation has so far been reported only once for an achiral squaraine dye derivative.^[48]^ By introducing chiral, non‐racemic substituents, we anticipate the formation of structures with a preferred helicity upon aggregation in selected solvents or in the bulk.[^49^, ^50^]

Since the dyes contain either *N*,*N*‐dialkylaminoaryl or hydroxy aryl substituents, they will be sensitive to differences in pH and solvent polarity. Stimuli‐responsiveness is considered valuable for the design of future smart materials.^[51]^ The combination of the intriguing photophysical properties of squaraine dyes in the visible regime with the successful preparation of aggregates, which exhibit ordering with preferred helicity, is regarded as a key element towards novel materials for application in organic spintronics.

## Results and Discussion

### Synthesis of chiral (*S*‐) and achiral (a‐) SQ‐1 and SQ‐2

**Scheme 2:**
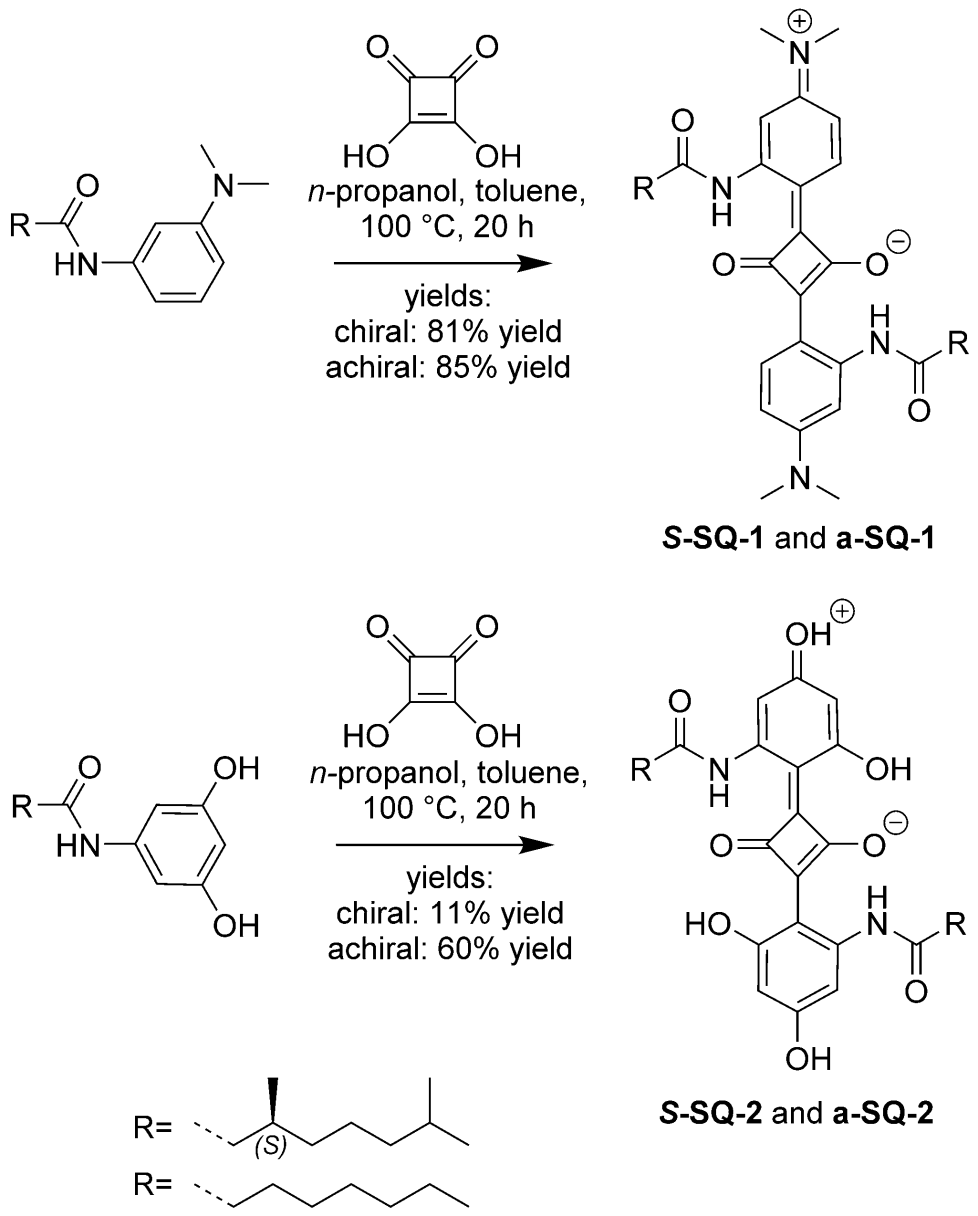
Synthesis route starting from amide functionalised arenes that are reacted with squaric acid to yield ***S*** **‐SQ‐1** and **a‐SQ‐1** as green, and ***S*** **‐SQ‐2** and **a‐SQ‐2** as blue solids.

*N*,*N*‐Dialkylamino‐aryl and hydroxyl‐aryl substituted squaraine dyes ***S*** **‐SQ‐1** and **a‐SQ‐1** as well as ***S*** **‐SQ‐2** and **a‐SQ‐2**, were prepared according to Scheme 2. After obtaining an amide substituted precursor (see Scheme S1 in the Supporting Information for detailed synthetic procedures), condensation with squaric acid was performed using a modification of the original procedure by Sprenger and Ziegenbein.^[39]^ As described by a more recent study, *n*‐butanol was replaced by the lower‐boiling *n*‐propanol to avoid the formation of the undesired 1,2‐condensation product.^[17]^ The successful dye formation was indicated by the development of an intense green (***S*** **‐SQ‐1** and **a‐SQ‐1**) or blue (***S*** **‐SQ‐2** and **a‐SQ‐2**) colour. ***S*** **‐SQ‐1** and **a‐SQ‐1** were obtained in excellent (89–94 %) and ***S*** **‐SQ‐2** and **a‐SQ‐2** in reasonable (14–60 %) yields. The compounds were fully characterised with NMR, MALDI‐TOF‐MS, elemental analysis and IR spectroscopy (spectra are shown in the Supporting Information). These results show that the final compounds were obtained in high purity, with a perfect regioselectivity.

### Photophysical properties in solution

**Figure 1:**
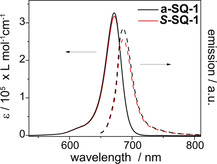
Molar extinction coefficients *ϵ* (solid lines) and fluorescence intensity (dashed lines, λ_ex_=640 nm) of ***S*** **‐SQ‐1** and **a‐SQ‐1** recorded in tetrahydrofuran. The concentrations are 2.6×10^−6^ mol L^−1^.

We first focus on *N*,*N*‐dialkylaminoaryl based squaraine dyes ***S*** **‐SQ‐1** and **a‐SQ‐1**. Figure 1 shows the molar extinction coefficients (*ϵ*) of ***S*** **‐SQ‐1** and **a‐SQ‐1** as a function of the wavelength in tetrahydrofuran (THF) solution. Both dyes show a sharp maximum of *ϵ* around 670 nm and a weak shoulder at 620 nm. The recorded spectra were found to be consistent with reports on squaraine dyes in the molecularly dissolved state.[^2^, ^11^, ^16^] ***S*** **‐SQ‐1** and **a‐SQ‐1** are highly fluorescent in the molecularly dissolved state. The emission spectra represent mirror images of the absorption spectra as clearly noticed in Figure 1. The corresponding Stokes shifts are 15 nm. The high *ϵ* of 3.2×10^5^ and 3.3×10^5^ L mol^−1^ cm^−1^ for ***S*** **‐SQ‐1** and **a‐SQ‐1**, respectively, and the narrow absorption bandwidths are the result of the weak vibronic coupling for the electronic *S*_0_→*S*_1_ transition along the long molecular axis of the squaraine backbone.^[52]^ Weak vibronic coupling is commonly caused by a low configurational displacement between initial and final state of an electronic transition and accompanied by weak manifestation of vibronic bands in the absorption spectrum.

The small shoulder in the absorption spectrum of ***S*** **‐SQ‐1** and **a‐SQ‐1** is assigned to the weak vibronic progression of the absorption band. A quantitative theory on how strong vibrational progressions are observed in the absorption spectrum was developed by Huang and Rhys.^[53]^ After fitting the recorded spectral data as shown in Figure S1, the Huang and Rhys parameter S was estimated to be 0.16, which corroborates the weak vibronic coupling.^[54]^

In contrast to ***S*** **‐SQ‐1** and **a‐SQ‐1**, ***S*** **‐SQ‐2** and **a‐SQ‐2** exhibited shorter wavelengths of maximum absorbance of *λ*=606 nm (for spectra see Figures S2–S5) and a decreased molar absorptivity (*ϵ*(***S*** **‐SQ‐2**)_606_  n_m_=1.5×10^5^ L mol^−1^ cm^−1^ and *ϵ*(**a‐SQ‐2**)_606_  n_m_=1.6 ×10^5^ L mol^−1^ cm^−1^). The shifts in values suggest a less pronounced intramolecular charge transfer for ***S*** **‐SQ‐2** and **a‐SQ‐2** compared to ***S*** **‐SQ‐1** and **a‐SQ‐1**.

Subsequently, absorption and emission spectra of all dyes were measured in a range of solvents (acetonitrile, chloroform, heptane, methanol and water) that differ in polarity. The recorded spectra are shown in Figures S2–S5. For all dyes, the absorption band of the molecularly dissolved species depends on the solvent polarity and responds to the addition of an acid or base. A detailed characterisation of the manipulation of the photophysical properties upon the addition of acids or bases is given in the Supporting Information (See Figures S6–S8). During the study, we found that ***S*** **‐SQ‐2** and **a‐SQ‐2** have a limited chemical stability when molecularly dissolved in dimethyl sulfoxide. An NMR study on the stability is presented in the Supporting Information (Figure S9). As a result, we decided to focus our further investigations on ***S*** **‐SQ‐1** and **a‐SQ‐1**.

### Formation of CD active aggregates of *S*‐SQ‐1 and a‐SQ‐1 in solution

**Figure 2:**
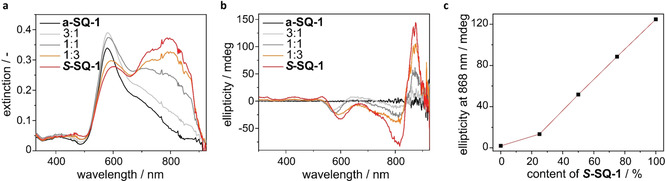
Photophysical properties of aggregates of ***S*** **‐SQ‐1** and **a‐SQ‐1** and mixtures thereof, recorded at a total concentration of 1.3×10^−5^ mol L^−1^ in H_2_O containing 1 vol% tetrahydrofuran. (a) UV/Vis spectra; (b) CD spectra; (c) Ellipticity measured at 868 nm.

The aggregation of ***S*** **‐SQ‐1** and **a‐SQ‐1** and mixtures thereof is induced by adding a concentrated solution of dye in THF to water, which is a poor solvent. Upon the addition, an immediate colour change from green to blue is observed for both ***S*** **‐SQ‐1** and **a‐SQ‐1**, which suggests that the aqueous solution containing 1 vol % THF induces the formation of aggregates. The absorption spectra of the aggregated dyes are depicted in Figure 2 a. In order to ensure complete dissolution and equilibration during the mixing experiments, the samples were heated and stirred at 90 °C, followed by slow cooling to room temperature before each measurement. This procedure was followed throughout this study. The absorption spectrum of aggregated **a‐SQ‐1** (black line in Figure 2 a) shows one absorption maximum around 590 nm with a broad shoulder towards longer wavelengths. The wavelength of maximum absorbance is blue‐shifted (H‐band) with respect to the absorption band of the molecularly dissolved species (670 nm). Upon addition of ***S*** **‐SQ‐1** to the feed and equilibration following the protocol, the rise of an absorption band with maximum around 800 nm is observed. The band is most pronounced for pure ***S*** **‐SQ‐1** (red line in Figure 2 a) and red‐shifted (J‐band) with respect to the absorption band of the molecularly dissolved species. The gradual increase of the J‐band indicates changes in aggregate structure when tuning the dye feed from **a‐SQ‐1** to ***S*** **‐SQ‐1**. In the spectra of all aqueous solutions, the absorption band of the molecularly dissolved species is noticed as a small shoulder only, which indicates the pronounced conversion from the molecularly dissolved state to aggregates.

Circular dichroism (CD) spectra were measured for solutions of ***S*** **‐SQ‐1**, **a‐SQ‐1** and mixtures thereof. The results are depicted in Figure 2 b and show that the aggregates formed by ***S*** **‐SQ‐1** exhibit circular dichroism whereas aggregates of **a‐SQ‐1** do not, as expected.

Since no CD effect was observed for molecularly dissolved neutral or charged forms, it is concluded that the CD effect stemmed from supramolecular interactions. The helical structure of the aggregates is indicated by the absorption band around 820 nm which displays a strong bisignate Cotton effect. When the content of ***S*** **‐SQ‐1** in a mix of ***S*** **‐SQ‐1** and **a‐SQ‐1** is increased, the ellipticity that is determined at 868 nm increases predominantly in a linear fashion as shown in Figure 2 c. This correlation suggests that the aggregates formed by ***S*** **‐SQ‐1** and **a‐SQ‐1** in water are either barely mixing or the ability of ***S*** **‐SQ‐1** to bias one helical sense is low.

### Formation of CD active aggregates of *S*‐SQ‐1 and a‐SQ‐1 in the solid state

**Figure 3:**
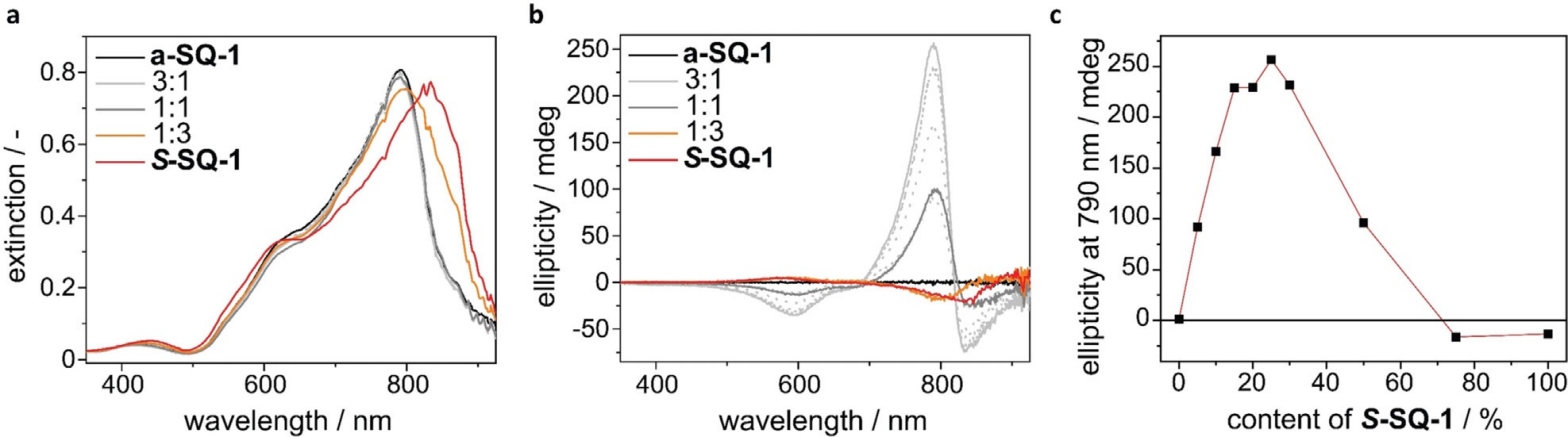
Photophysical properties of thin films prepared from ***S*** **‐SQ‐1** and **a‐SQ‐1** and mixtures thereof. (a) UV/Vis spectra; (b) CD spectra; (c) Variation of the ellipticity with increasing content of ***S*** **‐SQ‐1** determined at 790 nm. All thin films have a thickness of approximately 40 nm.

The chiroptical properties of ***S*** **‐SQ‐1** and **a‐SQ‐1** were further investigated in bulk. Thin films (thickness =40 nm) were prepared by spin‐coating a solution of ***S*** **‐SQ‐1** and **a‐SQ‐1** in chloroform onto microscope glass slides. The films were characterised by UV/Vis spectroscopy (Figure 3 a). The UV/Vis spectra obtained from the thin films show noticeable differences from those recorded for the aqueous solutions. In the thin films, both dyes show a broad absorption band with a maximum that is red shifted with respect to the absorption band of the molecularly dissolved species. For **a‐SQ‐1**, this maximum is found at 790 nm and for ***S*** **‐SQ‐1** at 834 nm. Additionally, the absorption spectra of both compounds exhibit a shoulder 620 nm which is in turn blue‐shifted with respect to the absorption band of the molecularly dissolved species. The coinciding presence of a red‐ and blue‐shifted component in the spectra are tentatively attributed to the presence of several molecules with different orientations in one structural repeating unit. Since the shapes of the absorption spectra of ***S*** **‐SQ‐1** and **a‐SQ‐1** are very similar, a structurally similar packing of the chromophores in the aggregates, which are formed in the thin films, is likely. Having investigated the absorption properties of thin films consisting of the pure compounds, we investigated thin films consisting of spin‐coated mixtures of ***S*** **‐SQ‐1** and **a‐SQ‐1**. As shown in Figure 3 a, the absorbance spectra recorded for all thin films, where 5–50 % ***S*** **‐SQ‐1** were present in the mixture showed a maximum of absorbance at 790 nm. The spectra look very similar to the spectrum recorded for pure **a‐SQ‐1**. Increasing the content of ***S*** **‐SQ‐1** to 75 % in the mix results in a red‐shift of the maximum of absorbance to 799 nm. For pure ***S*** **‐SQ‐1**, the red‐shift is even more pronounced and the wavelength of maximum absorbance is determined with 834 nm. These results indicate that thin films, which comprise up to 50 % ***S*** **‐SQ‐1** in a mix of ***S*** **‐SQ‐1** and **a‐SQ‐1**, show an aggregation mode that is structurally very similar to the aggregation of pure **a‐SQ‐1**. The mixtures containing 75 % ***S*** **‐SQ‐1** in contrast shows an aggregation mode that is approaching the aggregate structure formed by pure ***S*** **‐SQ‐1**.

Next, CD spectroscopy was used to investigate the thin films (Figure 3 b). Gratifyingly, thin films consisting of pure **a‐SQ‐1**, do not show any CD effect. In contrast, thin films containing a mixture of ***S*** **‐SQ‐1** and **a‐SQ‐1** or pure ***S*** **‐SQ‐1** show significant Cotton effects in the CD spectrum. As noticed in Figure 3 c, the CD effect is most intense for a 1:3 mix of ***S*** **‐SQ‐1** and **a‐SQ‐1,** and not for pure ***S*** **‐SQ‐1**. This observation is in sharp contrast to the results obtained in water, where the most intense CD‐effect was measured for the sample containing pure ***S*** **‐SQ‐1**. Additional spectroscopic data obtained from the Muller matrix is discussed in the Supporting Information.

The spectroscopic data suggests that **a‐SQ‐1** forms helical aggregates with P‐ and M‐helices as a racemic mixture, as the addition of a small amount of ***S*** **‐SQ‐1** to the feed, induces a CD effect but does not impact the absorbance spectrum of the material. The absence of changes in the absorbance spectra indicate that the aggregate's structure is not changed as well. ***S*** **‐SQ‐1** is structurally incorporated into the aggregates formed by **a‐SQ‐1**. Owing to the chiral information of the stereogenic centres, this incorporation non‐proportionally biases the ratio of the populations of present P‐ and M‐helical aggregates. In helical supramolecular systems, this effect is commonly referred to as the “sergeants‐and‐soldiers” effect.^[55]^ Interestingly, the “sergeants‐and‐soldiers” effect is not operative over the whole range of mixtures.

Once ***S*** **‐SQ‐1** becomes the major fraction in the feed, the recorded absorbance spectrum starts to change, indicative of a changed structure of the formed aggregates. At the same time, the recorded CD effect is decreased. This observed trend is fascinating since it shows that the maximisation of the amount of stereogenic centres in the aggregates—by increasing the amount of ***S*** **‐SQ‐1** in the feed—does not yield the thin film with the highest CD effect. The highest CD effect is achieved when ***S*** **‐SQ‐1** is solely used as a director for **a‐SQ‐1**, rather than a structural component.

In order to elucidate whether the measured ellipticity is caused by purely excitonic coupling or also stems from other effects such as cholesteric ordering, thin films with different thicknesses were prepared by spin coating. As shown in Figure S10, the ratio of measured ellipticity and film thickness was relatively constant for rather thin films (thicknesses between 11 and 24 nm). When the film thickness was increased further, an increase of the ratio was noticed. In very thin films, the CD effect can only originate from the mechanism operative at the nm scale, which suggests the presence of excitonic coupling. The increase of the ratio with increasing film thickness suggests that the recorded CD effect stems partly from cholesteric ordering in bulk. In all thin films, the aggregate size is very small and the films do not show birefringence when investigated by polarised optical microscopy (Figure S19), while only a negligible linear dichroism (LD) effect was recorded (Figure S10d).

**Figure 4:**
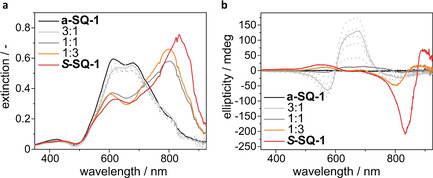
Photophysical properties of annealed (10 min, 210 *°C* under nitrogen atmosphere) thin films prepared from **S‐SQ‐1** and **a‐SQ‐1** and mixtures thereof. (a) UV/Vis spectra; (b) CD spectra.

The absorbance and CD spectra recorded for the thin films differ from the spectra recorded for the aqueous samples. Since the aqueous samples were equilibrated by a heating and cooling cycle, the impact of annealing on the aggregated structures in thin film was probed. All thin films were annealed for 10 min at 210 °C under nitrogen atmosphere which is well below the molecules’ decomposition temperature. Subsequently, the optical properties were investigated again. The absorbance spectra recorded after annealing are depicted in Figure 4 a. The thin films with low ***S*** **‐SQ‐1** content (0–30 %) exhibit a decrease of the former band of maximum absorbance around 790 nm and the rise of two new absorption bands around 600 nm and 700 nm, respectively. For thin films with a higher ***S*** **‐SQ‐1** content (50–100 %), the absorbance of the band around 600 nm intensifies slightly. The position of the maximum of absorbance is retained around 800 nm after annealing. Besides changes in the UV/Vis spectra, the CD spectra recorded for all samples show differences after annealing, too. As depicted in Figure 4 b, samples with low ***S*** **‐SQ‐1** content (0–30 %) showed a shift of the wavelength of maximum CD effect to shorter wavelengths. After annealing, the maximum CD effect was noticed around 690 nm (formerly 790 nm). The CD effect is decreased for the 50 % ***S*** **‐SQ‐1** sample and increased for the 75 % ***S*** **‐SQ‐1** and the pure ***S*** **‐SQ‐1** sample. The changes in both UV/Vis and CD spectra upon annealing suggest therefore that the aggregates formed during spin coating were the result of kinetic effects. The recorded LD spectra are depicted in Figure S11 and indicate that after annealing, LD has negligible contribution to the absorption properties, still.

### Spin‐filtering properties of thin films by mc‐AFM

**Figure 5:**
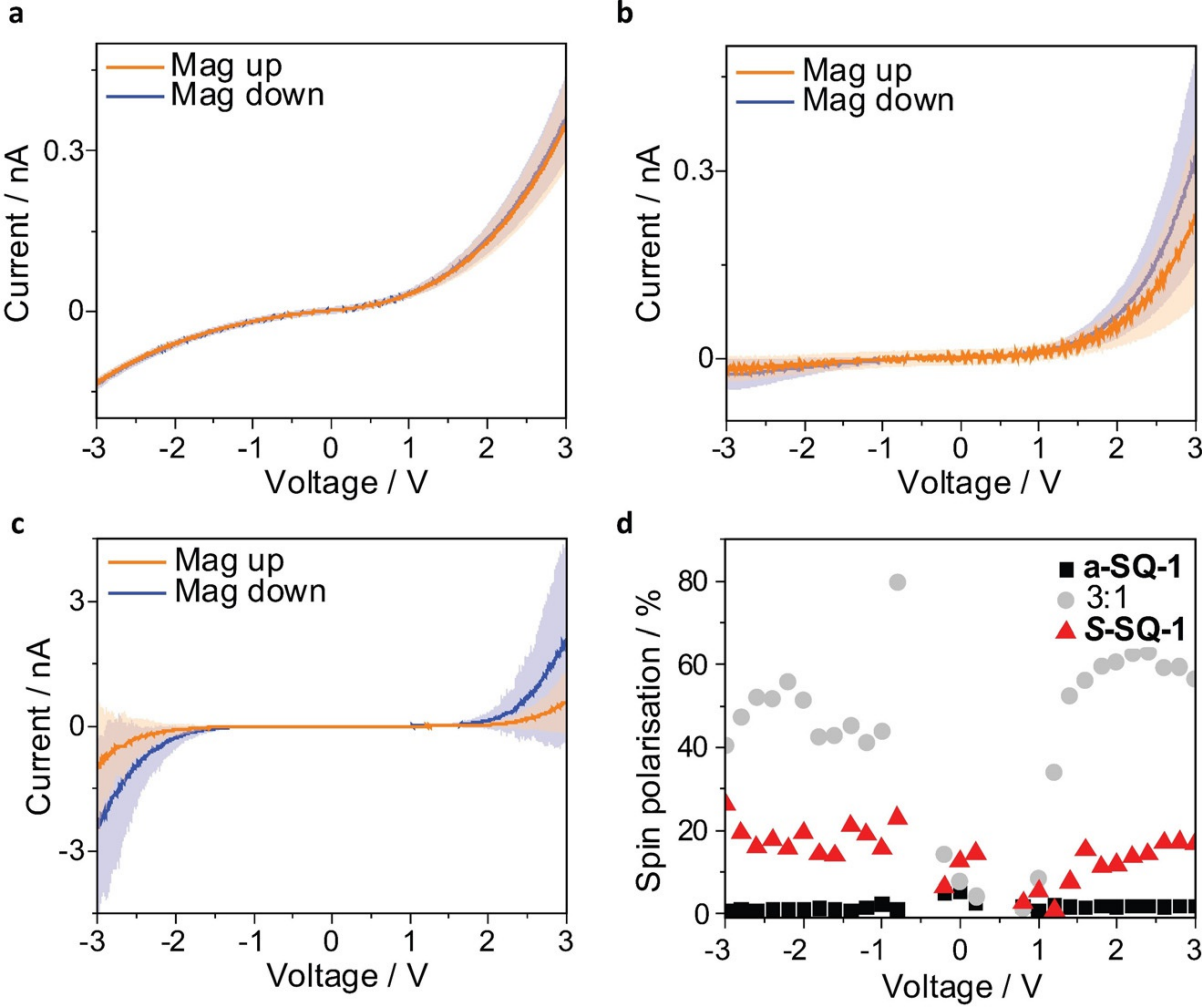
Spin‐dependent conduction through thin films consisting of **a‐SQ‐1** (a), **S‐SQ‐1** (b) and 3:1 mixture thereof (c). The I‐V plots show determined averages and standard deviations over 100 individual measurements. For each sample measurements were performed with the Ni film magnetised with the north pole pointing up (orange) or down (blue). (d) Spin polarisation calculated from the mc‐AFM measurements.

In order to confirm the exceptional non‐linear behaviour found for the spin‐coated films, we performed spin‐filtering experiments. Magnetic‐conductive atomic force microscopy (mc‐AFM) is an efficient method for studying the spin‐selectivity in the electron transport through chiral structures including the effect of the interface between the probed material and the substrate.^[56]^ The electrical measurement records the current transported through a thin film of organic matter deposited on top of a gold‐coated nickel surface. During the measurement, the sample is magnetised with its magnetisation perpendicular to the surface. Owing to the magnetisation of the Ni/Au substrate, the electrons that are injected into the organic material have a bias in their populations of electron spin alignments (spin‐up and spin‐down). It is important to note that the values are determined in the nonlinear regime which make the measurement sensitive to detect the spin polarisation for the electron conduction. The results obtained from recording 100 *I‐V* curves for both orientations of the magnet and the three samples containing pure **a‐SQ‐1**, pure ***S*** **‐SQ‐1** and the 3:1 mixture thereof are depicted in Figure 5. The orientation of the magnet does not have an influence on the I‐V curves recorded for **a‐SQ‐1**. For the sample containing purely ***S*** **‐SQ‐1**, only a small impact is found. The most pronounced dependency of the I‐V curves on the orientation of the magnet is found for the 3:1 mixture of both **a‐SQ‐1** and ***S*** **‐SQ‐1**.

From the recorded measurements, the spin polarisation was determined using $SP=\left[\frac{I_{up}-I_{down}}{I_{up}+I_{down}}\right]•100$ . The spin polarisation at 3 V was determined with 0 % for **a‐SQ‐1**, 17±5 % for ***S*** **‐SQ‐1** and 56±8 % for the 3:1 mixture. As noticed in the Figure 5 d, similar values are found for lower potentials, too. The upper limit of 60 % corresponds to a ratio of about four to one between the two spin states. Various systems, that are based on a homochiral compound only, report a similar spin polarisation, too.[^29^, ^57^, ^58^, ^59^, ^60^, ^61^, ^62^, ^63^] Studies on comparing chiral and achiral materials are reported less often.[^30^, ^31^] Only one very recently reported study investigated the spin polarisation of mixtures of chiral molecules and their achiral counterparts.^[30]^ The compounds were coassembled into supramolecular nanofibers. The spin selective measurements indicated a correlation of the molar circular dichroism of the nanofibers in solution and the spin polarisation which was measured after drop casting and drying. However, both maxima of molar circular dichroism and spin polarisation were found for the sample containing purely chiral material. In the system presented by us, we find the most selective electron transport for materials with the highest optical activity. Since this material does however not contain the highest number of stereogenic centres in the series, our findings indicate a strong correlation between spin polarisation during the electron transport and the optical activity of the organic material. A similar correlation has been found for deoxyribonucleic acid (DNA) based spin filters which showed a drastically reduced spin‐selective electron transport when used as single‐strands instead of double‐strands.^[29]^ Although the number of stereogenic centres in both types of DNA is equal, the formation of a highly‐ordered chiral structure is impaired for the single‐stranded version. With the loss of chiral order, spin selective properties are lost. The system presented by us underlines the cruciality of chiral (supramolecular) order. Although the number of stereogenic centres is increased when comparing the ***S*** **‐SQ‐1** sample with the 3:1 mixture of **a‐SQ‐1** and ***S*** **‐SQ‐1**, a pronounced decrease in spin polarisation is noticed which coincides with the decrease of optical activity.

## Conclusions

The formation of squaraine dyes is commonly achieved by the reaction of squaric acid with aniline‐based precursors. The presented study shows that another class of substrates that is suitable for this type of reaction is given by amide substituted hydroxyl‐benzenes. Both aniline‐ and hydroxyl‐benzene based dyes were prepared in chiral and achiral forms. The dyes exhibit intense colours that can be tuned by the addition of acids or bases. Anilino substituted ***S*** **‐SQ‐1** and **a‐SQ‐1** change their colour from green to violet upon protonation. Blue hydroxyl‐benzene substituted ***S*** **‐SQ‐2** and **a‐SQ‐2** change their colour to red when singly deprotonated.

***S*** **‐SQ‐1** and **a‐SQ‐1** were aggregated in aqueous solution and in thin films. The aggregates were studied by UV/Vis and CD spectroscopy. When mixtures of ***S*** **‐SQ‐1** and **a‐SQ‐1** were aggregated in solution, the CD effect increased predominantly linearly when increasing the amount of chiral ***S*** **‐SQ‐1** in the mix. This linear increase indicates that the aggregates of ***S*** **‐SQ‐1** and **a‐SQ‐1** are either barely mixing or the ability of ***S*** **‐SQ‐1** to bias one helical sense in the mix is low. In contrast, spin‐coated thin films of ***S*** **‐SQ‐1** and **a‐SQ‐1** resulted in co‐assembled structures that displayed a non‐linear increase of the CD effect. Surprisingly, the highest dissymmetry was not measured for a thin film that contained pure ***S*** **‐SQ‐1** but a 1:3 mixture of ***S*** **‐SQ‐1** and **a‐SQ‐1** molecules. The similarity of the absorption spectrum of the 1:3 mixture of ***S*** **‐SQ‐1** and **a‐SQ‐1** with respect to the absorption spectrum of pure **a‐SQ‐1** indicates a very similar aggregation structure for these two samples. Opposed to pure **a‐SQ‐1**, the 1:3 mixture is highly CD active, which in turn suggests that the population between the present P‐ or M‐helices has been altered. Additionally, this sample exhibits a higher CD effect than pure ***S*** **‐SQ‐1**. In contrast to previous studies, the preparation of solid dye structures with the highest CD effect is achieved by using a chiral compound (***S*** **‐SQ‐1**) solely as a director for an achiral compound (**a‐SQ‐1**), rather than a structural component. This nonlinear behaviour was further confirmed by mc‐AFM measurements, as the highest spin selectivity was found for the 1:3 mixture as well. Annealing of the spin‐coated thin films resulted in changes of absorption and CD spectra which suggests the coassembly to be a kinetically controlled process. We note that the degree of circular polarization in absorption displayed by the chiral amide derivates under study is much smaller than observed for the chiral prolinol derivatives of the squaraine chromophore described by Zablocki et al.^[19]^ Furthermore, we note that for crystals of squaraine dyes, the interaction between light and molecules can be exceptionally strong with coupling constants in the order of one eV.^[64]^ This strong coupling, most likely contributes to unconventional optical properties which remain yet to be fully explored.

Considering the emerging work on spintronic applications for example, photoelectrochemical water splitting, where devices functionalised with a chiral, non‐racemic active layer yielded higher currents than for achiral counterparts,[^31^, ^33^] the presented system provides an promising basis to further probe the impact of supramolecular rather than molecular chirality on material properties. Utilisation in spintronic applications will be the topic of future investigations.

## Experimental Section

### Materials and methods

All chemicals were purchased from commercial sources and used without further purification. Dry solvents were obtained with an MBRAUN Solvent Purification System (MB‐SPS). Reactions were followed by thin‐layer chromatography (TLC) using 60‐F254 silica gel plates from Merck and visualised by UV light at 254 nm. Silica column purifications were performed on a Grace Reveleris X2 automated column machine with Reveleris Silica Flash Cartridges and on a Biotage Isolera 1 with Biotage SNAP KP‐SIL columns. ^1^H‐ and ^13^C‐NMR spectra were recorded with a Varian Mercury Vx 400 MHz. Deuterated solvents were purchased from Cambridge Isotope Laboratories and are indicated for each measurement. Chemical shifts (*δ*) are expressed in ppm and are referred to the residual peak of the solvent. Peak multiplicity is abbreviated as s: singlet; d: doublet, q: quartet; p: pentet; m: multiplet; dd: double doublet; dt: double triplet; dq: double quartet. MALDI‐TOF spectra were recorded with Bruker Autoflex Speed using α‐cyano‐4‐hydroxycinnamic acid (CHCA) or *trans*‐2‐[3‐(4‐tert‐butylphenyl)‐2‐methyl‐2‐propenyl‐idene]‐malononitrile (DCBT) as matrix. The stock solutions used for the optical measurements and spin coating were prepared by weighing the necessary amount of compound for the given concentration and transferring into a screw‐capped vial. The desired concentration was adjusted by the addition of solvent using GilsonTM MICROMANTM Positive‐Displacement Pipets. In order to ensure complete dissolution and equilibration during experiments performed in aqueous solution, the samples were heated and stirred at 90 °C for 20 min, followed by slow cooling to room temperature before each measurement. Spectroscopic measurements were performed in high precision cells made of quartz suprasil (Hellma analytics). The optical path length was 10 mm. For spin coating, 50 μL of the solution containing the dye were deposited on microscope glass slides and subsequently spun (Headway Research Inc., conc. 5 mm, 10 mm or 20 mm, spinning speed 1000 rpm or 2000 rpm, 45 s). The microscope glass slides had previously been cleaned by sonication in acetone, demineralised water and isopropanol for 5 min each. For the determination of optical properties after annealing, the spin coated samples were annealed at 210 °C for 10 min under nitrogen atmosphere. UV/Vis, CD and LD spectroscopy were performed on a JASCO J‐815 CD spectrometer either a JASCO Peltier MPTC‐490S temperature controller with a range of 278–373 K or a JASCO Peltier PFD‐425S/15 with a range of 263–383 K. Separate UV/Vis spectra were recorded on a JASCO V‐750 UV/Vis spectrometer. Fluorescence spectroscopy was performed on a PerkinElmer LS 50 B luminescence spectrometer. Optical microscopy using crossed polarisers was performed on an optical microscope from Jenaval. Images were recorded using an Infinity1 camera provided by Lumenera. AFM imaging was performed using an Asylum Research MFP‐3D mounted on an anti‐vibration stage. Silicon tips were supplied by Nanoworld (SIN 89 229F10L1333). The dimensions of the tips were: length of 150 μm, width of 27 μm and thickness of 2.8 μm. The spring constant was 7.4 N m^−1^ and soft tapping‐mode was used to obtain an image. A resolution of 512 points and lines was used for the images. The images were processed using Gwyddion (v. 2.53) software. mc‐AFM measurements were performed on 10 nm thick thin films deposited by spin coating from a chloroform solution on top of a gold‐coated nickel surface (Ni/Au 120/8 nm thicknesses). During the AFM measurement (contact mode), the substrate was magnetised with the magnetic pole up or down by external magnetic field of 0.5 T. The AFM set‐up was a custom‐designed RHK machine capable of reaching a magnetic field of 1 T. The AFM cantilevers were platinum‐coated silicon tips supplied by Micromasch (HQ:DPE‐XSC11, spring constant: 2.7 N m^−1^). During each measurement, an 8–10 nn force was exerted with the tip onto the probed surface. The AFM tip is grounded while the potential at the Au/Ni substrate was varied from −3 to +3 V. Reflectivity and Muller matrix spectroscopy was performed on a W‐VASE ellipsometer from J.A. Wollam.

### Syntheses

We synthesised the substrates for ***S*** **‐SQ‐1** and **a‐SQ‐1** as well as ***S*** **‐SQ‐2** and **a‐SQ‐2** according to literature procedures.[^39^, ^50^, ^51^, ^52^] A detailed summary is given in the Supporting Information. The squaraine dye formations were performed using a modification of the original procedure by Sprenger and Ziegenbein.[^16^, ^35^]

***a‐SQ‐1***: A flask filled with 80 mL of 50/50 *n*‐propanol/toluene was fitted with a Dean–Stark trap with cooler and heated to 110 °C. After the trap was fully filled with solvent and manually emptied once, squaric acid (218 mg, 1.9 mmol, 1 equiv.) was added. Heating and stirring were continued for 15 minutes before *N‐(*3‐(dimethylamino)phenyl)octanamide (1.0 g, 3.8 mmol, 2 equiv.) was added. The solution turned dark green and heating with stirring was continued. After 20 hours of reaction, the mixture had a deep turquoise colour. The reaction mixture was cooled down and the solvent was removed by rotary evaporation to afford a green crude product (1.08 g, 1.79 mmol, 89 %). The crude product was recrystallised from acetonitrile to afford pure **a‐SQ‐1** in high yield (0.97 g, 1.61 mmol, 85 %). ^1^H NMR (400 MHz, Chloroform‐d): *δ*=12.04 and 11.94 (s, 2 H), 8.49 and 8.43 (d, *J*=9.2 Hz, 2 H), 8.25 and 8.22 (d, *J*=2.4 Hz, 2 H), 6.45–6.42 (m, 2 H), 3.18 (s, 12 H), 2.62 and 2.56 (t, *J*=7.2 Hz, 4 H), 1.76 (quin, *J*=7.2 Hz, 4 H), 1.46–1.29 (m, 16 H), 0.876 ppm (t, *J*=6.0 Hz, 6 H); ^13^C NMR (101 MHz, Chloroform‐d): *δ*=182.74, 182.53 and 181.52 (2C), 175.86 and 175.33 (2C), 174.19 and 173.87 (2C), 156.88 (2C), 144.09 (2C), 133.95 and 133.30 (2C), 112.76 and 112.55 (2C), 108.64 (2C), 102.48 (2C), 40.63 (4C), 38.18 and 38.01 (2C), 31.93 and 31.89 (2C), 29.62, 29.40, 29.36 and 29.33 (4C), 25.42 and 25.42 (2C), 22.87 (2C), 14.53 ppm (2C); MALDI/TOF found 602.42 *m*/*z* (calculated 602.38); FT‐IR (cm^−1^): 3135 (w), 3096 (w), 2953 (w), 2918 (m), 2872 (w), 2852 (m), 2808 (w), 2676 (w), 1706 (m), 1609 (s), 1578 (s), 1535 (m), 1483 (m), 1458 (m), 1389 (s), 1352 (s), 1278 (s), 1242 (s), 1207 (s), 1179 (s), 1154 (s), 1125 (s), 1090 (s), 1063 (s), 958 (m), 944 (m), 891 (s), 853 (s), 821 (s) 804 (m), 781 (s), 725 (m), 707 (m), 684 (m), 663 (m), 641 (m), 593 (w), 565 (w), 524 (s) 500 (s), 461 (s).

**S*‐SQ‐1***: A flask filled with 100 mL of 50/50 *n*‐propanol/toluene was fitted with a Dean–Stark trap with cooler and heated to 110 °C. After the trap was fully filled and manually emptied twice, squaric acid (99.7 mg, 0.86 mmol, 1 equiv.) was added. After 30 minutes, (*S*)‐*N‐(*3‐(dimethylamino)phenyl)‐3,7‐dimethyloctanamide (0.51 g, 1.7 mmol, 2 equiv.) was added to the solution which turned dark green upon addition. After 20 h of reaction, the mixture had a deep turquoise colour. The reaction mixture was cooled down and the solvent was removed by rotary evaporation to afford a green crude product (507 mg, 0.77 mmol, 45 %). The crude product was recrystallised from acetonitrile to afford pure ***S*** **‐SQ‐1** in high yield (456 mg, 0.69 mmol, 81 %). ^1^H NMR (400 MHz, Chloroform‐d): *δ*=12.08 and 11.94 (s, 2 H), 8.55 and 8.49 (d, 9.2 Hz, 2 H), 8.31 and 8.30 (d, 2.4 Hz, 2 H), 6.48 (dd, *J*=9.2 Hz, *J*=2.4 Hz, 2 H), 3.22 (s, 12 H), 2.66–2.53 (m, 2 H), 2.45–2.37 (m, 2 H), 2.14 (br, 2 H), 1.59–1.47 (m, 2 H), 1.45–1.14 (m, 12 H), 1.03 (d, *J*=6.6 Hz, 6 H), 0.88–0.84 ppm (m, 12 H); ^13^C NMR (101 MHz, Chloroform‐d): *δ*=182.74 and 181.68 (2C), 176.03 and 175.57 (2C), 173.85 and 173.47 (2C), 156.95 (2C), 144.09 and 143.98 (2C), 134.00 and 133.38 (2C), 112.80 and 112.59 (2C), 108.73 (2C), 102.60 (2C), 45.91 and 45.65 (2C), 40.67 (4C), 39.23, 37.37 and 37.23 (4C), 30.86 and 30.83 (2C), 28.15 (2C), 25.08 and 25.01 (2C), 22.87, 22.85, 22.75 and 22.71 (4C), 20.07 and 19.96 ppm (2C); MALDI/TOF found 658.47 *m*/*z* (calculated 658.45); FT‐IR (cm^−1^): 3133 (w), 2953 (m), 2925 (m), 2868 (m), 2681 (w), 1710 (w), 1607 (s), 1574 (s), 1532 (m), 1484 (w), 1455 (m), 1412 (w), 1382 (m), 1354 (s), 1325 (s), 1277 (s), 1241 (s), 1195 (s), 1139 (s), 1124 (s), 1063 (s), 902 (s), 879 (s), 862 (s), 812 (s), 781 (s), 725 (s), 681 (m), 664 (m), 649 (m), 556 (w), 520 (s), 498 (s), 463 (m).

***a‐SQ2***: A flask filled with 50 mL of 50/50 *n*‐propanol/toluene was fitted with a Dean–Stark trap with cooler and heated to 110 °C. Squaric acid (114 mg, 1.0 mmol, 1 equiv.) was added and heating was continued for three hours. *N‐(*3,5‐Dihydroxyphenyl)octanamide (510 mg, 2.0 mmol, 2 equiv.) was added. The mixture was heated and stirred for 20 h. The reaction mixture formed a deep blue colour, was cooled down and concentrated until dryness. The dark blue residue was dispersed in 30 mL ethyl acetate by sonication. After extraction with 20 mL 0.5 m aqueous hydrochloric acid solution a dark blue solid was filtered off the organic layer. The obtained product was dried in vacuum and characterised without further purification. **a‐SQ‐2** was obtained in 60 % yield (350 mg, 0.6 mmol). ^1^H NMR (400 MHz, [D_6_]DMSO): *δ*=12.46 (br, 2 H), 11.26 (s, 2 H), 7.58 (s, 2 H), 6.00 (s, 2 H), 2.40 (t, *J*=7.4 Hz, 4 H), 1.65–1.55 (m, 4 H), 1.29–1.25 (m, 16 H), 0.86–0.83 ppm (m, 6 H). MALDI/TOF found 580.30 *m*/*z* (calculated 580.28). FT‐IR (cm^−1^): 2954 (m), 2922 (m), 2854 (m), 2625 (m), 1681 (w), 1583 (s), 1516 (m), 1455 (m), 1427 (m), 1383 (m), 1322 (m), 1226 (m), 1191 (s), 1174 (s), 1135 (s), 1123 (s), 1099 (m), 1024 (s), 948 (m), 866 (m), 801 (s), 760 (s), 723 (s), 659 (m), 619 (m), 598 (m), 538 (m), 486 (s). Elemental analysis: found 66.10 % C, 6.91 % H, 4.81 % N, 21.84 % O (calculated: 66.19 % C, 6.94 % H, 4.82 % N, 22.04 % O).

**S*‐SQ‐2***: A flask filled with 50 mL of 50/50 *n*‐propanol/toluene was fitted with a Dean‐Stark trap with cooler and heated to 110 °C. Squaric acid (19 mg, 0.17 mmol, 1 equiv.) was added and heating was continued for three hours. (*S*)‐*N‐(*3,5‐dihydroxyphenyl)‐3,7‐dimethyloctanamide (100 mg, 0.36 mmol, 2.1 equiv.) was added. The mixture was heated and stirred for 20 h. The reaction mixture formed a deep blue colour, was cooled down and concentrated until dryness. The dark blue residue was dispersed in 40 mL ethyl acetate by sonication. After extraction with 20 mL 0.5 m aqueous hydrochloric acid solution a dark blue solid was filtered off the organic layer. The obtained product was dried in vacuum and characterised without further purification. ***S*** **‐SQ‐2** was obtained in 11 % yield (12 mg, 0.02 mmol). The low yield was caused by the small scale of the reaction. ^1^H NMR (400 MHz, [D_6_]DMSO): *δ*=12.41 (br, 2 H), 11.26 (s, 2 H), 7.58 (s, 2 H), 5.97 (s, 2 H), 2.41 (dd, *J*=14.2, 6.4 Hz, 2 H), 2.23 (dd, *J*=14.3, 7.6 Hz, 2 H), 1.98 (br, 2 H), 1.54–1.44 (m, 2 H), 1.35–1.09 (m, 12 H), 0.92 (d, *J*=6.7 Hz, 6 H), 0.83 ppm (d, *J*=6.6 Hz, 12 H). MALDI/TOF found 659.34 *m*/*z* (calculated [M]+Na^+^ 659.33). FT‐IR (cm^−1^): 2955 (m), 2928 (m), 2868 (m), 2620 (br), 1682 (w), 1610 (m), 1583 (s), 1517 (m), 1458 (w), 1426 (m), 1383 (m), 1325 (w), 1229 (s), 1196 (s), 1174 (s), 1125 (s), 1024 (s), 953 (m), 866 (m), 806 (m), 770 (m), 723 (s), 659 (w), 616 (m), 598 (m), 535 (m), 486 (m). Elemental analysis: found 66.91 % C, 7.47 % H, 4.29 % N, 19.84 % O (calculated: 67.90 % C, 7.60 % H, 4.40 % N, 20.10 % O).

## Conflict of interest

The authors declare no conflict of interest.

## Supporting information

As a service to our authors and readers, this journal provides supporting information supplied by the authors. Such materials are peer reviewed and may be re‐organized for online delivery, but are not copy‐edited or typeset. Technical support issues arising from supporting information (other than missing files) should be addressed to the authors.

Supplementary
