# Supplementary material for: Helicity Control in the Aggregation of Achiral Squaraine Dyes in Solution and Thin Films
Source: Chemistry. 2020 Nov 9;27(1):298–306. doi: 10.1002/chem.202002695 (PMC7839690; doi:10.1002/chem.202002695)
Supplement: Supplementary file 1 — Supplementary [file CHEM-27-298-s001.pdf]

Chemistry–A European Journal

Supporting Information

Helicity Control in the Aggregation of Achiral Squaraine Dyes in Solution and Thin Films

Andreas T. Rösch,[a] Qirong Zhu,[b] Jorn Robben,[a] Francesco Tassinari,[b] Stefan C. J. Meskers,[c] Ron Naaman,[b] Anja R. A. Palmans,[a] and E. W. Meijer*[a]

Table of Contents

Synthesis: 2

Huang-Rhys parameter *S* – calculation 13

Photophysical properties – steady state 15

Photophysical properties changed by addition of acid or base 19

Blue dyes *S*-SQ-2 and a-SQ-2: chemical stability: 22

Green dyes *S*-SQ-1 and a-SQ-1: supporting information on aggregation 23

Müller matrix spectroscopy 29

Green dyes *S*-SQ-1 and a-SQ-1: POM images of spin coated thin films 35

Green dyes *S*-SQ-1 and a-SQ-1: AFM images of spin coated thin films 36

Blue dyes S-SQ-2 and a-SQ-2: supporting information on aggregation 37

Recorded spectra 40

Nuclear magnetic resonance 40

Fourier-transform infrared spectroscopy 51

Matrix assisted laser desorption/ionisation time-of-flight mass spectrometry 54

Literature 58

Synthesis:

The synthesis was performed according to Scheme S1. The individual steps are described in detail in the following section.

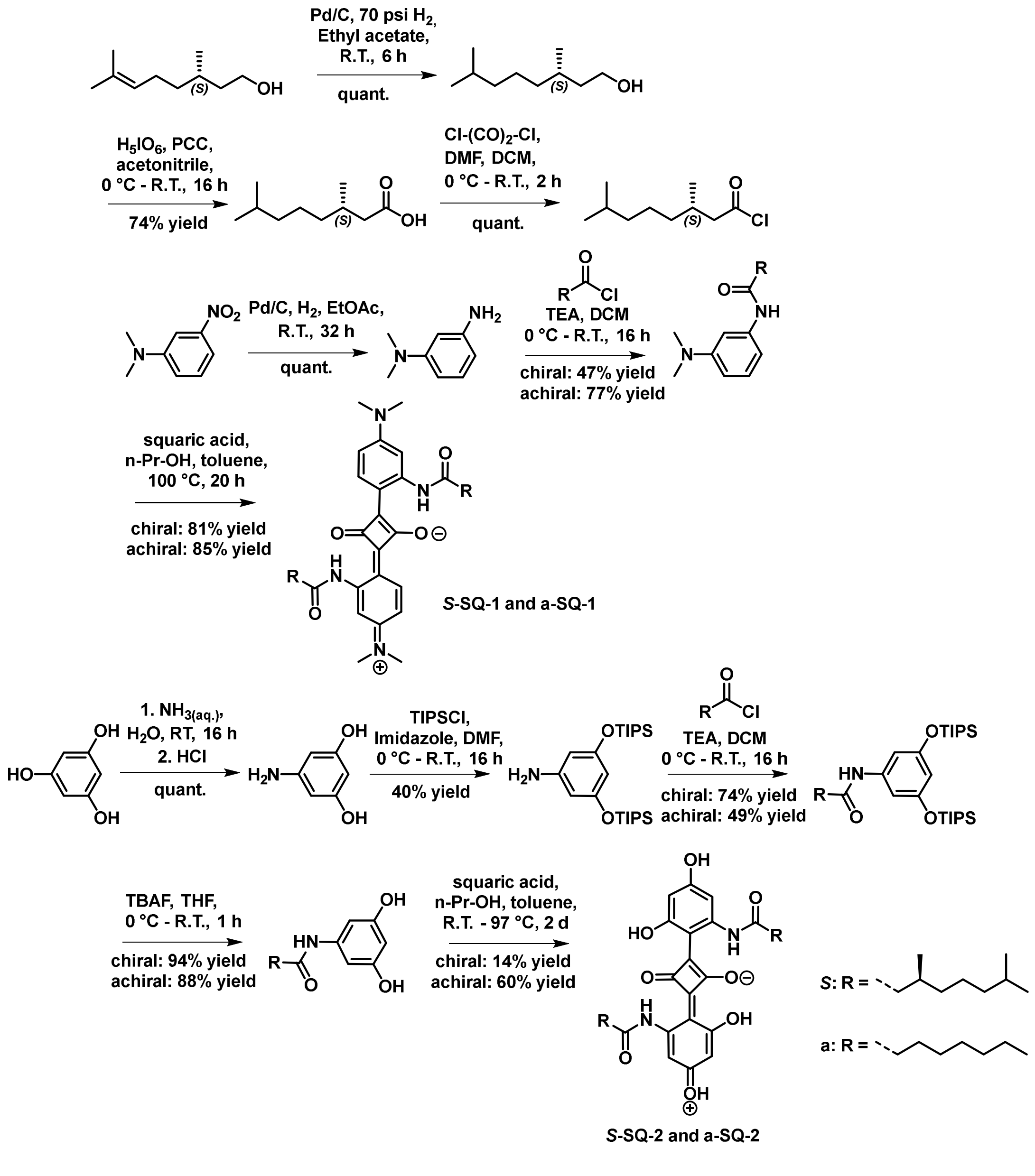

Scheme S1: Reaction scheme for synthesizing ***S*-SQ-1** and **a-SQ-1** as well as ***S*-SQ-2** and **a-SQ-2**. Squaric acid was reacted with custom synthesised amides to yield ***S*-SQ-1** and **a-SQ-1** as green and ***S*-SQ-2** and **a-SQ-2** as blue solids.

Synthesis of [1] (*S*)-3,7-dimethyloctan-1-ol

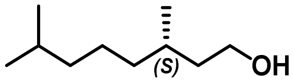

(*S*)-Citronellol (20.0 g, 0.13 mol) was dissolved in 150ml ethyl acetate. Argon was bubbled through the solution for 15 minutes before Pd/C (0.25 g, 10%) was added. The vessel was inserted into a Parr hydration apparatus and the hydrogen pressure was adjusted to 70 psi. The reaction was started by shaking the vessel and a decreasing hydrogen pressure was noticed. During the following three hours of shaking, the hydrogen pressure had to be readjusted repeatedly. Having shaken the vessel at room temperature in the hydrogen atmosphere for 6 h in total, the catalyst was removed by filtration through diatomaceous earth. The filter was washed with ethyl acetate. Having removed the solvent by reduced pressure, the pure product was obtained as a clear oil. (*S*)-3,7-Dimethyloctan-1-ol was obtained in quantitative yield (20.6 g, 0.13 mol). ^{1}H NMR (400 MHz, Chloroform-d): δ [ppm] = 3.75 – 3.64 (m, 2H), 1.69 – 1.07 (m, 10H), 0.90 (d, J = 6.6 Hz, 3H), 0.87 (d, J = 6.7 Hz, 6H).

Synthesis of [2] (*S*)-3,7-dimethyloctanoic acid

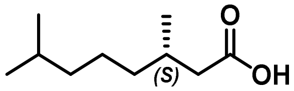

Periodic acid (15.85 g, 69.5 mmol, 2.2 eq.) was dissolved in 250 mL acetonitrile and cooled with an ice bath. Then, (*S*)-3,7-dimethyloctan-1-ol (5 g, 31.6 mmol, 1 eq.) was dissolved in 40 mL acetonitrile and added dropwise to the cooled solution of periodic acid. After pyridinium chlorochromate (136 mg, 0.6 mmol, 0.02 eq.) had been added, the reaction mixture turned yellow. Stirring was continued for 15 min at 0 °C and subsequently for 16 h at room temperature. The solution was concentrated, diluted in 500 mL ethyl acetate and extracted with 250 mL of a water/ brine solution (v/v = 1/1), 250 mL saturated aqueous $NaHSO_3$ solution and 250 mL brine. The organic layer was dried with $MgSO_4$, filtered and the filter cake was washed with ethyl acetate. The organic phase was concentrated by reduced pressure. The crude product was purified by Kugelrohr distillation (1 mbar, 120 °C). (*S*)-3,7-dimethyloctanoic acid was obtained in 74% yield (4.01 g, 23.3 mmol). ^{1}H NMR (400 MHz, Chloroform-d): δ [ppm] = 2.35 (dd, J = 15.0, 5.9 Hz, 1H), 2.15 (dd, J = 14.9, 8.2 Hz, 1H), 2.02 – 1.91 (m, 1H), 1.57 – 1.47 (m, 1H), 1.39 – 1.11 (m, 6H), 0.97 (d, J = 6.7 Hz, 3H), 0.87 (d, J = 6.6 Hz, 6H).

Synthesis of [3] *N,N*-dimethylbenzene-1,3-diamine

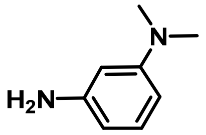

N,N-Dimethyl-3-nitroaniline (2.56 g, 15 mmol) was dissolved in 100 ml of ethyl acetate. The orange solution was bubbled through with argon for 15 minutes. Then Pd/C (30.2 mg, 10%) was added to the solution. The reaction mixture flask was pressurised with hydrogen gas (5 bar) for 32 hours. The colourless reaction mixture was filtered through diatomaceous earth. The filtrate's solvent was removed by rotary evaporation to afford an orange liquid. The obtained *N,N*-dimethylbenzene-1,3-diamine (2.04 g, 15 mmol, 100%) was used for the following reaction without further purification. ^{1}H NMR (400 MHz, Chloroform-d): δ [ppm] = 7.03 (t, J = 8.0 Hz, 1H), 6.20 (dd, J = 8.5, 2.1 Hz, 1H), 6.13 – 6.06 (m, 2H), 3.58 (br, 2H), 2.91 (s, 6H).

Synthesis of [a-4] *N*-(3-(dimethylamino)phenyl)octanamide

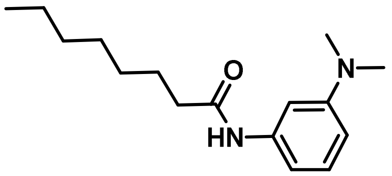

N,N-Dimethylbenzene-1,3-diamine (2.00 g, 15 mmol) and triethylamine (2.66 mL, 19 mmol) were added to 50 mL dry dichloromethane under argon atmosphere. After the solution was cooled to 0°C, octanoyl chloride (2.67 g, 16 mmol) was added. A precipitate formed upon the addition. The reaction mixture was allowed to warm up to room temperature and was further stirred for 16 hours. The reaction mixture was extracted three times with 25 mL water, four times with 25 mL of a one molar aqueous solution of sodium hydroxide solution and finally twice 25 mL with brine. The organic phase was dried with $MgSO_4$, filtered and the solvent was removed by rotary evaporation to obtain *N*-(3-(dimethylamino)phenyl)octanamide in 77% yield (3.29 g, 12.5 mmol). ^{1}H NMR (400 MHz, Chloroform-d): δ [ppm] = δ 7.17 – 7.13 (m, 2H), 7.08 (br, 1H), 6.69 (d, J = 7.9 Hz, 1H), 6.49 – 6.46 (m, 1H), 2.94 (s, 6H), 2.33 (t, J = 7.6 Hz, 2H), 1.72 (q, J = 7.4 Hz, 2H), 1.34 – 1.28 (m, 8H), 0.87 (t, J = 6.8 Hz, 3H).

Synthesis of [*S*-4] *(S)-N*-(3-(dimethylamino)phenyl)-3,7-dimethyloctanamide

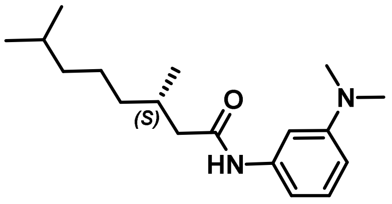

(*S*)-3,7-Dimethyloctanoic acid (2.83 g, 16.7 mmol) was dissolved in dry dichloromethane (40 mL) in dried glassware under argon. After the solution was cooled to 0°C, oxalyl chloride (2.91 g, 23 mmol) and *N,N*-dimethylformamide (0.2 mL, cat.) were added to the solution. The reaction mixture was then allowed to warm up to room temperature and reacted for 1.5 hours. The reaction mixture was distilled at 65°C under 1 atm. The residue was redissolved in dry dichloromethane and added dropwise to a cooled solution of *N,N*-dimethyl-3-nitroaniline (2.09 g, 15 mmol) and triethylamine (2.35 mL, 17 mmol) in dry dichloromethane (40 mL) under argon. After 60 hours, the reaction mixture was extracted three times with brine and twice with water. The organic phase was dried with $MgSO_4$, filtered and the solvent was removed by rotary evaporation to afford 4.34 g of black crude product. The crude product was purified on a silica column (heptane - ethyl acetate, 0-50%) to afford *(S)-N*-(3-(dimethylamino)phenyl)-3,7-dimethyloctanamide as viscous beige oil in 47% yield (2.09 g, 7.1 mmol). ^{1}H NMR (400 MHz, Chloroform-d): δ [ppm] = 7.15 (t, J = 8.1 Hz, 2H), 7.02 (br, 1H), 6.69 (d, J = 7.2 Hz, 1H), 6.48 (dd, J = 8.3, 2.0 Hz, 1H), 2.95 (s, 6H), 2.37 – 2.32 (m, 1H), 2.12 – 2.07 (m, 2H), 1.51 (sept, J = 6.6 Hz, 1H), 1.37 – 1.15 (m, 6H), 0.99 (d, J = 6.2 Hz, 3H), 0.86 (d, J = 7.6 Hz, 6H).

Synthesis of [5] 5-aminobenzene-1,3-diol hydrochloride

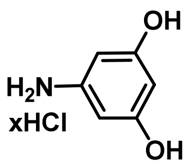

Argon gas was bubbled through 40 mL aqueous ammonia solution for 15 min. The solution was cooled to 0 °C before 8 g phloroglucinol dihydrate (49 mmol, 1.0 eq.) was added. The mixture was allowed to warm up to room temperature and was stirred for 16 h under argon atmosphere. The turbid solution was concentrated by distillation under reduced pressure until ca. 20 mL water had been removed. 20 mL 3 N $HCl_{(aq.)}$ (63 mmol, 1.3 eq.) was added and the dispersion was concentrated until dryness. 5-Aminobenzene-1,3-diol hydrochloride (7.8 g, 48.3 mmol) was obtained as a yellowish solid (99% yield) and used without

purification in the following reaction. ^1H NMR (400 MHz, DMSO-d_6): δ [ppm] = 10.02 (s, 3H), 7.47 – 7.09 (m, 1H), 6.28 – 6.22 (m, 2H).

Synthesis of [6] 3,5-bis((triisopropylsilyl)oxy)aniline

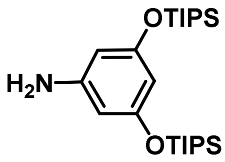

The reaction was carried out under inert conditions in dried glassware under argon. 5-Aminobenzene-1,3-diol hydrochloride (3.72 g, 23 mmol, 1 eq.) and imidazole (6.0 g, 88 mmol, 3.8 eq.) were dissolved in 25 mL dry *N,N*-dimethylformamide. The mixture was cooled to 0 °C and triisopropylsilyl chloride (12.3 mL, 58 mmol, 2.5 eq.) was added. The reaction mixture was allowed to warm up to room temperature and stirring was continued for 16 h. The reaction was stopped by addition of an aqueous saturated NH_4Cl solution (20 mL). The mixture was extracted twice with 50 mL ethyl acetate. The combined organic layers were washed with 50 mL water and 50 mL brine and finally dried with $MgSO_4$. The yellowish solution was filtrated and the filter cake was washed with ethyl acetate. Concentration by rotary evaporation yielded crude product which was purified by silica column chromatography with chloroform (20-50%) in heptane as eluent. Pure 3,5-bis((triisopropylsilyl)oxy)aniline was obtained as yellow oil in 40% yield (4.0 g, 9.2 mmol). ^1H NMR (400 MHz, Chloroform-d): δ [ppm] = 5.87 (s, 3H), 3.52 (s, 2H), 1.26 – 1.19 (m, 6H), 1.09 (d, J = 7.2 Hz, 36H).

Synthesis of [a-7] *N*-(3,5-bis((triisopropylsilyl)oxy)phenyl)octanamide

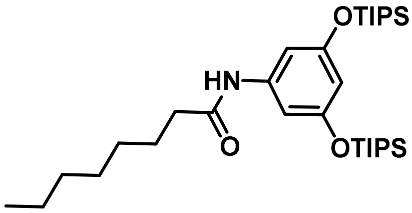

The reaction was carried out under inert conditions in dried glassware under argon. Caprylic acid (2.16 g, 15 mmol, 1 eq.) were dissolved in 30 mL dry dichloromethane. The solution was cooled to 0 °C and 0.96 mL oxalyl chloride (16.5 mmol, 1.1 eq.) and 57 µL *N,N*-dimethylformamide (0.75 mmol, 0.05 eq.) were added. The reaction mixture was allowed to warm up to room temperature again and stirred for two hours before the solvent was removed by distillation under reduced pressure. The crude product was dissolved in 15 mL dry dichloromethane and added dropwise to a cool solution of 3,5-

bis((triisopropylsilyl)oxy)aniline (6.2 g, 15 mmol, 1.0 eq.) and triethylamine (2.17 mL, 16.5 mmol, 1.1 eq.) in dry dichloromethane. The reaction mixture was allowed to warm up to room temperature again and stirred for 16 h. The reaction mixture was extracted with 25 mL brine, 25 mL water and dried with $MgSO_4$. The mixture was filtered and the filter cake was washed with dichloromethane. Subsequently, the obtained solution was concentrated. Purification was performed by silica column chromatography with chloroform (0-100%) in heptane as eluent. The pure product was obtained as white solid in 49% yield (49%, 7.25 mmol). ^{1}H NMR (400 MHz, Chloroform-d): δ [ppm] = 6.91 (s, 1H), 6.74 (s, 2H), 6.18 (s, 1H), 2.30 (t, J = 7.6 Hz, 2H), 1.74 – 1.67 (s, 2H), 1.37 – 1.08 (m, 42H), 0.88 (t, J = 6.8 Hz, 3H).

Synthesis of [*S*-7] *N*-(3,5-bis((triisopropylsilyl)oxy)phenyl)octanamide

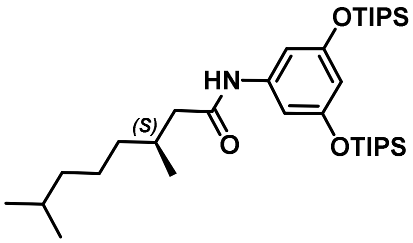

The reaction was carried out under inert conditions in dried glassware under argon. (*S*)-3,7-Dimethyloctanoic acid (1.59 g, 9.2 mmol, 1 eq.) was dissolved in 20 mL dry dichloromethane. The solution was cooled to 0 °C and oxalyl chloride (0.87 mL, 10.1 mmol, 1.1 eq.) as well as *N,N*-dimethylformamide (35 µL, 0.46 mmol, 0.05 eq.) were added. The reaction mixture was allowed to warm up to room temperature again and stirred for two hours before the solvent was removed by distillation under reduced pressure. The crude product was dissolved in 10 mL dry dichloromethane and added dropwise to a cool solution of 3,5-bis((triisopropylsilyl)oxy)aniline (4 g, 9.1 mmol, 1.0 eq.) and triethylamine (1.42 mL, 10.2 mmol, 1.1 eq.) in 20 mL of dry dichloromethane. The reaction mixture was allowed to warm up to room temperature again and stirred for 16 h. The reaction mixture was extracted with 25 mL brine and 25 mL water. The organic phase was dried with $MgSO_4$. The mixture was filtered and the filter cake was washed with dichloromethane. Subsequently, the obtained solution was concentrated to yield crude (*S*)-*N*-(3,5-bis((triisopropylsilyl)oxy)phenyl)-3,7-dimethyloctanamide. Purification was performed by silica column chromatography with dichloromethane (20-60%) in heptane as eluent. The pure product was obtained as white solid in 74% yield (3.98 g, 6.7 mmol). ^{1}H NMR (400 MHz, Chloroform-d): δ [ppm] = 6.90 (s, 1H), 6.74 (s, 2H), 6.18 (s, 1H), 2.32 (q, J = 9.4 Hz, 1H), 2.06 (q, J = 7.9 Hz, 2H), 1.52 – 1.46 (m, 1H), 1.35 - 1.08 (m, 46H), 0.98 (d, J = 5.9 Hz, 2H), 0.90 – 0.85 (m, J = 19.4 Hz, 9H).

Synthesis of [a-8] *N*-(3,5-dihydroxyphenyl)octanamide

The reaction was carried out under inert conditions in dried glassware under argon. *N*-(3,5-bis((triisopropylsilyl)oxy)phenyl)octanamide (4.04 g, 7.1 mmol, 1 eq.) were dissolved in 50 mL dry tetrahydrofuran. The solution was cooled to 0 °C before 22 mL tetra-*n*-butylammoniumfluoride (1M in tetrahydrofuran, 22 mmol, 3 eq.) were added. The reaction mixture was allowed to warm up to room temperature again and stirred for three hours. The obtained solution was diluted with 50 mL ethyl acetate and extracted with 20 mL saturated aqueous NH_4Cl solution, 20 mL brine and 20 mL water. The organic phase was dried with $MgSO_4$, filtered and the filter cake was washed with ethyl acetate. Subsequently, the obtained solution was concentrated to yield crude product. Purification was performed by silica column chromatography with methanol (0-10%) in dichloromethane as eluent. The pure product was obtained as white solid in 88% yield (1.57 g, 6.2 mmol). ^1H NMR (400 MHz, DMSO-d_6): δ [ppm] = 9.53 (s, 1H), 9.10 (s, 2H), 6.55 (d, J = 2.1 Hz, 2H), 5.87 (t, J = 2.2 Hz, 1H), 2.23 (t, J = 7.4 Hz, 2H), 1.58 – 1.525 (m, 2H), 1.32 – 1.20 (m, 8H), 0.85 (t, J = 7.0 Hz 3H).

Synthesis of [*S*-8] (*S*)-N-(3,5-dihydroxyphenyl)-3,7-dimethyloctanamide

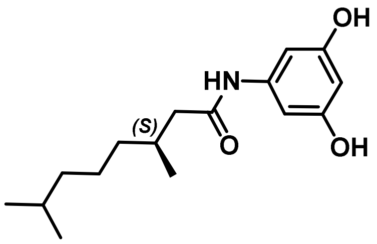

The reaction was carried out under inert conditions in dried glassware under argon. (*S*)-*N*-(3,5-Bis((triisopropylsilyl)oxy)phenyl)-3,7-dimethyloctanamide (1.02 g, 1.73 mmol, 1 eq.) was dissolved in 20 mL dry tetrahydrofuran. The solution was cooled to 0 °C before tetra-*n*-butylammoniumfluoride (1 M in tetrahydrofuran, 5.2 mL, 5.19 mmol, 3 eq.) was added. The reaction mixture was allowed to warm up to room temperature again and stirred for three hours. The obtained solution was diluted with 25 mL ethyl acetate and extracted with 10 mL saturated aqueous NH_4Cl solution, 10 mL brine and 10 mL water. The organic phase was dried with $MgSO_4$, filtered and the filter cake was washed with ethyl acetate. Subsequently, the obtained solution was concentrated to yield crude (*S*)-*N*-(3,5-bis((triisopropylsilyl)oxy)phenyl)-3,7-dimethyloctanamide. Purification was performed by silica column chromatography with methanol (0-20%) in chloroform as eluent. The pure product was obtained as white solid in 94% yield (455 mg, 1.6 mmol). ^1H NMR (400 MHz, Acetone-d6): δ [ppm] = 8.86 (s, 1H), 8.13 (s,

1H), 6.76 (d, J = 2.1 Hz, 2H), 6.06 (t, J = 2.2 Hz, 1H), 2.30 (dd, J = 13.8, 6.0 Hz, 1H), 2.13 – 2.08 (m, 1H), 2.00 – 1.98 (m, 1H), 1.58 – 1.48 (m, 1H), 1.40 – 1.1 (m, 6H), 0.94 (d, J = 6.6 Hz, 3H), 0.86 (d, J = 6.6 Hz, 6H).

Synthesis of a-SQ-1

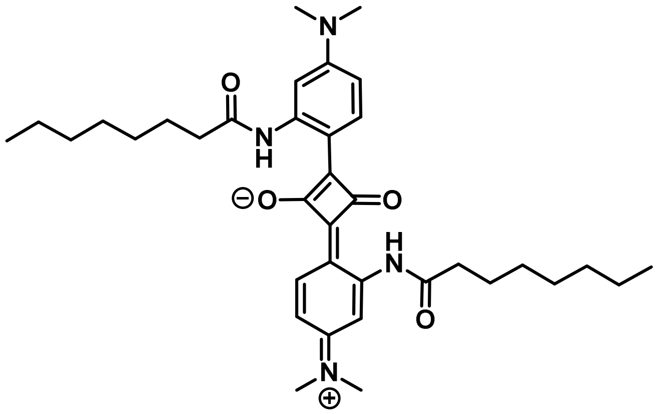

A flask filled with 80 mL of 50/50 *n*-propanol/toluene was fitted with a Dean-Stark trap and cooler and heated to 110 °C. After the trap was fully filled with solvent and manually emptied once, squaric acid (218 mg, 1.9 mmol, 1 eq.) was added. Heating and stirring were continued for 15 minutes before *N*-(3-(dimethylamino)phenyl)octanamide (1.0 g, 3.8 mmol, 2 eq.) was added. The solution turned dark green and heating and stirring were continued. After 20 hours of reaction, the mixture had a deep turquoise colour. The reaction mixture was cooled down and the solvent was removed by rotary evaporation to afford a green crude product (1.08 g, 1.79 mmol, 89%). The crude product was recrystallised from acetonitrile to afford pure **a-SQ-1** in high yield (0.97 g, 1.61 mmol, 85%). ^{1}H NMR (400 MHz, Chloroform-d): δ [ppm] = 12.04 and 11.94 (s, 2H),[1] 8.49 and 8.43 (d, J = 9.2 Hz, 2H), 8.25 and 8.22 (d, J = 2.4 Hz, 2H), 6.45 – 6.42 (m, 2H), 3.18 (s, 12H), 2.62 and 2.56 (t, J = 7.2 Hz, 4H), 1.76 (quin, J = 7.2 Hz, 4H), 1.46 – 1.29 (m, 16H), 0.876 (t, J = 6.0 Hz, 6H). ^{13}C NMR (101 MHz, Chloroform-d): δ [ppm] = 182.74, 182.53 and 181.52 (2C), 175.86 and 175.33 (2C), 174.19 and 173.87 (2C), 156.88 (2C), 144.09 (2C), 133.95 and 133.30 (2C), 112.76 and 112.55 (2C), 108.64 (2C), 102.48 (2C), 40.63 (4C), 38.18 and 38.01 (2C), 31.93 and 31.89 (2C), 29.62, 29.40, 29.36 and 29.33 (4C), 25.42 and 25.42 (2C), 22.87 (2C), 14.53 (2C). MALDI/TOF found 602.42 m/z (calculated 602.38). FT-IR (cm^{-1}): 3135 (w), 3096 (w), 2953 (w), 2918 (m), 2872 (w), 2852 (m), 2808 (w), 2676 (w), 1706 (m), 1609 (s), 1578 (s), 1535 (m), 1483 (m), 1458 (m), 1389 (s), 1352 (s), 1278 (s), 1242 (s), 1207 (s), 1179 (s), 1154 (s), 1125 (s), 1090 (s), 1063 (s), 958 (m), 944 (m), 891 (s), 853 (s), 821 (s) 804 (m), 781 (s), 725 (m), 707 (m), 684 (m), 663 (m), 641 (m), 593 (w), 565 (w), 524 (s) 500 (s), 461 (s).

[1] See chapter "Temperature dependent NMR **a-SQ-1**" for explanation of the splitting

Synthesis of *S*-SQ-1

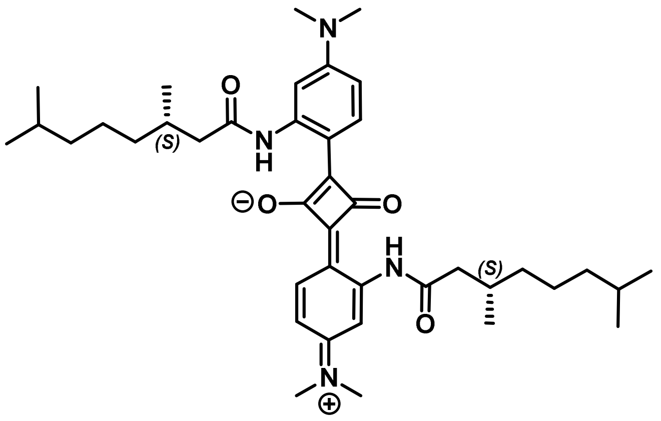

A flask filled with 100 mL of 50/50 *n*-propanol/toluene was fitted with a Dean-Stark trap and cooler and heated to 110 °C. After the trap was fully filled and manually emptied twice, squaric acid (99.7 mg, 0.86 mmol, 1 eq.) was added. After 30 minutes, (*S*)-*N*-*(*3-(dimethylamino)phenyl)-3,7-dimethyloctanamide (0.51 g, 1.7 mmol, 2 eq.) was added to the solution which turned dark green upon addition After 20 hours of reaction the mixture had a deep turquoise colour. The reaction mixture was cooled down and the solvent was removed by rotary evaporation to afford a green crude product (507 mg, 0.77 mmol, 90%). The crude product was recrystallised from acetonitrile to afford pure ***S*-SQ-1** in high yield (456 mg, 0.69 mmol, 81%). ^{1}H NMR (400 MHz, Chloroform-d): δ [ppm] = 12.08 and 11.94 (s, 2H), 8.55 and 8.49 (d, 9.2 Hz, 2H), 8.31 and 8.30 (d, 2.4 Hz, 2H), 6.48 (dd, J = 9.2 Hz, J = 2.4 Hz, 2H), 3.22 (s, 12H), 2.66 – 2.53 (m, 2H), 2.45 – 2.37 (m, 2H), 2.14 (br, 2H), 1.59 – 1.47 (m, 2H), 1.45 – 1.14 (m, 12H), 1.03 (d, J = 6.6 Hz, 6H), 0.88 – 0.84 (m, 12H). ^{13}C NMR (101 MHz, Chloroform-d): δ [ppm] = 182.74 and 181.68 (2C), 176.03 and 175.57 (2C), 173.85 and 173.47 (2C), 156.95 (2C), 144.09 and 143.98 (2C), 134.00 and 133.38 (2C), 112.80 and 112.59 (2C), 108.73 (2C), 102.60 (2C), 45.91 and 45.65 (2C), 40.67 (4C), 39.23, 37.37 and 37.23 (4C), 30.86 and 30.83 (2C), 28.15 (2C), 25.08 and 25.01 (2C), 22.87, 22.85, 22.75 and 22.71 (4C), 20.07 and 19.96 (2C). MALDI/TOF found 658.47 m/z (calculated 658.45). FT-IR (cm^{-1}): 3133 (w), 2953 (m), 2925 (m), 2868 (m), 2681 (w), 1710 (w), 1607 (s), 1574 (s), 1532 (m), 1484 (w), 1455 (m), 1412 (w), 1382 (m), 1354 (s), 1325 (s), 1277 (s), 1241 (s), 1195 (s), 1139 (s), 1124 (s), 1063 (s), 902 (s), 879 (s), 862 (s), 812 (s), 781 (s), 725 (s), 681 (m), 664 (m), 649 (m), 556 (w), 520 (s), 498 (s), 463 (m).

Synthesis of a-SQ-2

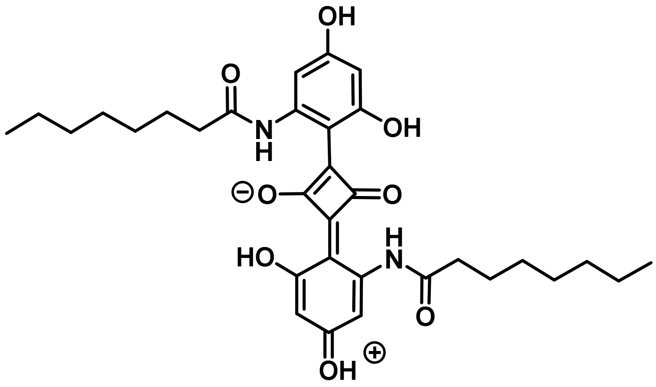

A flask filled with 50 mL of 50/50 *n*-propanol/toluene was fitted with a Dean-Stark trap and cooler and heated to 110 °C. Squaric acid (114 mg, 1.0 mmol, 1 eq.) was added and heating was continued for three hours. *N*-(3,5-Dihydroxyphenyl)octanamide (510 mg, 2.0 mmol, 2 eq.) was added. The mixture was heated and stirred for 20 h. The reaction mixture formed a deep blue colour, was cooled down and concentrated until dryness. The dark blue residue was dispersed in 30 mL ethyl acetate by sonication. After extraction with 20 mL 0.5 M aqueous hydrochloric acid solution a dark blue solid was filtered off the organic layer. The obtained product was dried in vacuum and characterised without further purification. **a-SQ-2** was obtained in 60% yield (350 mg, 0.6 mmol). ^{1}H NMR (400 MHz, DMSO-d$_6$): δ [ppm] = 12.46 (br, 2H), 11.26 (s, 2H), 7.58 (s, 2H), 6.00 (s, 2H), 2.40 (t, J = 7.4 Hz, 4H), 1.65 – 1.55 (m, 4H), 1.29 – 1.25 (m, 16H), 0.86 – 0.83 (m, 6H). MALDI/TOF found 580.30 m/z (calculated 580.28). FT-IR (cm^{-1}): 2954 (m), 2922 (m), 2854 (m), 2625 (m), 1681 (w), 1583 (s), 1516 (m), 1455 (m), 1427 (m), 1383 (m), 1322 (m), 1226 (m), 1191 (s), 1174 (s), 1135 (s), 1123 (s), 1099 (m), 1024 (s), 948 (m), 866 (m), 801 (s), 760 (s), 723 (s), 659 (m), 619 (m), 598 (m), 538 (m), 486 (s). Elemental analysis: found 66.10% C, 6.91% H, 4.81% N, 21.84% O (calculated: 66.19% C, 6.94% H, 4.82% N, 22.04% O).[2]

[2] The elemental analysis showed the presence of 0.34 wt% contamination of other elements than C, H, N and O. The material is most likely salt accumulated during the purification.

Synthesis of (3S,3'S)-N,N'-((2,4-dioxocyclobutane-1,3-diyl)bis(3,5-dihydroxy-2,1-phenylene))bis(3,7-dimethyloctanamide) *S*-SQ-2

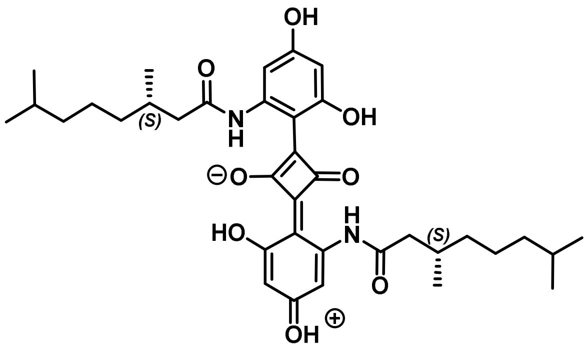

A flask filled with 50 mL of 50/50 *n*-propanol/toluene was fitted with a Dean-Stark trap and cooler and heated to 110 °C. Squaric acid (19 mg, 0.17 mmol, 1 eq.) was added and heating was continued for three hours. (*S*)-*N*-(3,5-dihydroxyphenyl)-3,7-dimethyloctanamide (100 mg, 0.36 mmol, 2.1 eq.) was added. The mixture was heated and stirred for 20 h. The reaction mixture formed a deep blue colour, was cooled down and concentrated until dryness. The dark blue residue was dispersed in 40 mL ethyl acetate by sonication. After extraction with 20 mL 0.5 M aqueous hydrochloric acid solution a dark blue solid was filtered off the organic layer. The obtained product was dried in vacuum and characterised without further purification. ***S*-SQ-2** was obtained in 11% yield (12 mg, 0.02 mmol). The low yield was caused by the small scale of the reaction. ^{1}H NMR (400 MHz, DMSO-d_6): δ [ppm] = 12.41 (br, 2H), 11.26 (s, 2H), 7.58 (s, 2H), 5.97 (s, 2H), 2.41 (dd, J = 14.2, 6.4 Hz, 2H), 2.23 (dd, J = 14.3, 7.6 Hz, 2H), 1.98 (br, 2H), 1.54 – 1.44 (m, 2H), 1.35 – 1.09 (m, 12H), 0.92 (d, J = 6.7 Hz, 6H), 0.83 (d, J = 6.6 Hz, 12H). MALDI/TOF found 659.34 m/z (calculated [M]+Na$^+$ 659.33). FT-IR (cm^{-1}): 2955 (m), 2928 (m), 2868 (m), 2620 (br), 1682 (w), 1610 (m), 1583 (s), 1517 (m), 1458 (w), 1426 (m), 1383 (m), 1325 (w), 1229 (s), 1196 (s), 1174 (s), 1125 (s), 1024 (s), 953 (m), 866 (m), 806 (m), 770 (m), 723 (s), 659 (w), 616 (m), 598 (m), 535 (m), 486 (m). Elemental analysis: found 66.91% C, 7.47% H, 4.29% N, 19.84% O (calculated: 67.90% C, 7.60% H, 4.40% N, 20.10% O).[3]

[3] The elemental analysis showed the presence of 1.49 wt% contamination of other elements than C, H, N and O. The material is most likely salt accumulated during the purification.

Huang-Rhys parameter *S* – calculation

The high molecular extinction coefficients of ***S*-SQ-1** and **a-SQ-1**, respectively, and the narrow absorption bandwidths are the result of the weak vibronic coupling for the electronic $S_0 \rightarrow S_1$ transition along the long molecular axis of the squaraine backbone.[1] Weak vibronic coupling is commonly caused by a low configurational displacement between initial and final state of an electronic transition and accompanied by weak manifestation of vibronic bands in the absorption spectrum. A quantitative theory on how strong vibrational progressions are observed in the absorption spectrum was developed by Huang and Rhys.[2] Although this theory was initially established to describe phonon-exciton coupling in ionic crystals, the Huang-Rhys parameter *S* is used in molecular spectroscopy, too and quantifies the intramolecular vibrational-electronic coupling.

Figure S1 shows the molar extinction coefficients ε of ***S*-SQ-1** and **a-SQ-1** in tetrahydrofuran as a function of the energy. The spectra of both compounds are very similar and characterised by a sharp maximum around 1.85 eV and the weak vibronic progression at 2 eV. The spectra were approximated using the software Origin 2019 and the Levenberg Marquardt algorithm. The chosen function consisted of two Gaussians to fit the $I_{0\text{-}0}$ and $I_{0\text{-}1}$ transitions.

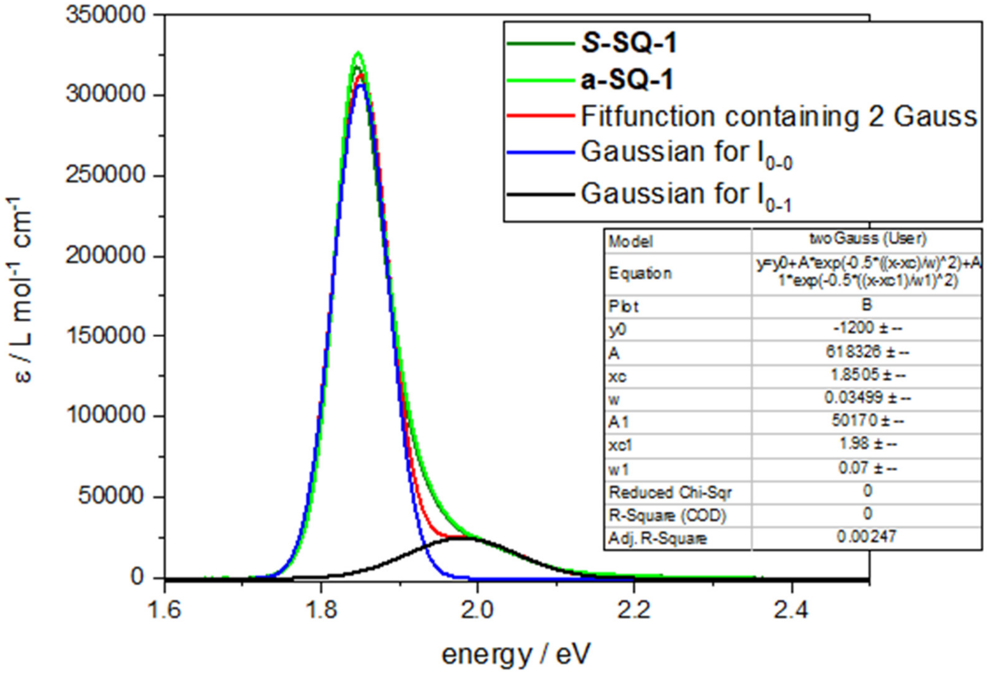

Figure S1: Molar extinction coefficients ε of ***S*-SQ-1** (dark green) and **a-SQ-1** (green) in tetrahydrofuran as a function of the wavelength. The concentrations are $2.6 \bullet 10^{-6}$ mol L^{-1}. The red line represents a fit-function for the spectral data. The function is based on two Gaussian functions as depicted in the in-set.

The fit-function was determined with

$$\varepsilon = -1200 + 618326 * e^{-0.5*\left(\frac{E-1.8505}{0.03499}\right)^2} + 50170 * e^{-0.5*\left(\frac{E-1.98}{0.07}\right)^2} .$$

Origin 2019 was used to plot and integrate this function. An area of 55642.9 was obtained.

By integrating $\varepsilon = -1200 + 50170 * e^{-0.5*\left(\frac{E-1.98}{0.07}\right)^2}$, the area under the baseline and the I_{0-1} transition was determined with 1263.2. Subtraction from the area of the complete fit function yielded the area under the I_{0-0} transition with 54379.7. Analogously the area under the I_{0-1} transition band was determined with 8803.

The Huang-Rhys parameter *S* is defined as

$$\frac{I_{0-0}}{I_{0-1}} = \frac{1}{S} \quad \text{(a)}$$

After transposing, the Huang-Rhys parameter *S* was determined with

$$S = \frac{I_{0-1}}{I_{0-0}} = \frac{8803}{55642.9} = 0.16$$

The energy difference between both absorption bands was determined with 0.13 eV or 1040 cm^{-1}. The corresponding vibration of the conjugated backbone is suggested to be IR silent owing to its symmetrical character. The FT-IR spectra of ***S*-SQ-1** and **a-SQ-1** depicted in Figure S38 and S39 match this expectation and do not show a distinguished band at this wavenumber.

Photophysical properties – steady state

We measured steady-state absorption and emission spectra of all dyes in a range of solvents that differ in polarity. The spectra recorded in acetonitrile, chloroform, heptane, methanol, tetrahydrofuran and water are presented in Figures S2-5. Depending on the chosen solvent, three different absorption regimes were found for the dyes. For all dyes, narrow absorption bands around 600 to 700 nm were found in most organic solvents. For ***S*-SQ-2** and **a-SQ-2** a second absorption band was found around 500 nm in several organic solvents. All dyes showed the presence of very broad absorption bands in water. After comparison with literature reports,[3–5] narrow absorption bands around 670 nm (found for ***S*-SQ-1** and **a-SQ-1**) or 605 nm (found for ***S*-SQ-2** and **a-SQ-2**), respectively, were assigned to the molecularly dissolved state. ***S*-SQ-2** and **a-SQ-2** exhibited shorter wavelengths of maximum absorbance and lower molar absorptivity than ***S*-SQ-1** and **a-SQ-1**. This observation was explained by the less pronounced intramolecular charge transfer between the quadratic squaraine core and bis-hydroxy-arene substituents with respect to the *N*,*N*-dialkylaminoaryl moieties.

For ***S*-SQ-2** and **a-SQ-2**, additional blue-shifted absorption bands were recorded in acetonitrile, methanol and *N,N*-dimethylformamide. The bands were ascribed to the presence of a deprotonated species. A detailed study on the dependency of the photophysical properties on the presence of acid or base is described in the section “Photophysical properties – stimuli responsiveness”.

Broad absorption bands in the near-infrared regime were noticed for all the presented dyes in the bad solvent water and assigned to the formation of aggregates. Spectral properties of aggregated ***S*-SQ-1** and **a-SQ-1** are given in the main article. See the section “Blue dye: supporting information on aggregation” for further information on the aggregation behaviour of ***S*-SQ-2** and **a-SQ-2**.

***S*-SQ-1** absorption and emission in steady state:

The wavelength of maximum absorbance $\lambda_{max\ OD}$ was determined for ***S*-SQ-1** in several solvents (Spectra shown in Figure S2). When $\lambda_{max\ OD}$ was plotted as a function of the solvents polarity expressed as the $E_T(30)$ value, no linear trend was observed. Solvatochromism (e.g. negative solvatochromism: blue shift with increasing solvent polarity caused by the ground state being better solubilised than the excited state[6]) is hence not observed. The absence of solvatochromism indicates the formation of hydrogen-bonds with solvents that are capable of forming these. Fluorescence is observed in all solvents and the observed Stokes shifts hardly depend on polarity.

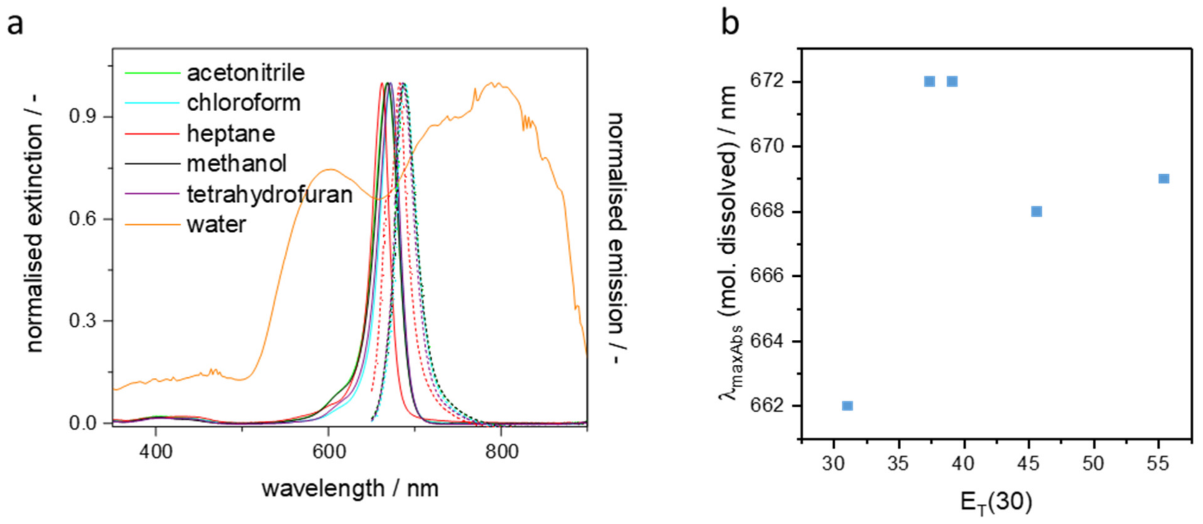

Figure S2: (a) Absorption (solid lines) and fluorescence spectra (dashed lines) recorded for ***S*-SQ-1** in various solvents. Concentrations: 2.6 e-5 M in organic solvents and 1.3 e-5 M in water. No fluorescence was detected in water. (b) Plotting the maximum absorption wavelength against the used solvent's solvent polarity expressed by the $E_T(30)$ value does not result in a linear trend indicating ***S*-SQ-1**'s capability for forming hydrogen bonds with solvent molecules.

solvent	$E_T(30)$ /kcal mol^{-1}[6]	λmaxAbs	λmaxEm	Stokes shift
Acetonitrile	45.6	668	688	20
Chloroform	39.1	672	689	17
Heptane	31.1	662	683	21
methanol	55.4	669	687	18
tetrahydrofuran	37.4	672	686	14

a-SQ-1 absorption and emission:

The recorded absorption (solid lines) and emission properties for **a-SQ-1** are similar to the spectra recorded for its chiral counterpart except for the bad solvent water where **a-SQ-1** forms H-type aggregates instead of a broad aggregate.

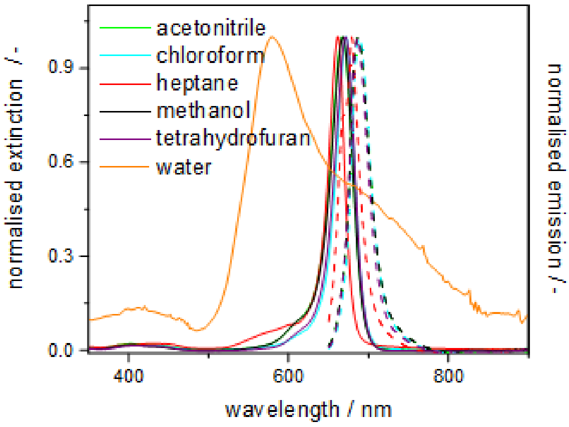

Figure S3: Absorption (solid lines) and fluorescence spectra (dashed lines) recorded for **a-SQ-1** in various solvents. Concentrations: 2.6 e-5 M in organic solvents and 1.3 e-5 M in water. No fluorescence was detected in water.

solvent	$\lambda_{max,Abs}$ [nm]	$\lambda_{max,Em}$ [nm]	Stokes shift [nm]
Acetonitrile	667	687	20
Chloroform	672	689	17
Heptane	662	679	17
methanol	668	687	19
tetrahydrofuran	671	686	15

__*S*-SQ-2 absorption:__

Absorption and emission were measured for ***S*-SQ-2** in several solvents. The investigation of the dependency of $\lambda_{max\ OD}$ on the solvent's polarity is hampered since the spectra recorded in the solvents methanol or acetonitrile revealed the presence of the singly deprotonated species only. In contrast to ***S*-SQ-1, *S*-SQ-2** forms aggregates both in water and heptane. Fluorescence was detected for none of the samples.

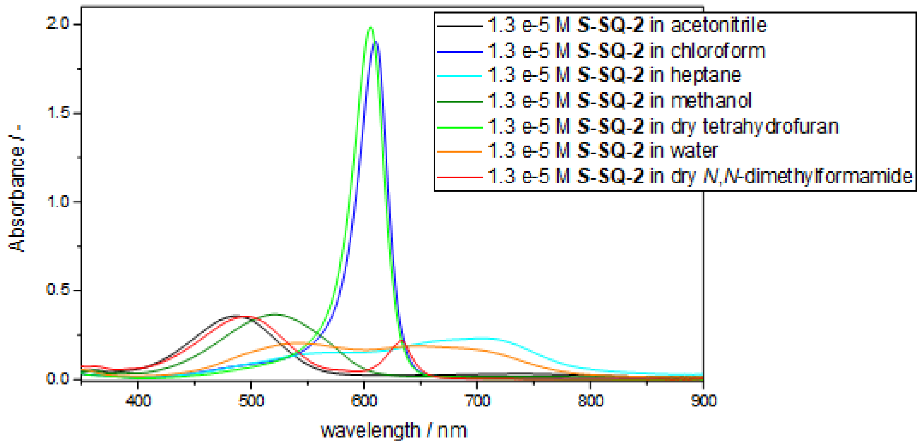

Figure S4: Absorption spectra recorded for ***S*-SQ-2** in various solvents.

__a-SQ-2 absorption:__

Analogously to ***S*-SQ-2**, recorded spectra for **a-SQ-2** were governed by deprotonation and aggregation of the dye.

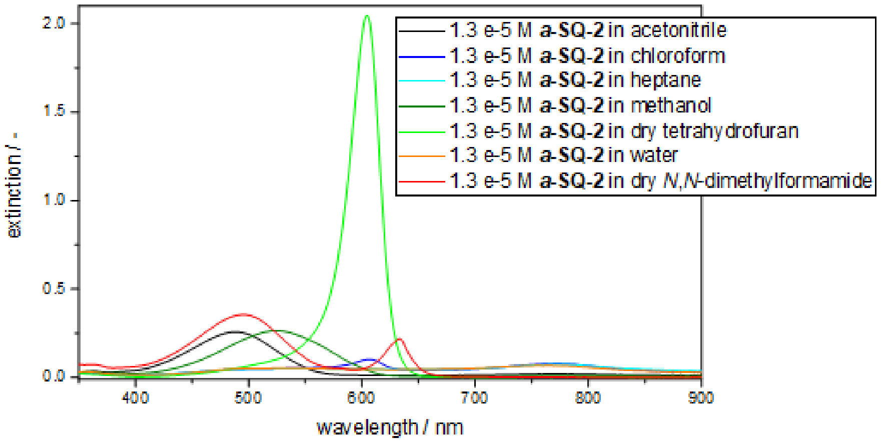

Figure S5: Absorption spectra recorded for **a-SQ-1** in various solvents.

Photophysical properties changed by addition of acid or base

***S*-SQ-1** and **a-SQ-1** contain amine and ***S*-SQ-2** and **a-SQ-2** hydroxyl groups. Since these groups are also part of the aromatic system, dis- and association reactions can be used to tune the absorption properties of the dyes.

Upon adding trifluoroacetic acid to a solution of molecularly dissolved ***S*-SQ-1** or **a-SQ-1** in $CHCl_3$, the dyes' amine groups were protonated and the solution turned its colour from green to violet. A rise of blue-shifted bands was observed in the respective absorption spectrum (Figure S6a).

In case of ***S*-SQ-2** or **a-SQ-2**, the presence of a blue-shifted absorption band was noticed when the spectrum was recorded in PA grade tetrahydrofuran instead of dry solvent (see dark blue line in Figure S7). The addition of triethylamine to the solution resulted in complete depletion of the absorption band of the molecularly dissolved neutral species. The reaction changed the colour of the solution of ***S*-SQ-2** or **a-SQ-2** from blue to red (Figure S6b). Subsequent acidification restored the blue colour and the absorption band of the molecularly dissolved species (see light blue line in Figure S7). The spectra obtained for several cycles of (de-)protonating **a-SQ-2** are depicted in Figure S8 and prove the reversibility of the reaction.

For all dyes, the generation of the singly charged form resulted in a hypsochromic (blue) shift in the absorbance spectra and broadening of the bandwidth. The observation was explained by resonance theory. The dyes are electronically symmetrical in their neutral form. The geometries of the molecules in both ground and first excited state are very similar. Once a single charge has to be distributed on a protonated form of ***S*-SQ-1** or **a-SQ-1**, or a deprotonated ***S*-SQ-2** or **a-SQ-2** molecule, the structures become electronically unsymmetrical. As a result, the observed hypsochromic shift and band broadening emerge in the absorption spectra. While the protonation of **a-SQ-1** did only occur as a distinct equilibrium reaction at even higher TFA concentrations ($c(TFA) > 0.1$ M, see Figure S6a), **a-SQ-2** was readily deprotonated by small amounts of triethylamine. The observation is explained by resonance theory, too. As depicted in Scheme S2, the CT interaction between the electron poor squaraine core and electron rich moieties results in formally charged groups in the donor part. The basicity of the amine group in **a-SQ-1** is hence reduced and its reaction with available acids is less pronounced. In case of **a-SQ-2**, the same effect increases the acidity of the hydroxyl group in para position to the squaric core and makes **a-SQ-2** react with traces of water being present in PA grade tetrahydrofuran. Singly deprotonated **a-SQ-2** can be deprotonated further by the addition of KOH in ethanol solution (as shown in Figure S6b). As the neutral form, doubly deprotonated **a-SQ-2** is electronically symmetrical. The wavelength of maximum absorption is red-shifted. In contrast to neutral and mono-anion, the dianion shows fluorescence with a Stokes shift

of 27 nm. The observation matches literature reports on a phloroglucinol based squaraine dye that exhibits fluorescence solely after double deprotonation.[5]

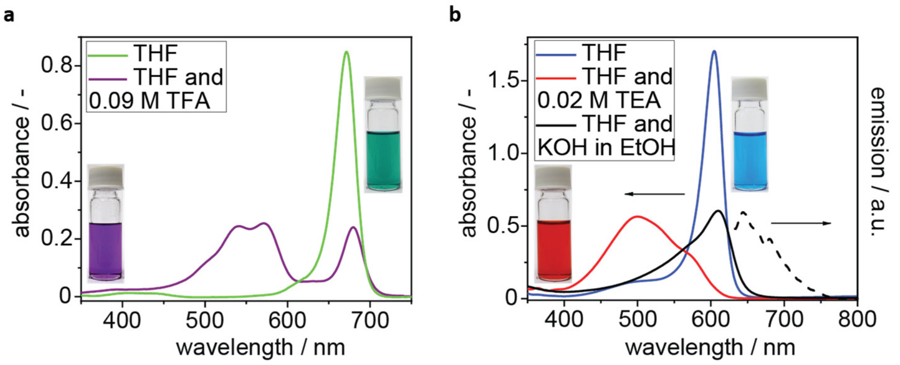

Figure S6: Photophysical properties of molecularly dissolved solutions of **a-SQ-1** or **a-SQ-2**, respectively, recorded dry THF. (a) The amine group of **SQ-1** is protonated by the addition of trifluoroacetic acid (0.09 M). (b) Addition of TEA results in single deprotonation of **a-SQ-2**. The second hydroxyl group is deprotonated by the addition of a saturated solution of KOH in ethanol. In contrast to neutral and singly dissociated form, doubly deprotonated **a-SQ-2** exhibits fluorescence. Concentrations: **a-SQ-1**: $2.6{\cdot}10^{-6}$ M, **a-SQ-2**: $1.3{\cdot}10^{-5}$ M. The insets show the colour impression of more concentrated samples. Violet: **a-SQ-1** and TFA, green: **a-SQ-1**, red: **a-SQ-2** and TEA, blue: **a-SQ-2** as well as **a-SQ-2** after the addition of KOH in ethanol.

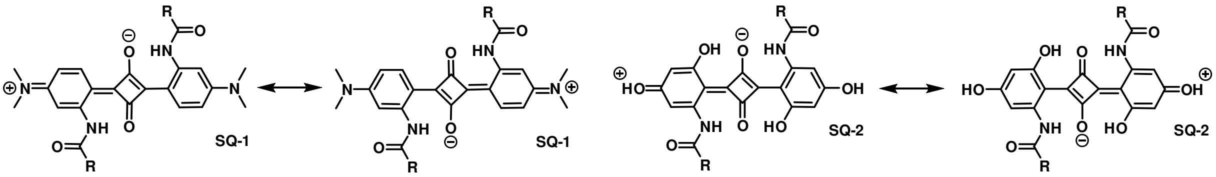

Scheme S2: Mesomerism of the squaraine dyes.

<u>***S*-SQ-2** in tetrahydrofuran (PA) grade: anion band visible</u>

The amount of water which is present in PA grade tetrahydrofuran is sufficient to deprotonate ***S*-SQ-2** to a large extend as clearly visible in the formation of a broad absorption band around 500 nm (dark blue line in Figure S7). Full deprotonation is achieved by the addition of a droplet of triethylamine. The observed blue-shift can be reversed by the addition of a droplet of aqueous solution of hydrochloric acid (3 M). Anionic ***S*-SQ-2** is protonated again.

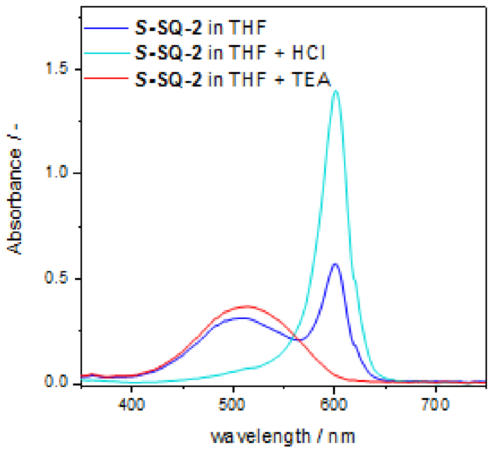

Figure S7: Absorption spectroscopy reveals two absorption bands for a solution of $1.0 \cdot 10^{-5}$ mol/l of ***S*-SQ-2** in PA grade tetrahydrofuran (THF). When the solution is acidified by addition of hydrochloric acid, the broad absorption band with a maximum at 510 nm is depleted and the only the absorption band of the molecularly dissolved species with a maximum at 602 nm is noticed. After addition of triethylamine, the absorption band of the molecularly dissolved species is depleted and an increase of the intensity of the broad absorption band is noticed.

a-SQ-2 in tetrahydrofuran (PA) grade: anion band visible, double deprotonation by addition of KOH in ethanol

The reversibility of the deprotonation reaction is depicted in Figure S8. The recorded spectra after the addition of triethylamine or trifluoroacetic acid of two cycles match in absorbance. Only after addition of KOH in ethanol and subsequent protonation by TFA, a decreased absorbance is noticed for the absorption band of the neutral species. The decrease is caused by a dilution effect when adding KOH in ethanol to achieve double deprotonation.

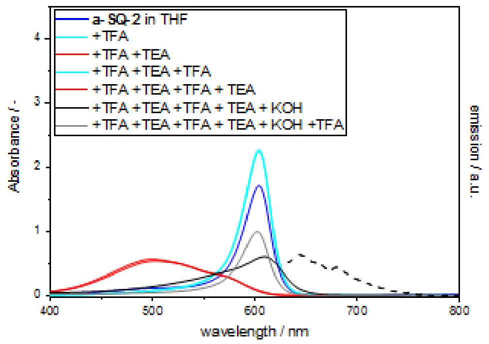

Figure S8: Several cycles of alternating acid and base induced switching between neutral and charged forms of **a-SQ-2** in a $1.3 \cdot 10^{-5}$ mol/l solution in tetrahydrofuran were followed by UV Vis absorption spectroscopy.

Blue dyes *S*-SQ-2 and a-SQ-2: chemical stability:

During the measurements of the absorption spectra of ***S*-SQ-2** and **a-SQ-2**, we noticed that the blue colour of the stock solutions was fading when stored over several weeks at room temperature. In order to probe the chemical stability of bis-hydroxy-arene substituted squaraine dyes, we performed NMR experiments of solutions of ***S*-SQ-2** in DMSO. Whereas a spectrum recorded of a freshly prepared solution showed the correct ratio of the signals for ***S*-SQ-2**'s protons, decomposition was followed by NMR spectroscopy (Figure S9). The decomposition could be sped up by sonication of the molecularly dissolved sample. After sonicating a sample for 30 minutes, the ratio of the alkyl to the aryl protons was already doubled and indicated decomposition. The observation stressed that in contrast to amino-substituted diaryl derivatives, bis-hydroxy-arene substituted squaraine dyes are chemically sensitive compounds whose solutions must be handled with care.

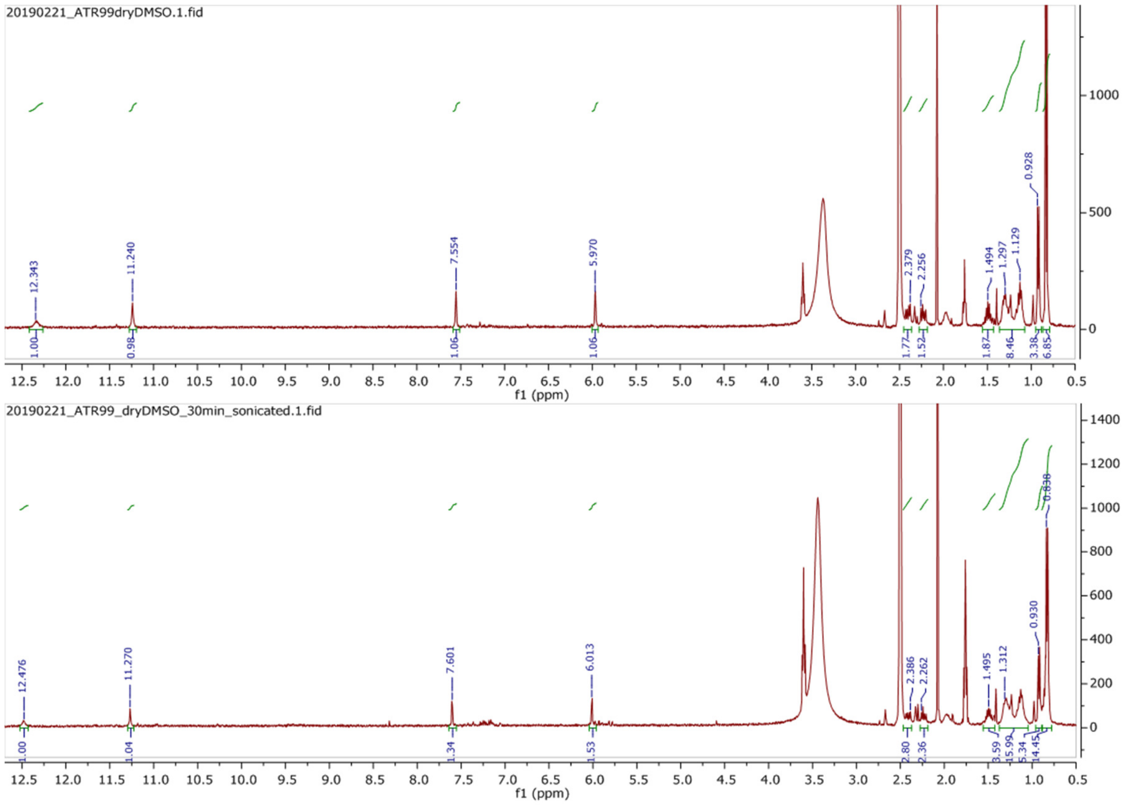

Figure S9: ^{1}H NMR spectroscopy of ***S*-SQ-2** in DMSO-d$_6$. The spectrum of the as prepared sample shows the correct ratio of alkyl to aryl protons. After sonication for 30 min, the spectrum shows a relative increase of the signals of the alkyl protons with respect to the signals of the aryl protons by factor 2.

Green dyes *S*-SQ-1 and a-SQ-1: supporting information on aggregation

Impact of film thickness on optical properties

Films with thicknesses ranging from 11 to 81 nm (thickness measured by surface profilometer) were prepared by spin coating (concentrations 5, 10 and 20 mM, rotation speed 1000 and 2000 rpm). The UV-Vis and circular dichroism spectra recorded for the films are depicted in Figure S10. Subsequently, we divided the ellipticity recorded wavelength of 790 and 495 nm by the film thickness in order to see whether the ellipticity over thickness classifies as an intensive property of the material. The data is depicted in Figure S10c. It is apparent that increasing the film thickness resulted in an increase of the ellipticity/thickness ratio when comparing the rather thin films (11-24 nm) with thicker ones (43-81 nm). For a purely excitonic origin of the CD effect, no thickness dependency is expected. The increase of the CD effect of the material with increasing thickness suggests that other effects such as cholesteric or a combination of linear dichroism with linear birefringence are active in the thicker films.

We probed the presence of linear dichroism in the films (Figure S10d). The highest linear dichroism is displayed by the thickest film, amounting to a value of 0.002 when expressed as differential absorbance for the 81 nm thick film. This linear dichroism can, in combination with linear birefringence in either the optical setup or the sample itself, lead to an artificial contribution to the circular dichroism.

In order evaluate this possibility, we first convert the measured ellipticity to circular differential absorbance $\Delta A = A_L - A_R$. For the 81 nm thick sample the maximum ellipticity of 342 mdeg correspond with a differential absorbance ΔA of 0.01. The circular differential absorbance is five times higher than the linear differential. We conclude that an artificial contribution to the circular dichroism must be small especially considering the fact the linear birefringence is of limited magnitude, typically below 0.1.

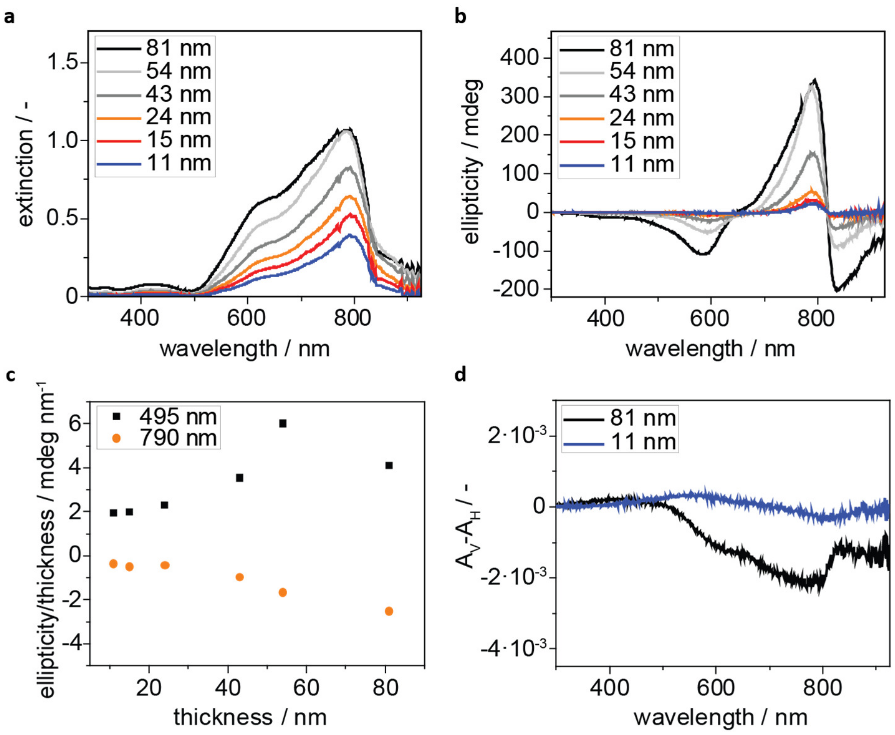

Figure S10: Increasing absorption (a) and CD effect (b) when increasing the thickness of a coassembled film containing 25% **S-SQ-1** and 75% **a-SQ-1**. For the 81 nm thick sample, dewetting started to occur. (c): Variation of the dissymmetry factor g_{abs} with increasing film thickness. (d): linear dichroism expressed as the difference in absorbance for vertically and horizontally polarized light A_V-A_H for the 11 nm and 81 nm thick samples.

<u>Linear and circular dichroism spectra for films of ***S*-SQ-1** and **a-SQ-1** before and after thermal annealing:</u>

Spin coated thin films of ***S*-SQ-1** and **a-SQ-1** where found to display little linear dichroism effect (Figure S11). After annealing at 210 °C for 10 min under argon atmosphere, linear dichroism spectra were recorded again. Only for the sample containing an equimolar mixture of ***S*-SQ-1** and **a-SQ-1**, a minor increase in linear dichroism is noticed which suggests slight a growth of the ordered domains.

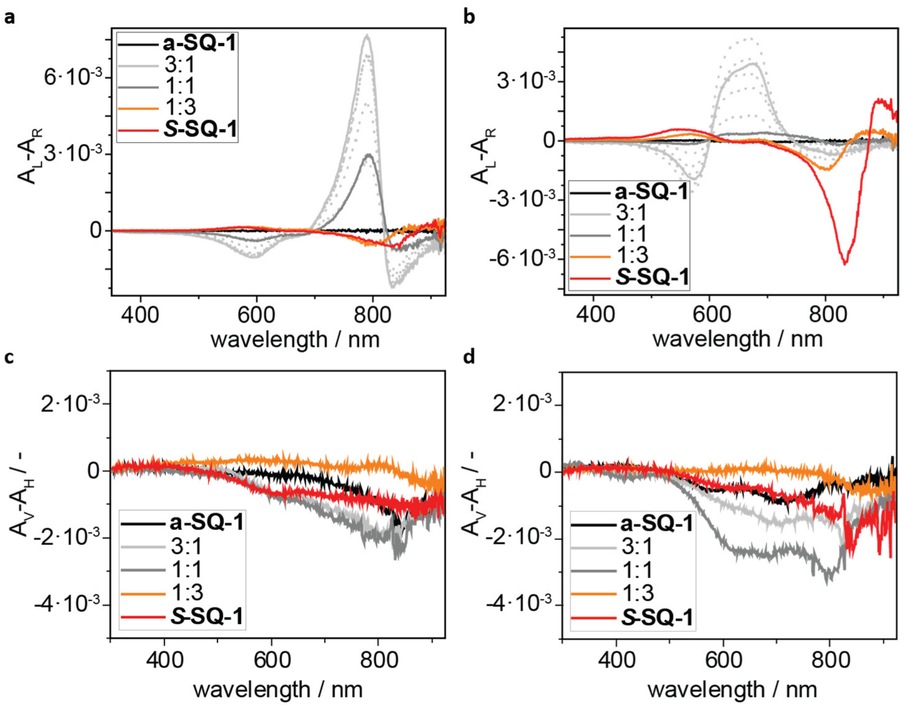

Figure S11: Circular and linear dichroism spectra for spin coated thin films with increasing content of ***S*-SQ-1** in a mix of ***S*-SQ-1** and **a-SQ-1**. Dichroism is expressed as differential absorbance. Graphs (a,c) show results for the films as cast, while (b,d) pertain to the films after thermal annealing. The increase in linear dichroism upon thermal annealing (d) hints at growth of ordered domains.

Estimation of the dissymmetry factor g_{abs}:

We spin coated thin films (speed 1000 rpm) from 10 mM chloroform solutions containing ***S*-SQ-1** and **a-SQ-1** in mixing ratios of 0/1, 3/1, 1/1, 1/3 and 1/0. We measured ellipticity, transmission (T) and reflection (R) spectra of all thin films. The reflectance was measure with incident beam under near normal incidence (15°). The reflection and transmission for s- and p-polarized light were measured separately. The spectra are depicted in Figure S12.

The films show high reflectivity in the wavelength range just below the onset of absorption, amounting to 40-50%. In order to get an estimate for the absorbance by the film, we correct the transmission for reflection loose and approximate

$$A = -\,{}^{10}\log(T+R)$$

Finally, we take the arithmetic average of the absorbance for s and p polarized light. We combine the ellipticity and the corrected, averaged absorbance to evaluate the dissymmetry factor g_{abs} defined as

$$g_{abs} = \frac{A_l - A_R}{A}$$

where A is the absorbance measured using unpolarised light and $A_{L(R)}$ the absorbance for left (right) circularly polarized light. The circular differential corresponds to the value for the ellipticity in mdeg divided by 32982. The estimates for g_{abs} as obtained for all mixtures are shown in Figure S13a. The highest g_{abs} of 0.02 is found for a 1/3 mixture of ***S*-SQ-1** and **a-SQ-1**. The variation of g_{abs} with increasing amount of ***S*-SQ-1** in the feed is shown in Figure S13b.

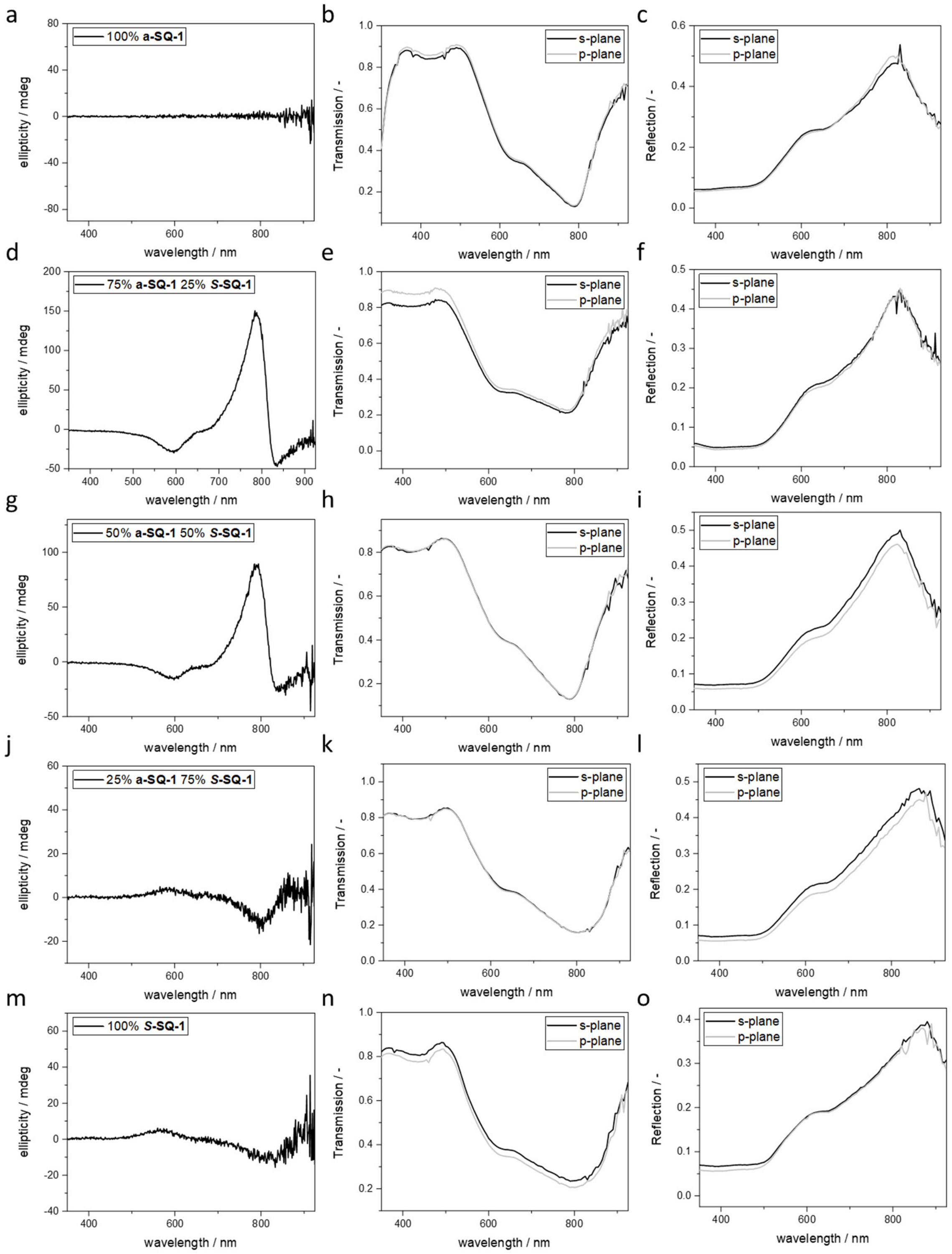

Figure S 12: Ellipticity, transmission and reflection spectra recorded for ***S*-SQ-1** and **a-SQ-1** in mixing ratios of 0/1 (a-c), 3/1 (d-f), 1/1 (g-i), 1/3 (j-l) and 1/0 (m-o).

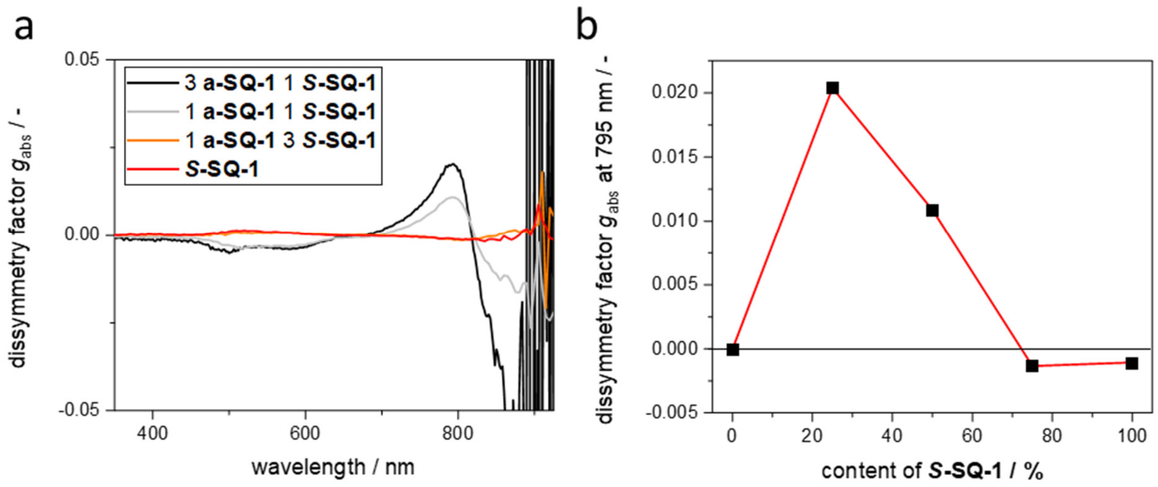

Figure S 13: Dissymmetry factor g_{abs} with increasing content of ***S*-SQ-1** in a mixture of ***S*-SQ-1** and **a-SQ-1**. (a) g_{abs} as a function of the wavelength for all mixtures as derived from CD, absorption and reflection spectroscopy; (b) Variation of g_{abs} with increasing amount of ***S*-SQ-1**.

Müller matrix spectroscopy

The stokes vector I describing the intensity and polarization of light may be defined as

$$I = \begin{bmatrix} I_1 \\ I_2 \\ I_3 \\ I_4 \end{bmatrix} = \begin{bmatrix} I_X + I_Y \\ I_Y - I_X \\ I_M - I_P \\ I_R - I_L \end{bmatrix}$$

Where R(L) denote the circular polarization of light while *X*,*Y*,*P*, and *M* refer to the linear polarization of light defined according to :

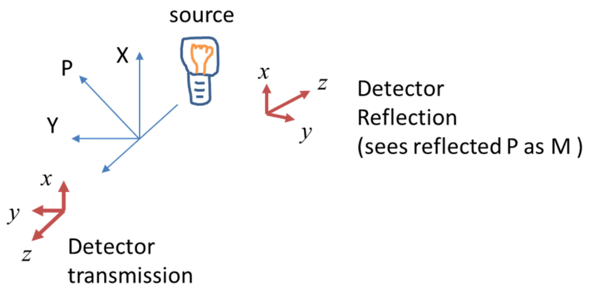

In order to describe the stokes vector of the light after having passed through or being reflected by the film we use the following Müller matrix :

$$I_{out} = \begin{bmatrix} I_{1,out} \\ I_{2,out} \\ I_{3,out} \\ I_{4,out} \end{bmatrix} = \begin{bmatrix} M_{11} & M_{12} & M_{13} & M_{14} \\ M_{21} & M_{22} & M_{23} & M_{24} \\ M_{31} & M_{32} & M_{33} & M_{34} \\ M_{41} & M_{42} & M_{43} & M_{44} \end{bmatrix} \begin{bmatrix} I_{1,in} \\ I_{2,in} \\ I_{3,in} \\ I_{4,in} \end{bmatrix}$$

Using a W-VASE spectroscopic ellipsometer from J.A. Woollam, we have determined values for most of the elements of the Müller matric normalized M_{11} , e.g.

mm12 = M_{12}/M_{11}

In the graphs below we show the results for transmission under normal incidence for five films with different composition in terms of ***S*-SQ-1** and **a-SQ-1.** For the film with ***S*-SQ-1** and **a-SQ-1** in 1:3 ratio we also show the Muller matrix for reflection of light under near-normal incidence reflection.

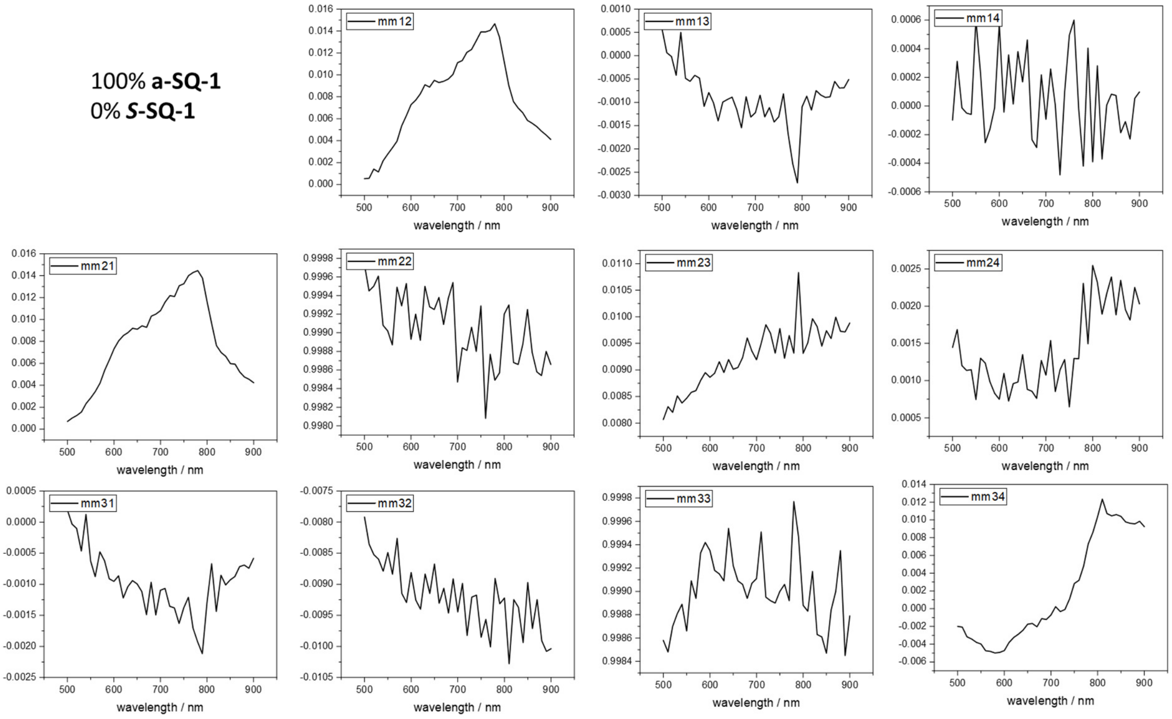

Figure S 14: Muller matrix spectroscopy for **a-SQ-1.** As expected, this film shows essentially zero circular differential transmission of light (mm14) but a relatively high degree of linear dichroism (mm12).

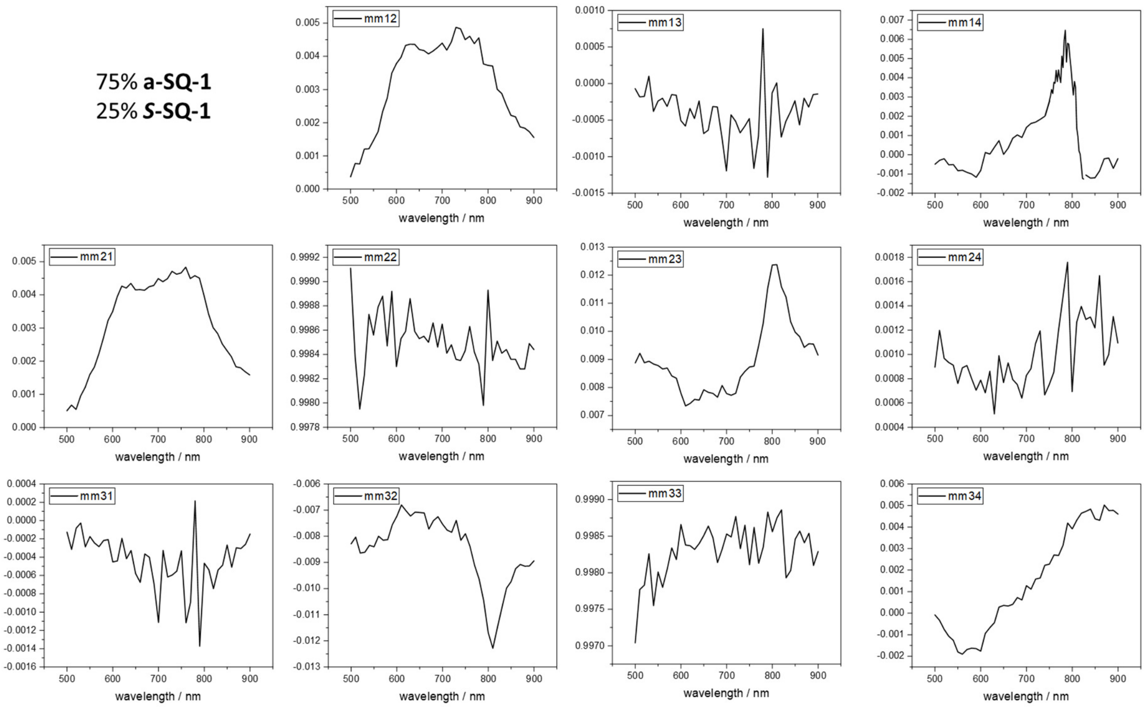

Figure S 15: Muller matrix spectroscopy for a 1/3 mixture of ***S*-SQ-1** and **a-SQ-1.** For this film we find the highest relative circular differential transmission (mm14) of all the five films studied. Also, relatively high values for the elements mm23 and mm32, related to the rotation of the plane of polarization upon transmission though the film (optical rotation) are noticeable.

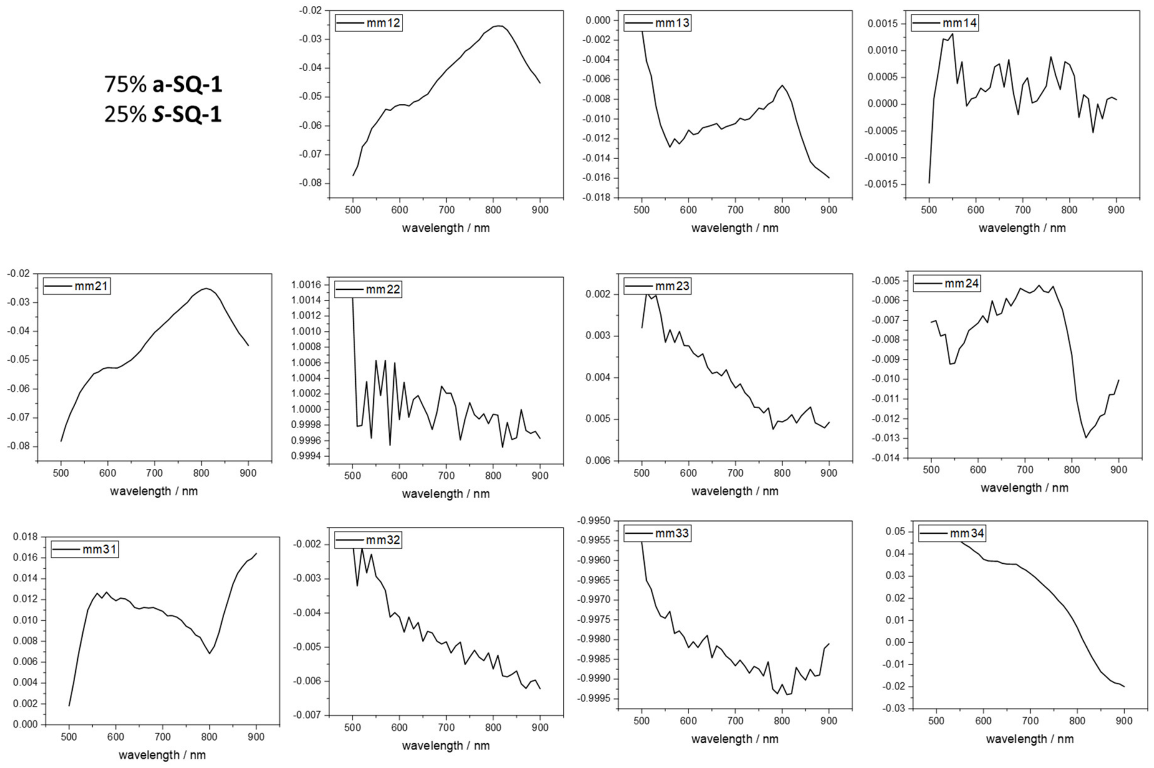

Figure S 16: Muller matrix spectroscopy for a 1/3 mixture of ***S*-SQ-1** and **a-SQ-1** in reflection under near normal incidence. Values for circular differential reflection of light (mm14) are small and cannot be distinguished with certainty from zero.

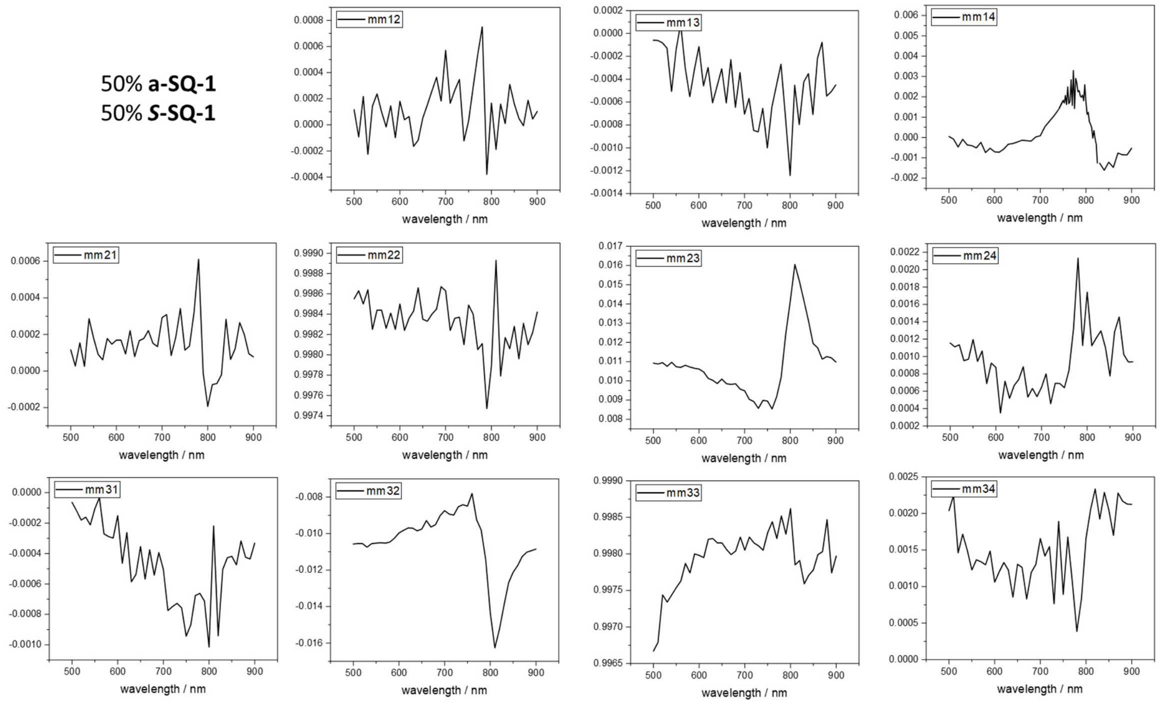

Figure S 16: Muller matrix spectroscopy for 1/1 mixture of ***S*-SQ-1** and **a-SQ-1**

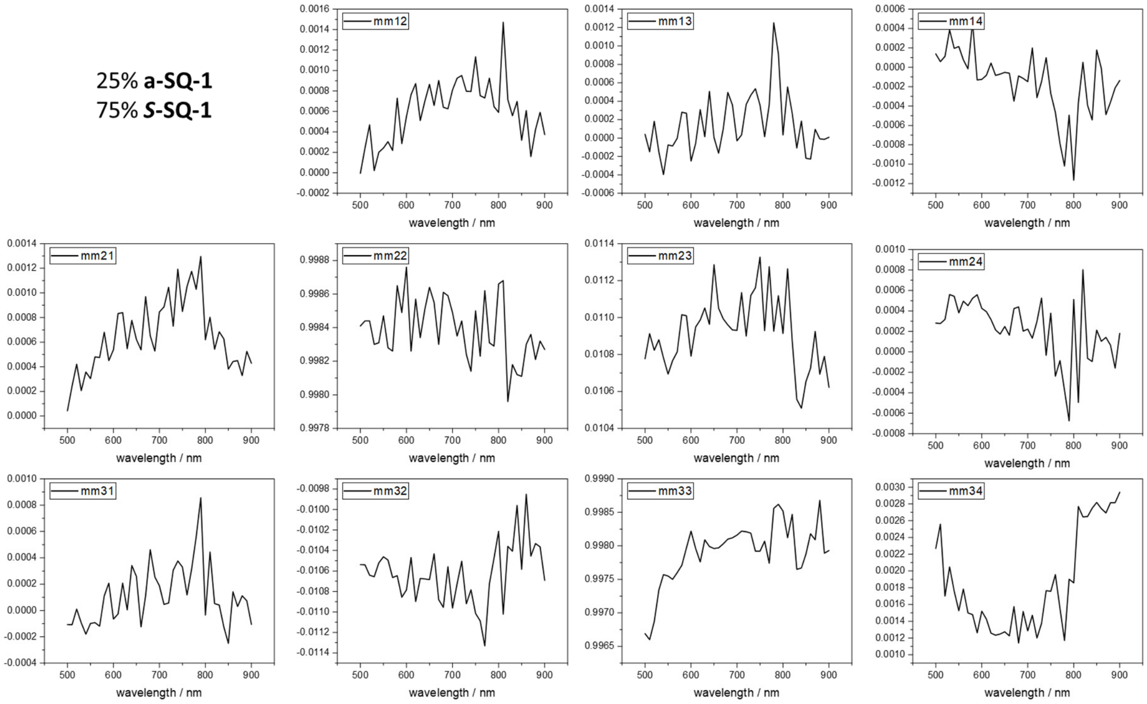

Figure S 17: Muller matrix spectroscopy for 3/1 mixture of ***S*-SQ-1** and **a-SQ-1**

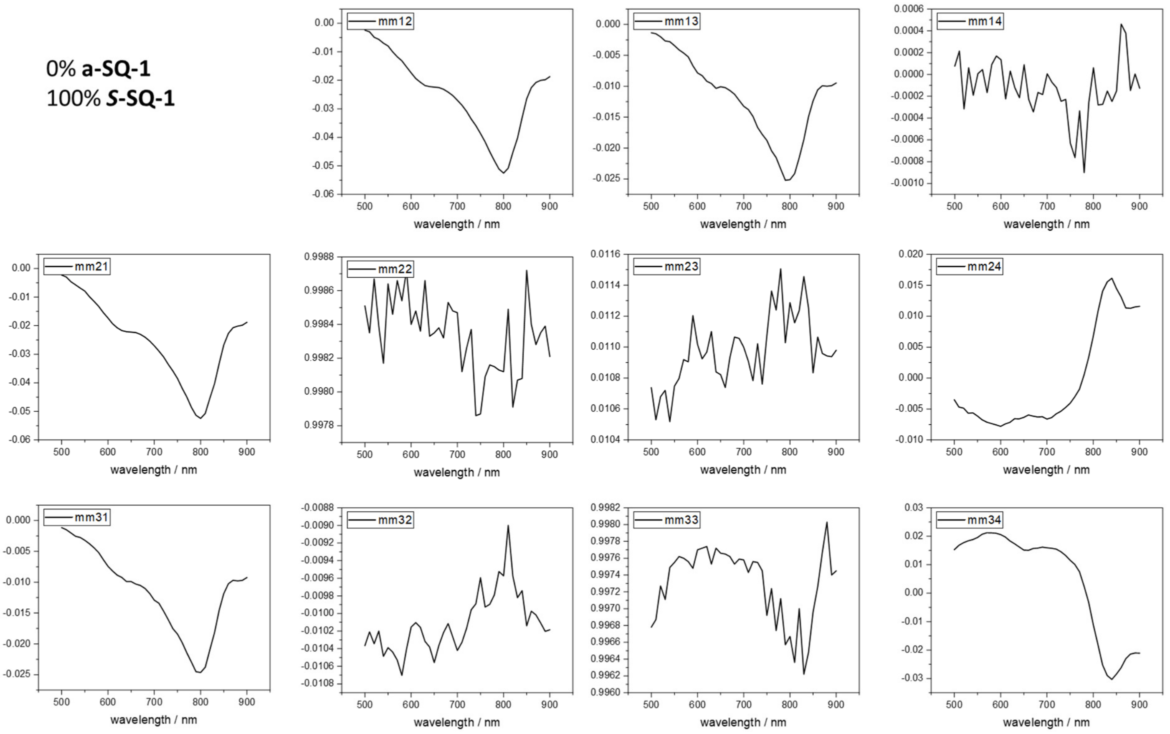

Figure S 18: Muller matrix spectroscopy for mixture of ***S*-SQ-1.**

Green dyes *S*-SQ-1 and a-SQ-1: POM images of spin coated thin films

The spin coated thins films were investigated by polarised optical microscopy (POM). All films show a green-bluish colour when the polarisers are not crossed. After crossing the polarisers, light was no longer transmitted. The black images demonstrate the absence of birefringent texture. Exemplary images for **a-SQ-1**, ***S*-SQ-1** and a 3:1 mixture thereof are shown in Figure S 19.

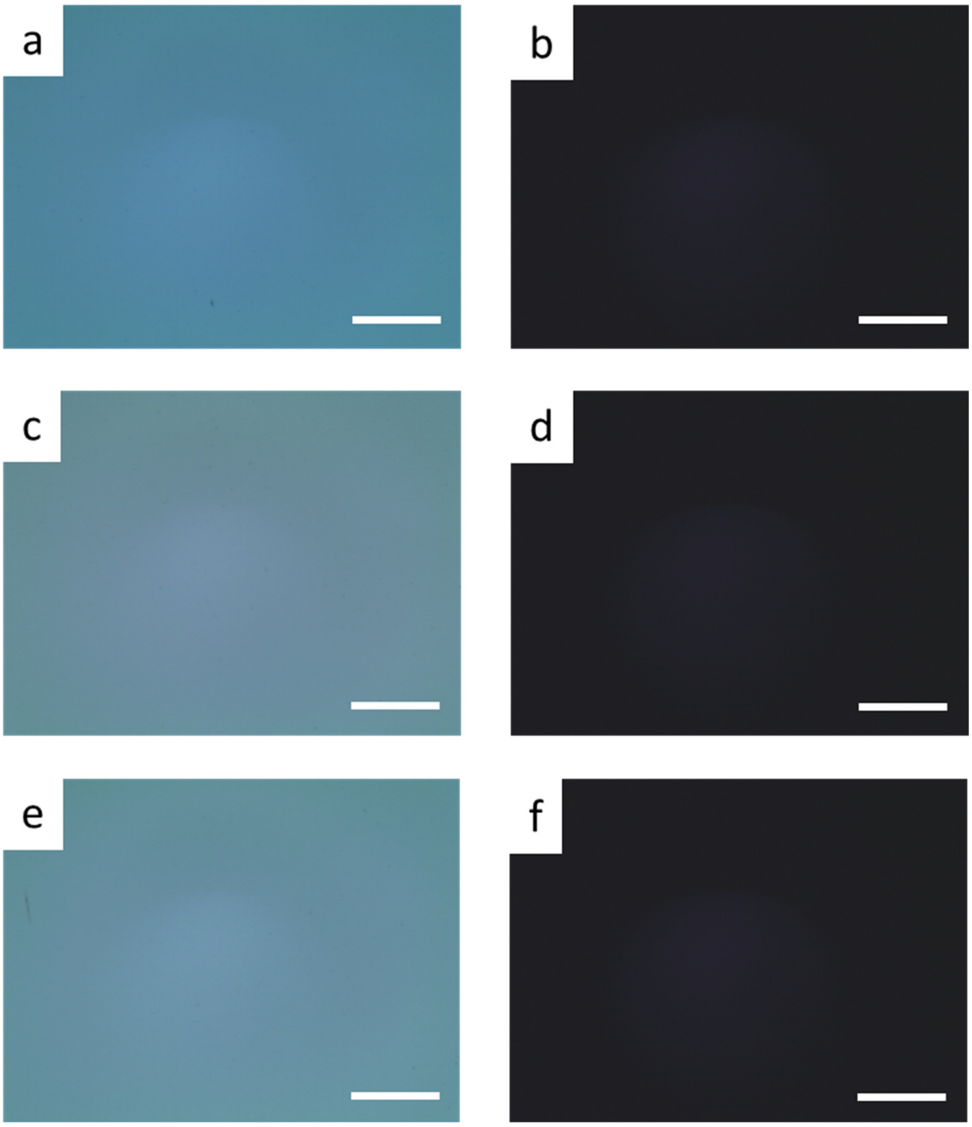

Figure S 19: POM images taken for **a-SQ-1** (a) and (b), ***S*-SQ-1** (c) and (d)and a 3:1 mixture thereof (e) and (f). Parallel oriented polarisers show the green-bluish color of all films (a, c, e). After crossing the polarisers, no light is transmitted and the obtained images are black (b, d, f).

Green dyes *S*-SQ-1 and a-SQ-1: AFM images of spin coated thin films

We investigated the thin films prepared by spin coating by atomic force microscopy (AFM). The surface morphologies of the samples containing pure **a-SQ-1**, ***S*-SQ-1** and the 3:1 mixture thereof are shown in Figure S 20. All films show smooth surfaces.

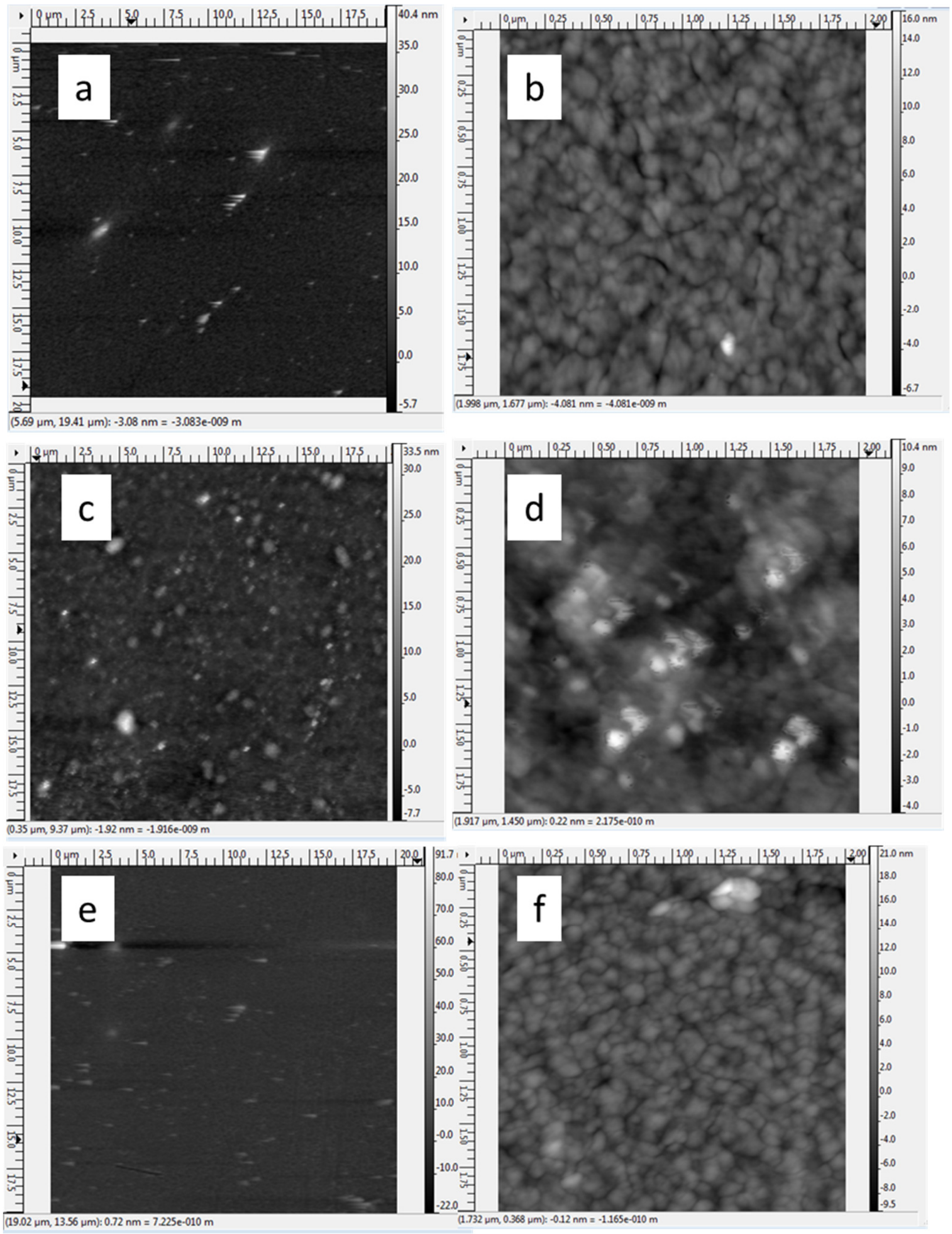

Figure S 20: AFM micrographs showing the height profiles of the thin films formed by **a-SQ-1** (a) and (b), ***S*-SQ-1** (c) and (d)and a 3:1 mixture thereof (e) and (f). The images show 20 µm x20 µm (a, c, e) and 2 µm x 2 µm (b, d, f) sections.

Blue dyes S-SQ-2 and a-SQ-2: supporting information on aggregation

Aggregation of ***S*-SQ-2** and **a-SQ-2** in solution and on surface

Aggregation could be induced for hydroxy aryl substituted squaraine dyes ***S*-SQ-2** and **a-SQ-2**, too. As shown in the steady state absorption spectra (Figure S4 and S5), bands assigned to aggregated ***S*-SQ-2** and **a-SQ-2** were recorded in the solvents heptane, water and toluene. Since **a-SQ-2** has a somewhat lower solubility than ***S*-SQ-2**, **a-SQ-2** could be aggregated in chloroform, too. A more detailed investigation of the aggregation behaviour of ***S*-SQ-2** in toluene will be presented in the following.

After addition of a concentrated aliquot of ***S*-SQ-2** to toluene, an increase of a broad band was noticed in the absorption spectrum (violet dashed line in Figure S13a). The optical transitions were CD active in the visible and near infra-red regime (violet line in Figure S13a). Heating and annealing the solution at 95 °C resulted in the depletion of these broad absorption bands and only molecularly dissolved species was noticed. Cooling the solution down to room temperature resulted in the formation of CD active aggregates whose absorption and CD spectrum differed from the ones recorded directly after the preparation (green lines in Figure S13a). The relative decrease in absorbance of molecularly dissolved species suggested that aggregation was more pronounced after the heating and cooling cycle. Additionally, the shape of the CD exhibited an additional minimum at 677 nm. The zero-crossing point in the CD spectrum of the aggregates prepared by cooling was located at 717 nm. It coincided with the aggregate's wavelength of maximum absorbance. The accordance indicates that the CD spectrum originated from exciton coupling between the neighbouring ***S*-SQ-2** molecules. Since the Cotton effect had a positive couplet at the longer wavelength (around 700 nm), the assemblies have been ascribed to (P)-type helicity. Since the CD spectra were contaminated by linear dichroism (LD) (Figure S13c), it was concluded that the formed structures were rather large.

The recorded cooling curves showed a large hysteresis (Figure S13b) and lower aggregation temperature than typically observed for benzene-1,3,5-tricarboxamides (BTAs) which are governed by hydrogen-bonding driven self-assembly.[7] Since BTAs contain three and not only two amide groups, decreased aggregation temperatures and slow kinetics were not unexpected for double amide functionalised ***S*-SQ-2**. A literature report for a C_{2h} symmetrical squaraine dye mentioned a similar aggregation temperature.[8] In contrast to the reported system, aggregation of ***S*-SQ-2** resulted in formation of J-type and not H-type aggregates which suggested that ***S*-SQ-2** was not stacked in a straight array but in a tilted fashion. Investigation of the N-H and C=O bonds via FT-IR spectroscopy was not comparable with spectra obtained for BTAs but suggested that the carbonyl groups were part of a highly conjugated system. The exact nature

of the aggregates could hence not be ascertained in the presented study. The J-type nature of the aggregates suggest a the formation of tilted stacks driven by intermolecular donor and acceptor interactions.[9]

During performing the experiments with ***S*-SQ-2** and **a-SQ-2**, it was observed that emptied spectroscopical glass cells retained a bluish colour impression. In order to obtain a more quantitative idea of the amount of substance that was remaining in the glass cell, optical properties of the emptied but not cleaned cell were recorded (Figure S13d). In contrast to the results obtained for ***S*-SQ-1**, the measured CD signal was not zero but also retained by roughly one third when the glass cell was simply emptied. The signal must have stemmed from the absorption of a thin chiral layer of ***S*-SQ-2** that had been formed or adsorbed onto the inside of the glass cell. While the temperature dependent CD experiments clearly show that the aggregates are formed upon cooling, it remains unclear whether the aggregates are nucleated in solution and exhibit a high affinity to cover the surface of the glass cell or the latter induces heterogeneous nucleation. Both aggregates were LD active and had a high affinity to the quartz glass surface of the spectroscopic cell. After emptying the glass cell high CD effects were retained by residues of ***S*-SQ-2** that remained sticking to the glass surface (Figure S13c).

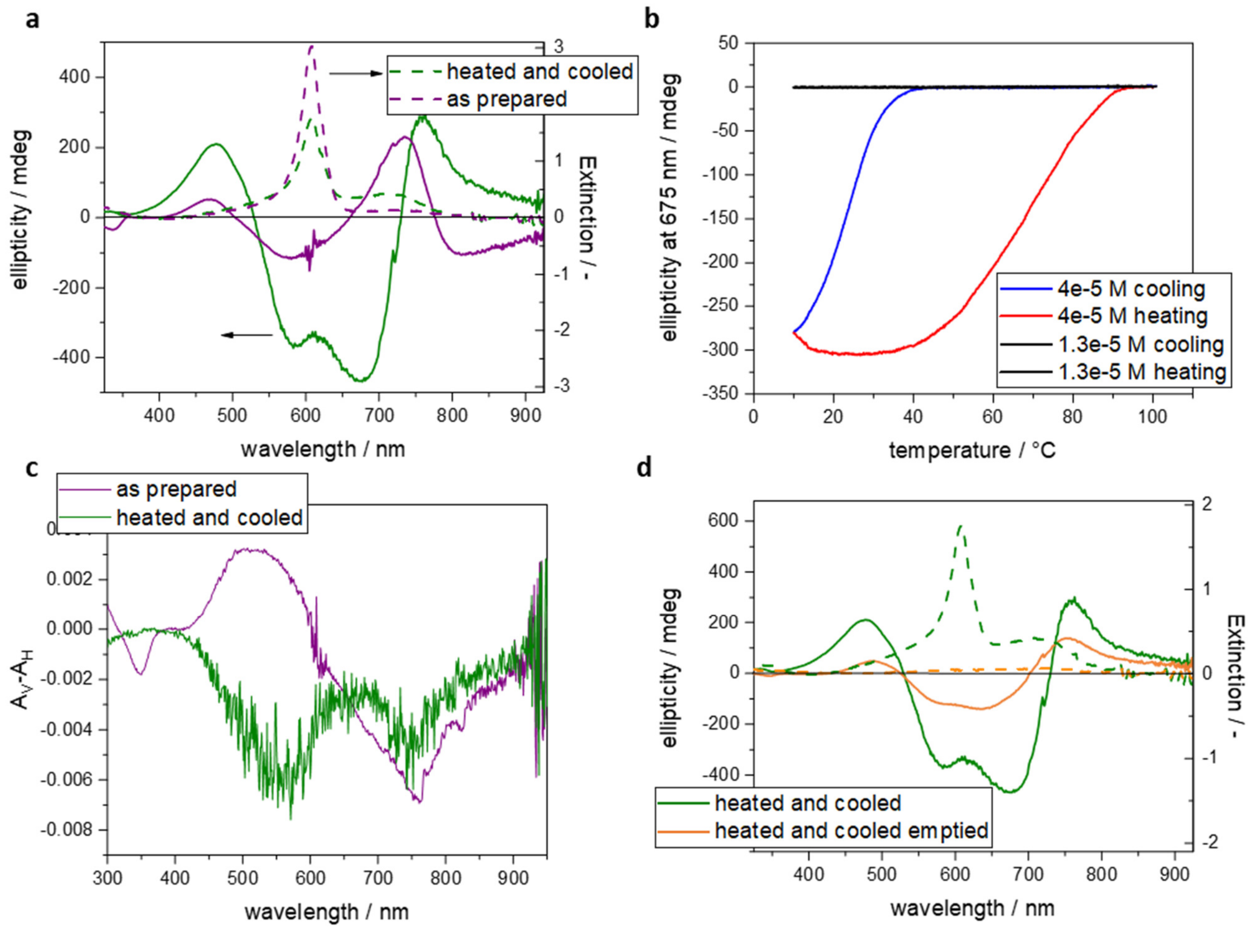

Figure S21: CD-effect measured for a heated and cooled and an as prepared solution of ***S*-SQ-2** (a). Solvent toluene + 5 vol% tetrahydrofuran. B: Variation of the CD effect during cooling and heating cycle, cooling rate: 15 °C/h. The cycle was started with cooling. For the concentration of $1.3 \cdot 10^{-5}$ M no aggregates were formed. D: Emptying a glass cell containing aggregated ***S*-SQ-2** does not removed aggregates from the glass walls.

Recorded spectra

Nuclear magnetic resonance

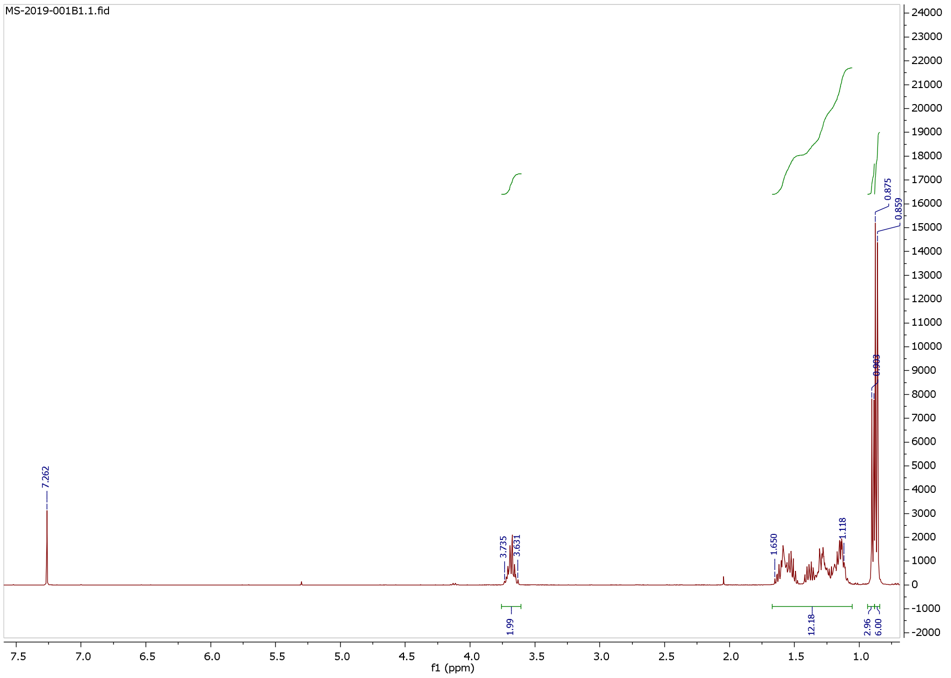

Figure S22: ^{1}H NMR spectrum of [1] (*S*)-3,7-dimethyloctan-1-ol.

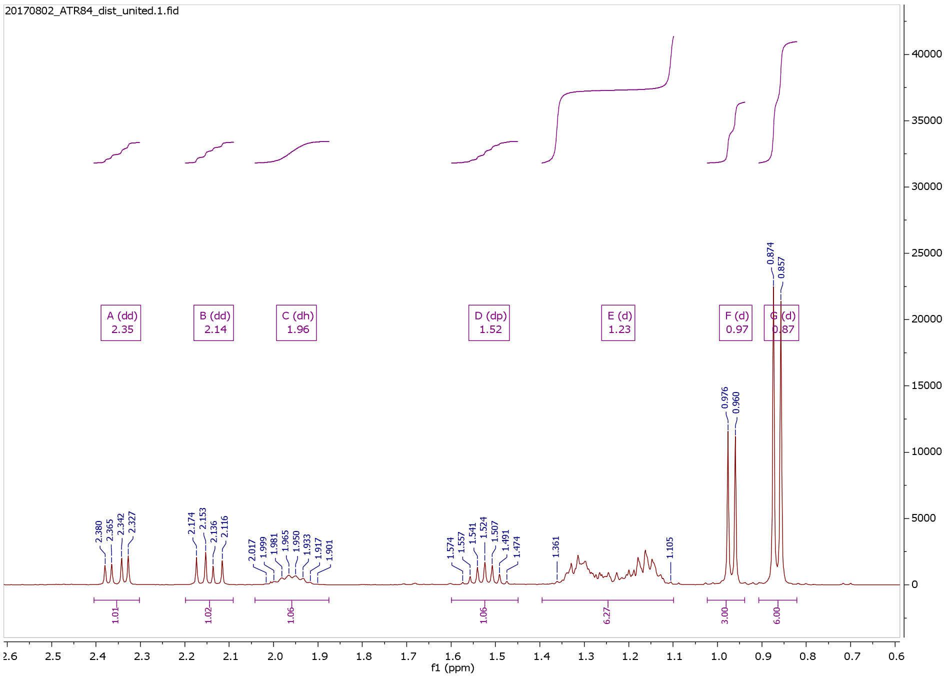

Figure S23: ^{1}H NMR spectrum of [2] (*S*)-3,7-dimethyloctanoic acid.

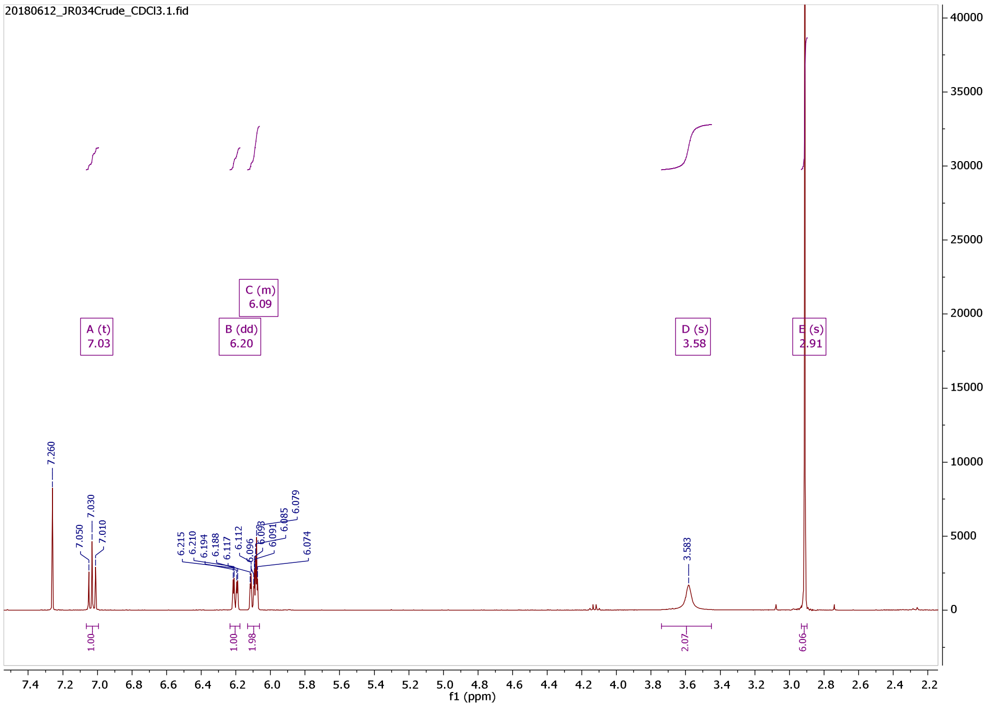

Figure S24: ^{1}H NMR spectrum of [3] N,N-dimethylbenzene-1,3-diamine.

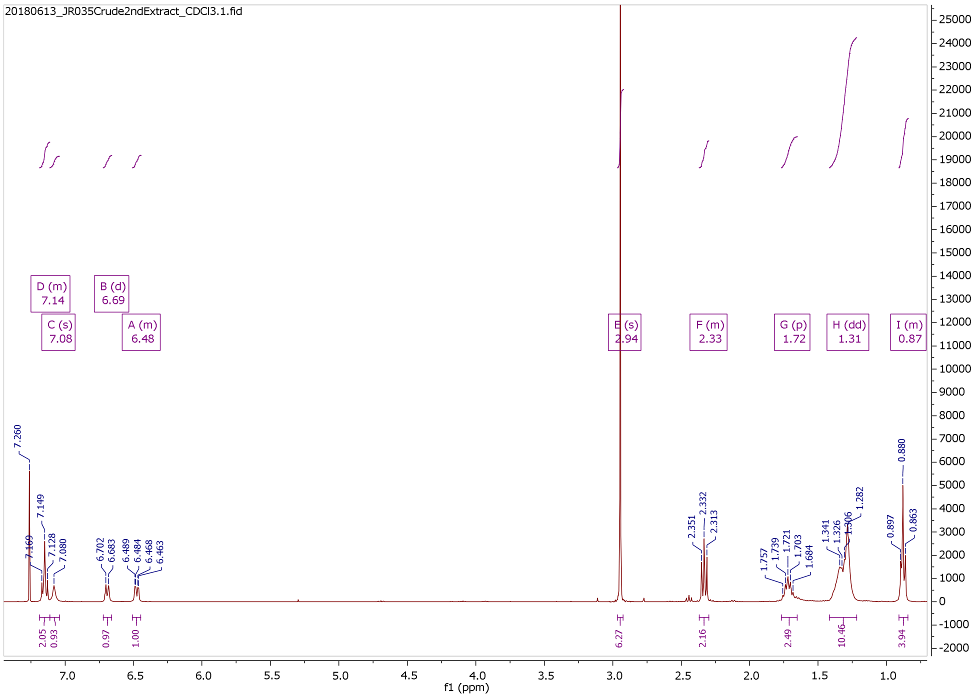

Figure S25: ^{1}H NMR spectrum of [a-4] N-(3-(dimethylamino)phenyl)octanamide.

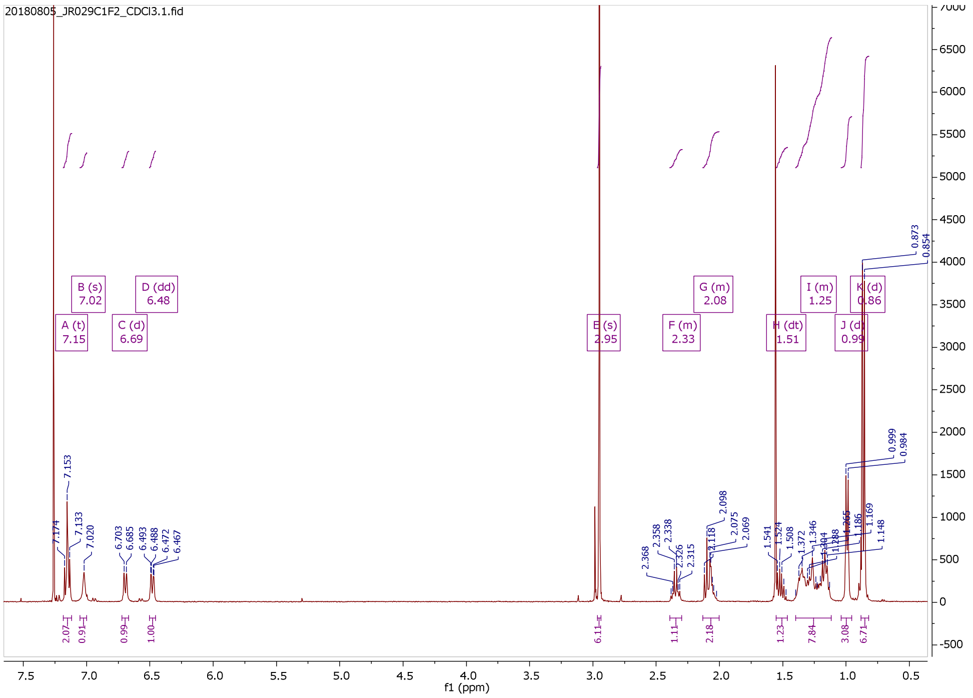

Figure S26: ^{1}H NMR spectrum of [*S*-4] (*S*)-N-(3-(dimethylamino)phenyl)-3,7-dimethyloctanamide.

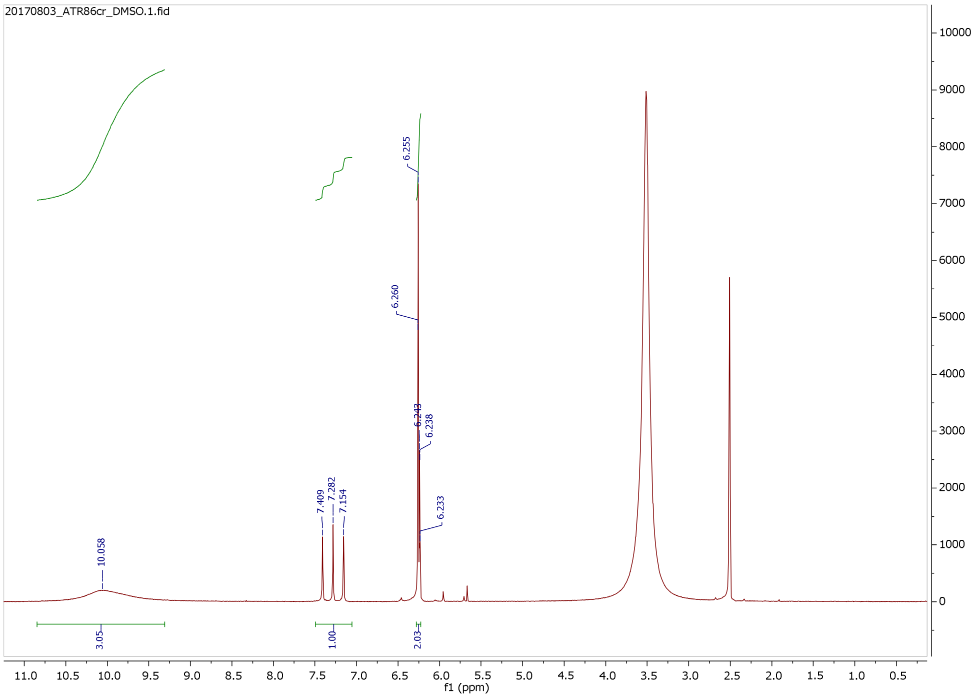

Figure S27: ^{1}H NMR spectrum of [5] 5-aminobenzene-1,3-diol hydrochloride.

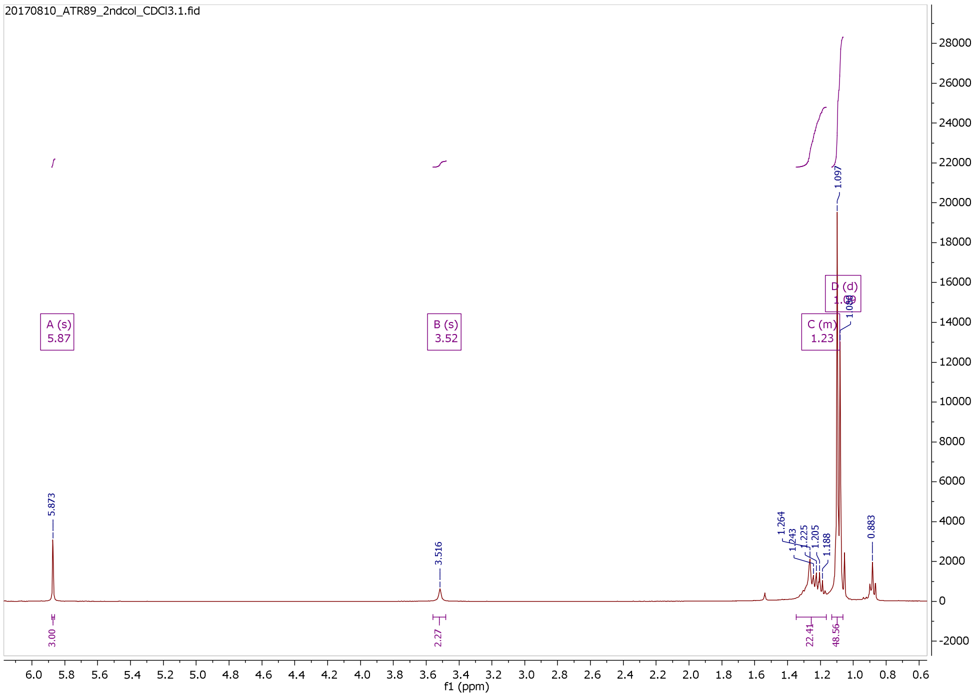

Figure S28: ^{1}H NMR spectrum of [6] 3,5-bis((triisopropylsilyl)oxy)aniline.

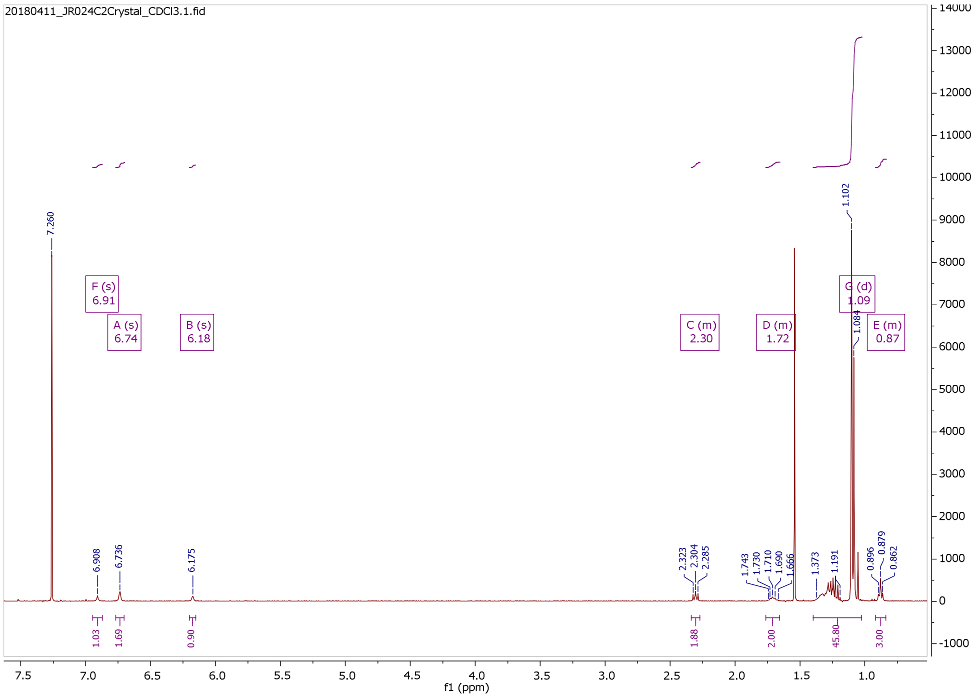

Figure S29: ^{1}H NMR spectrum of [a-7] N-(3,5-bis((triisopropylsilyl)oxy)phenyl)octanamide.

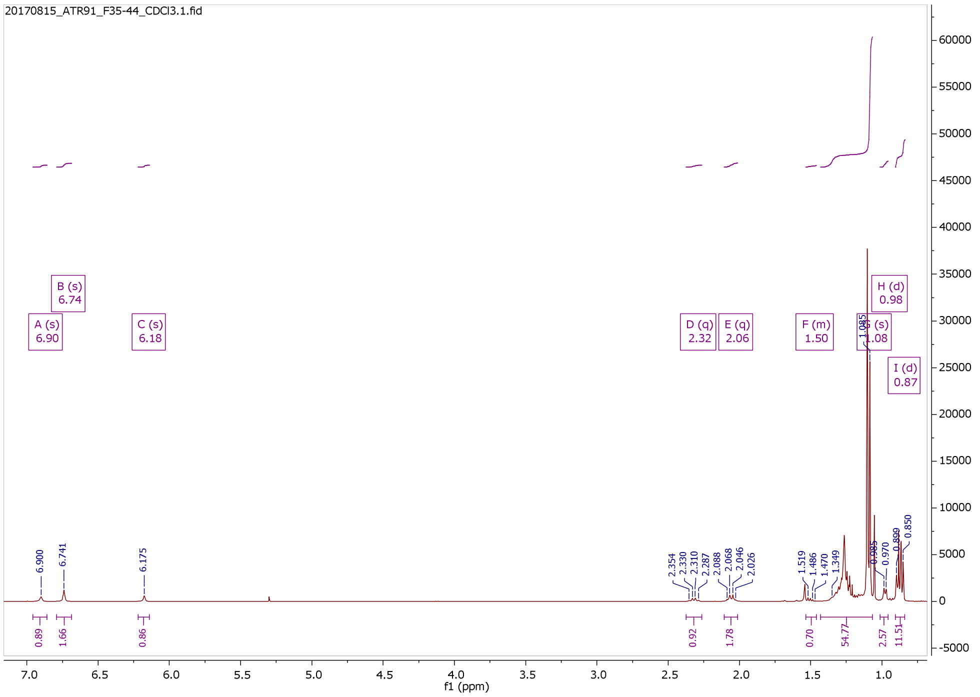

Figure S30: ^{1}H NMR spectrum of [*S*-7] N-(3,5-bis((triisopropylsilyl)oxy)phenyl)octanamide.

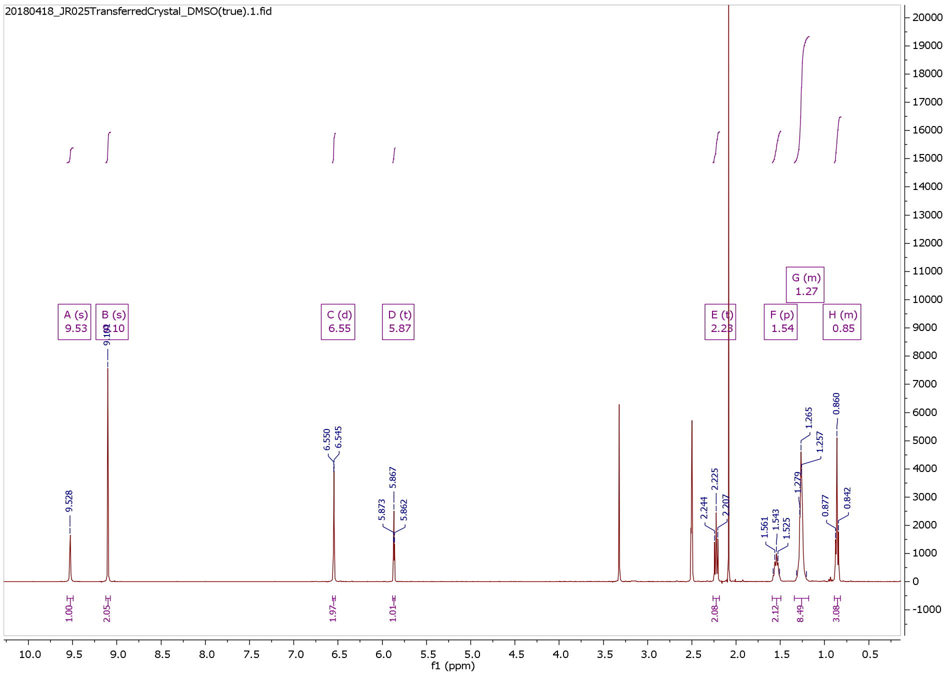

Figure S31: ^{1}H NMR spectrum of [a-8] N-(3,5-dihydroxyphenyl)octanamide.

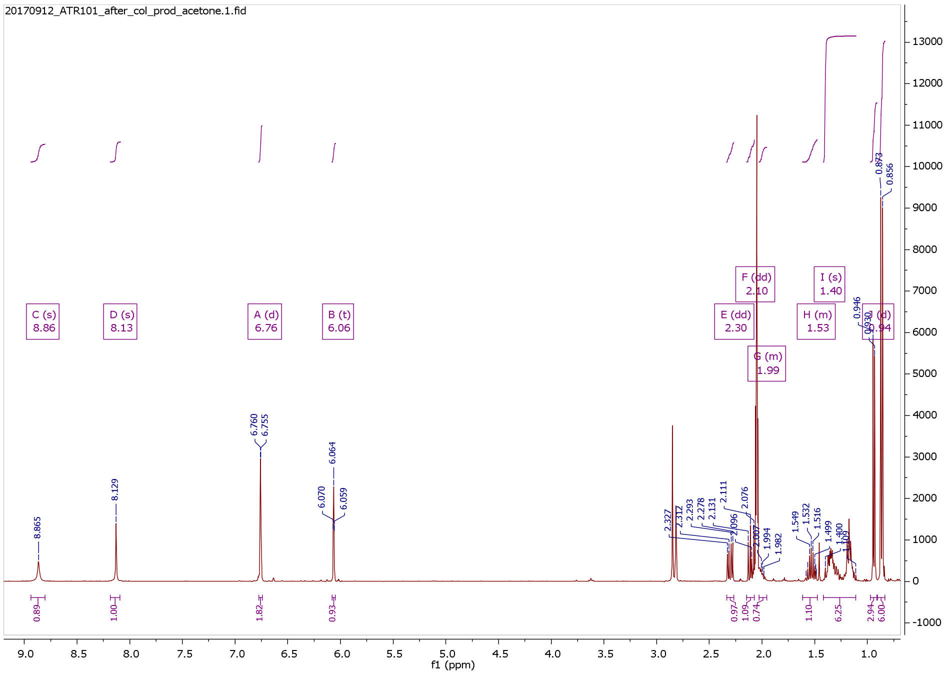

Figure S32: ^{1}H NMR spectrum of [*S*-8] (*S*)-N-(3,5-dihydroxyphenyl)-3,7-dimethyloctanamide.

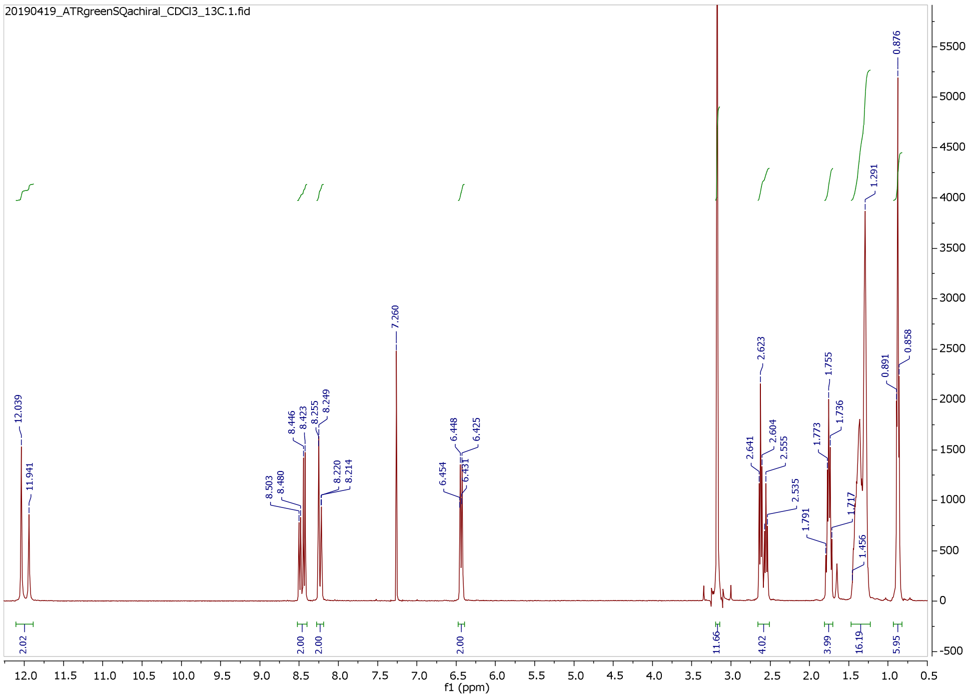

Figure S33: ^{1}H NMR spectrum of **a-SQ-1**.

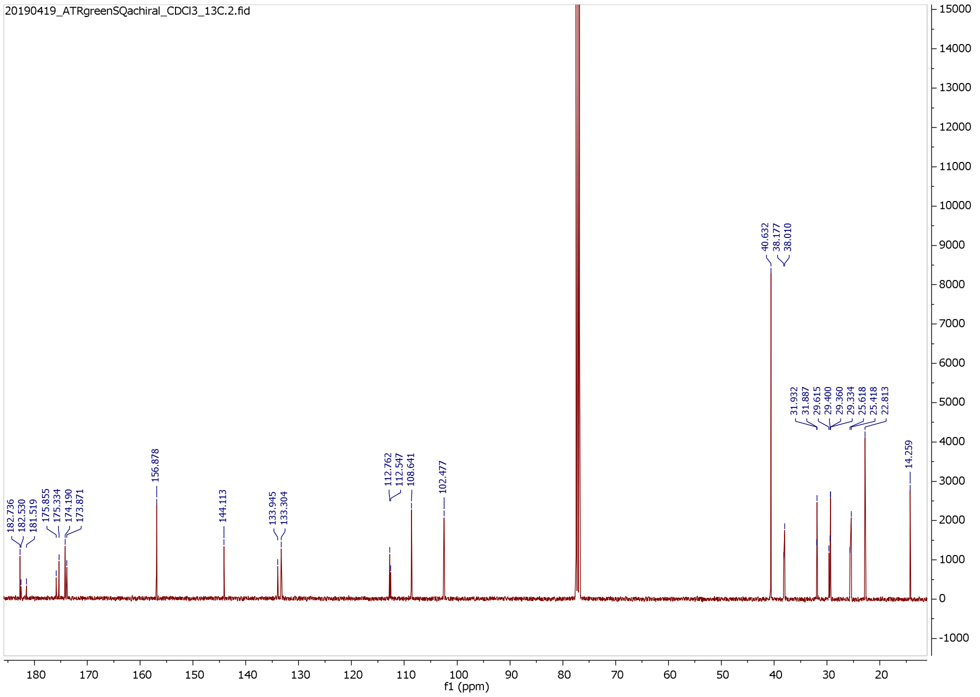

Figure S34: ^{13}C NMR spectrum of **a-SQ-1**.

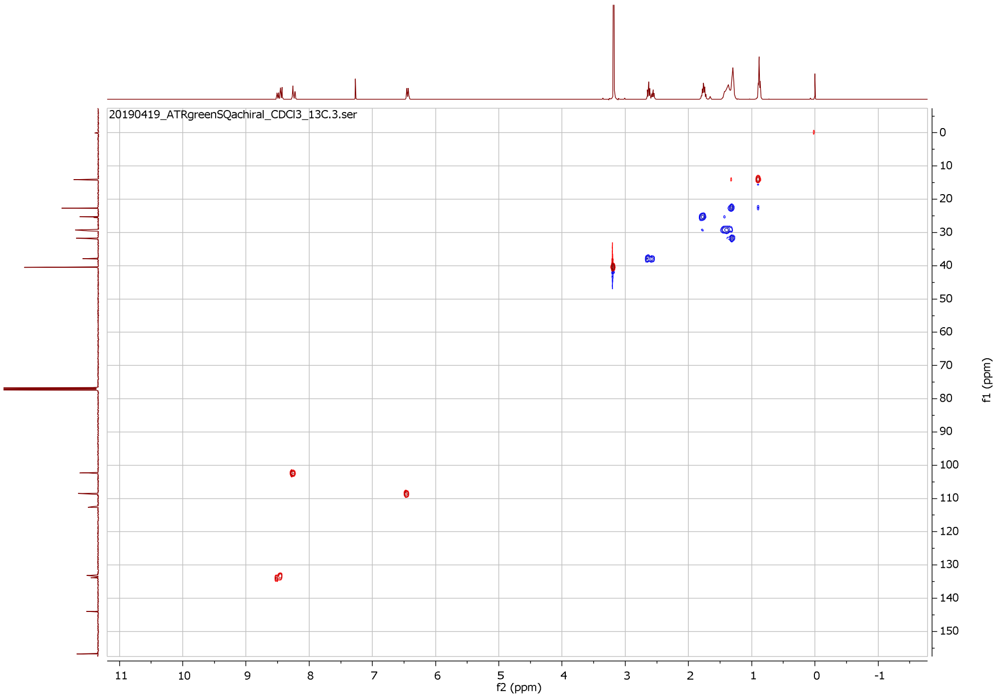

Figure S35: HSQC spectrum of **a-SQ-1**.

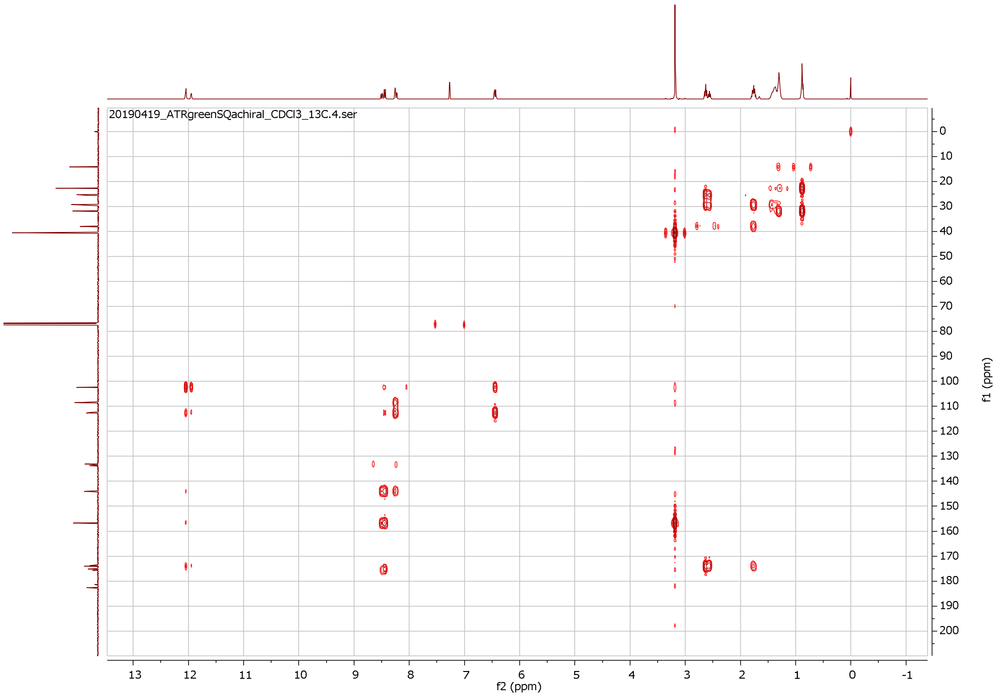

Figure S36: HMBC spectrum of **a-SQ-1**.

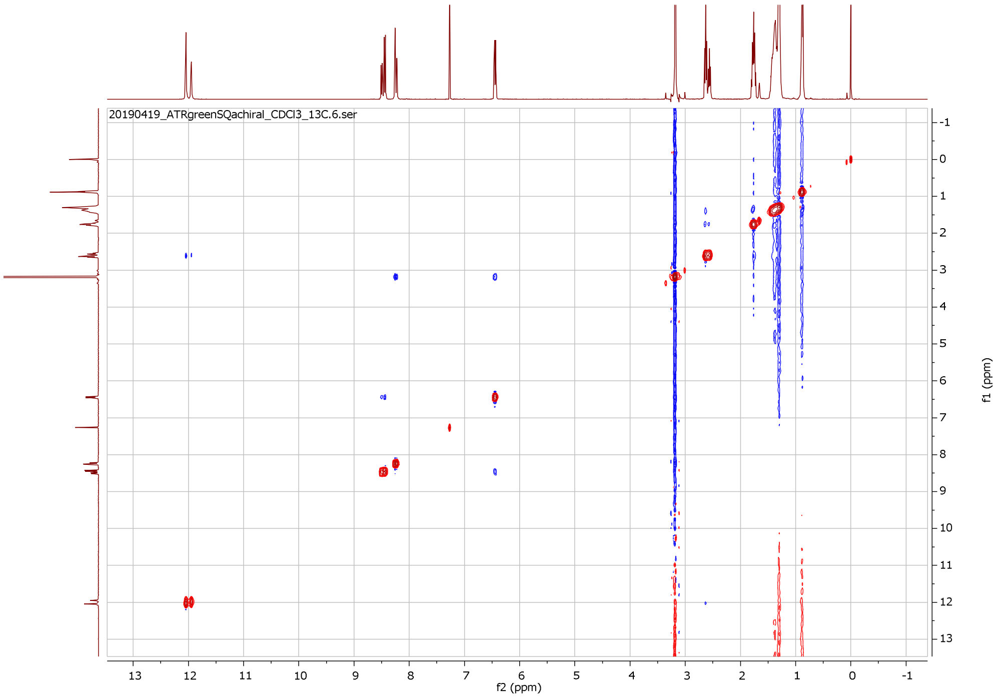

Figure S37: NOESY spectrum of **a-SQ-1**.

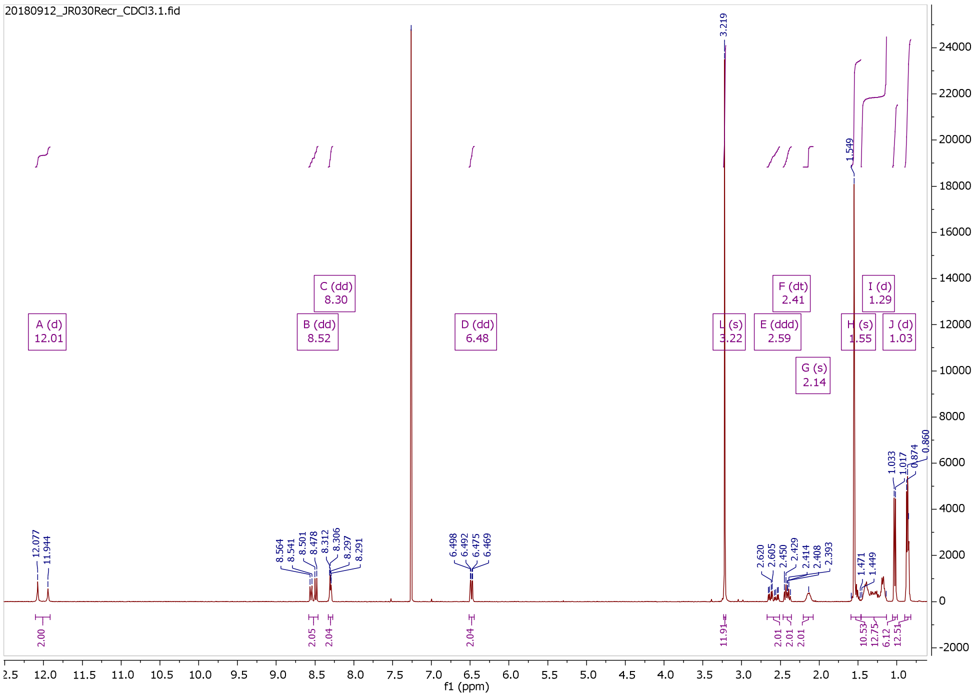

Figure S38: ^{1}H NMR spectrum of ***S*-SQ-1**.

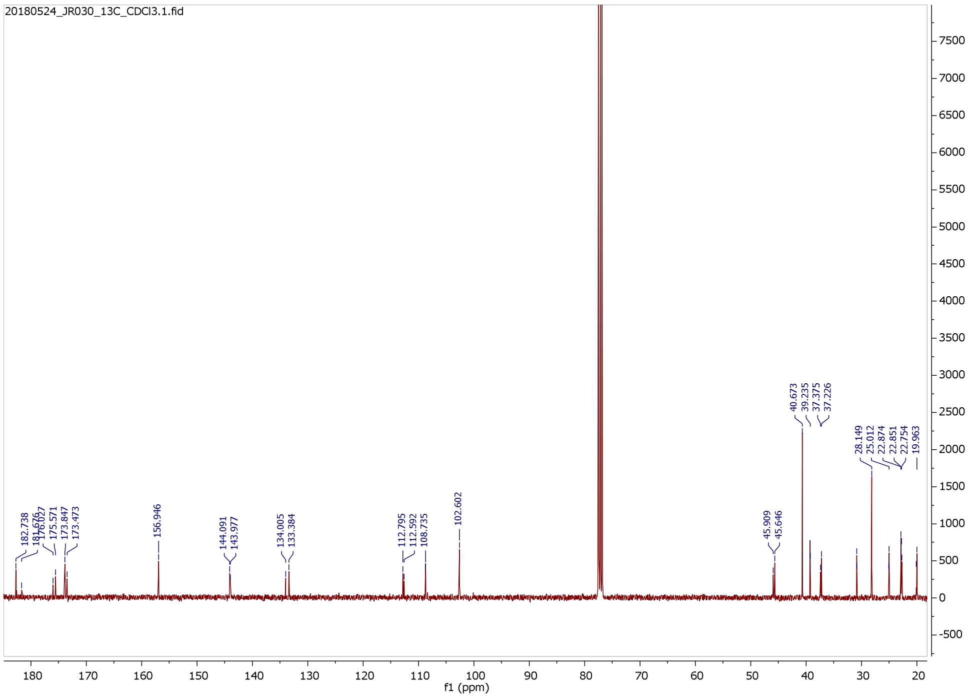

Figure S39: ^{13}C NMR spectrum of ***S*-SQ-1**.

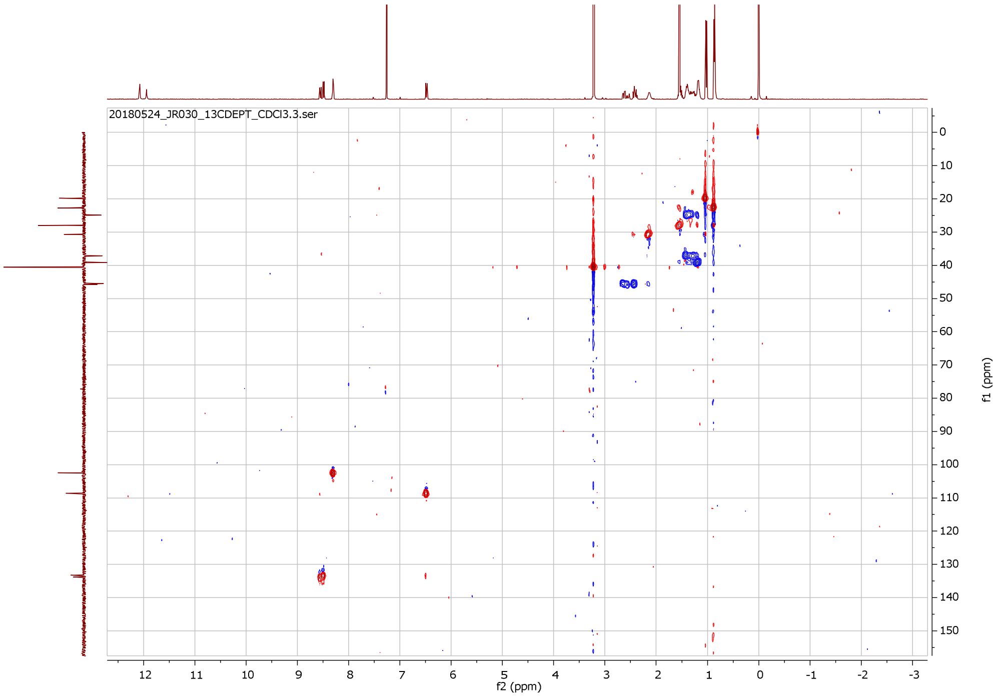

Figure S40: HSQC spectrum of ***S*-SQ-1**.

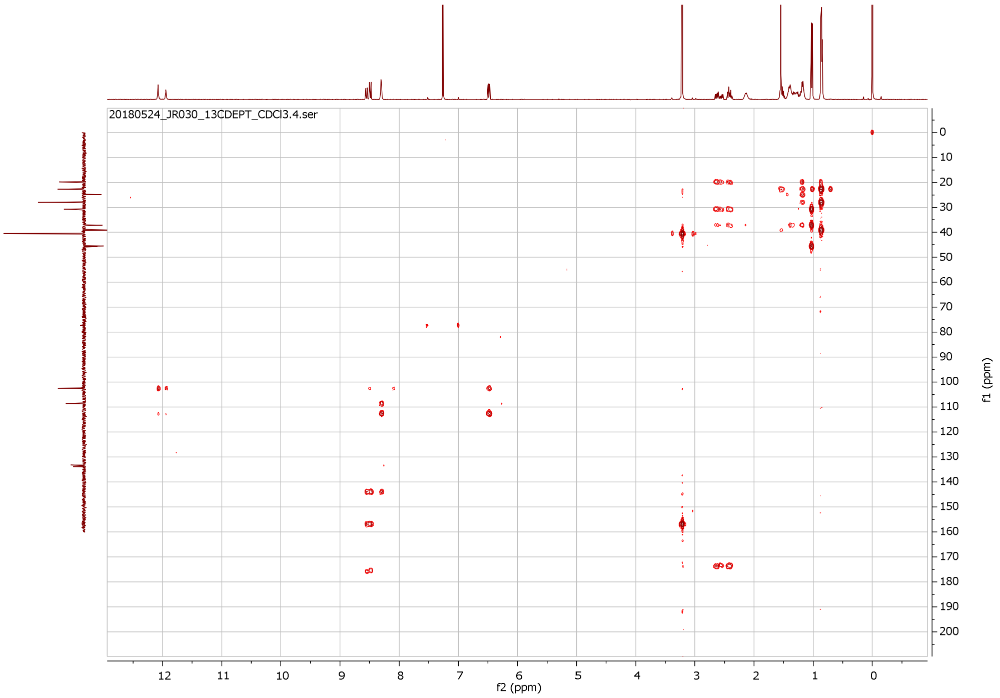

Figure S41: HMBC spectrum of ***S*-SQ-1**.

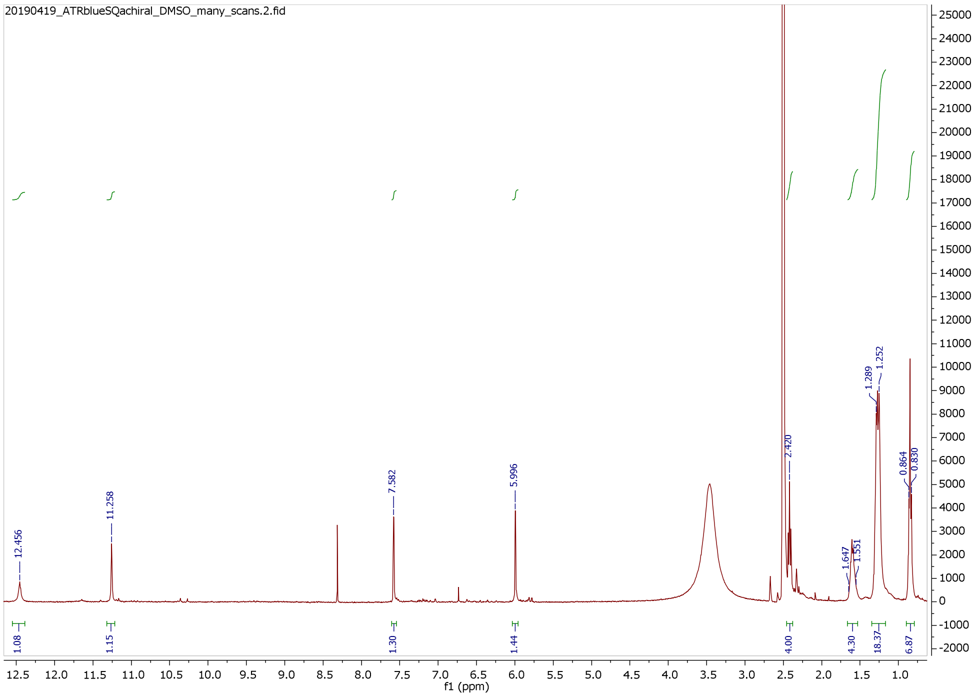

Figure S42: ^{1}H NMR spectrum of **a-SQ-2**.

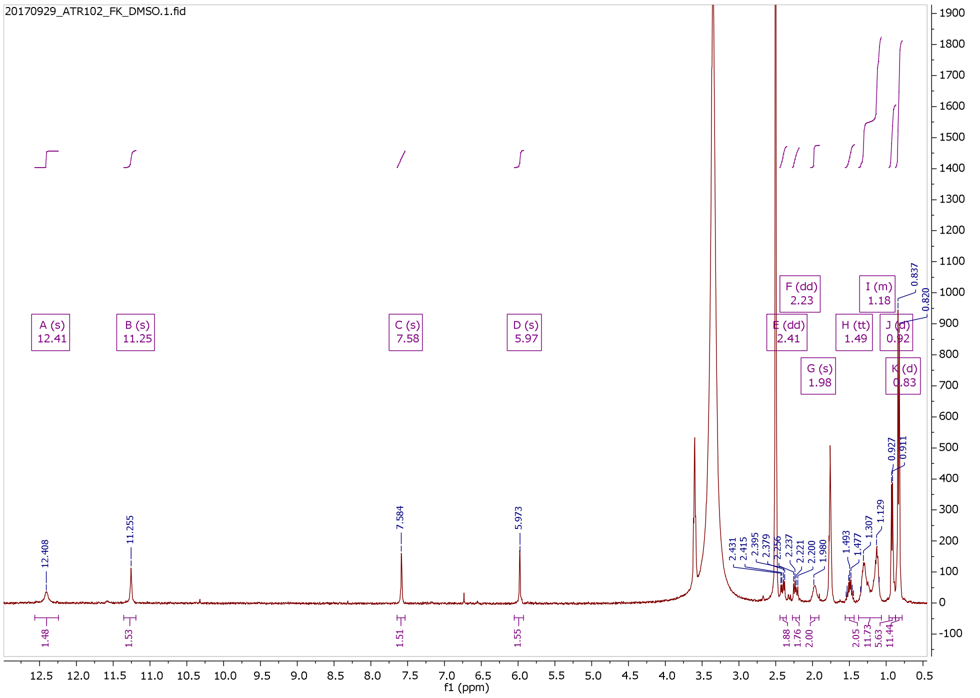

Figure S43: ^{1}H NMR spectrum of ***S*-SQ-2**.

Fourier-transform infrared spectroscopy

For all the dyes, the vibrations of the conjugated system are found around 1600 cm^{-1} ($\nu_{C=C}$, stretching). The vibrations are shifted to relatively small wavenumbers due to the close vicinity of the electron poor squaric unit. Literature reports that the C-O bonds of the squaric core have very low double bond character and the electrons are fully localised in the central squaric unit.[10] The very weak intensity of the C=O stretching at wavenumbers around 1700 cm^{-1} is probably caused by the C-O bonds of the amides which are conjugated to the electron deficient aromatic system. The amides N-H bending ν_{N-H} is noticed around 1580 cm^{-1}.

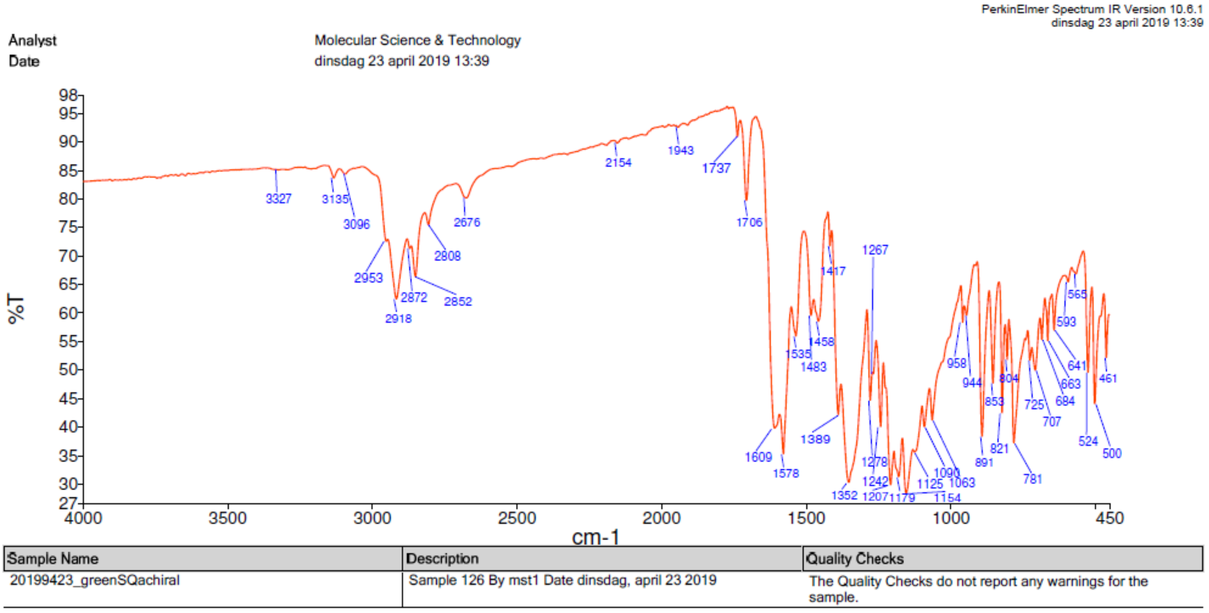

Figure S44: FT-IR spectrum of **a-SQ-1**.

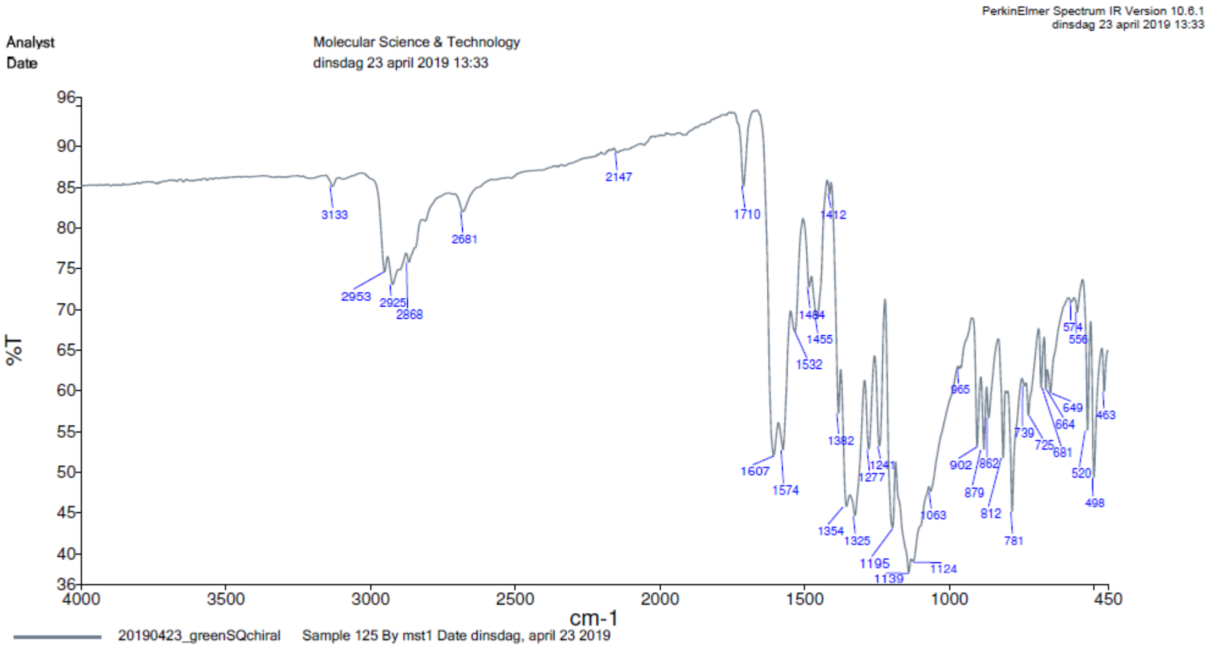

Figure S45: FT-IR spectrum of ***S*-SQ-1**.

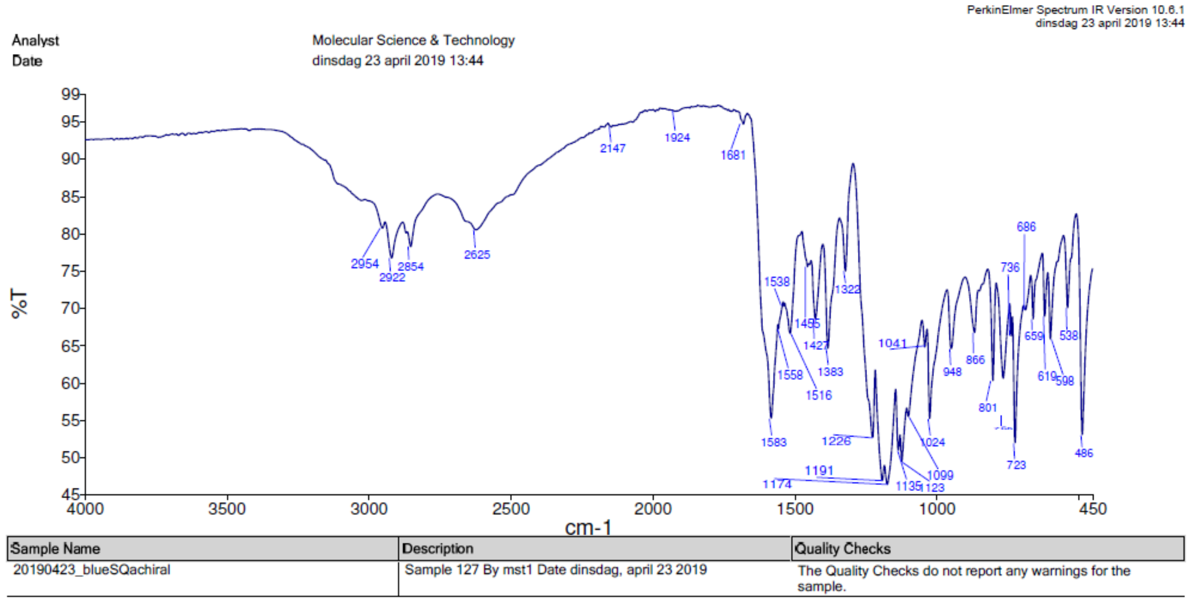

Figure S46: FT-IR spectrum of **a-SQ-2**.

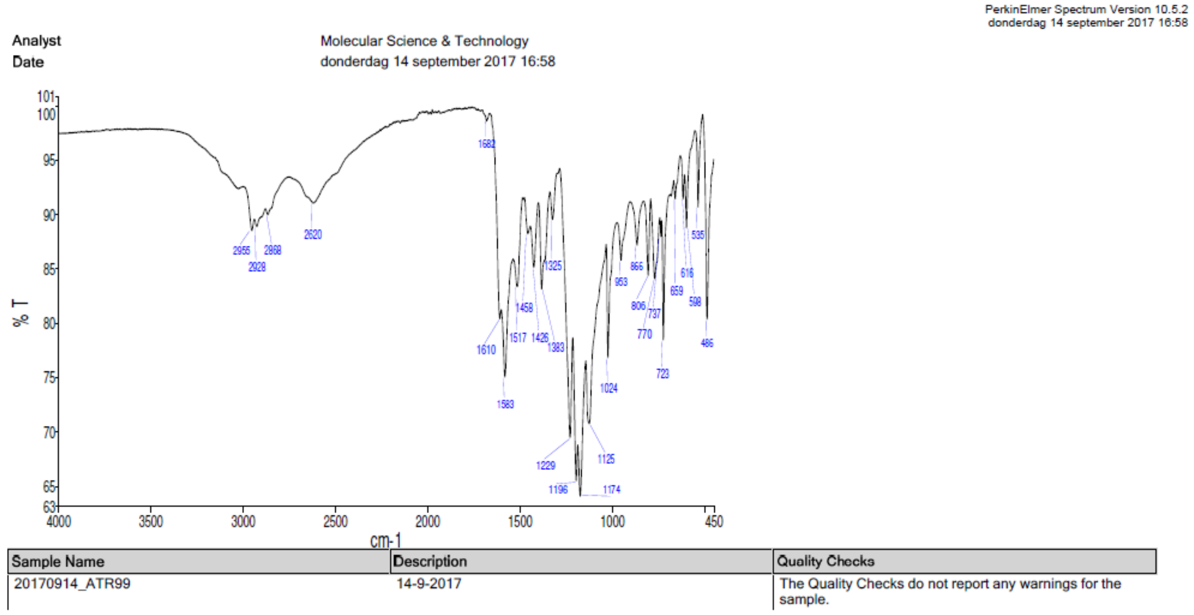

Figure S47: FT-IR spectrum of ***S*-SQ-2**.

Matrix assisted laser desorption/ionisation time-of-flight mass spectrometry

D:\Data\Users 2019\Ralf_Bovee\April\Andreas_Rosch\April242019\AS_greenSQachiral_CHCA_C3_dg10_ls30_PW\0_C3\1\1Ref

Comment 1 CHCA/DCTB in THF calibration in D:\Data\Users 2019\Ralf_Bovee\April\Andreas_Rosch\April242019

Comment 2

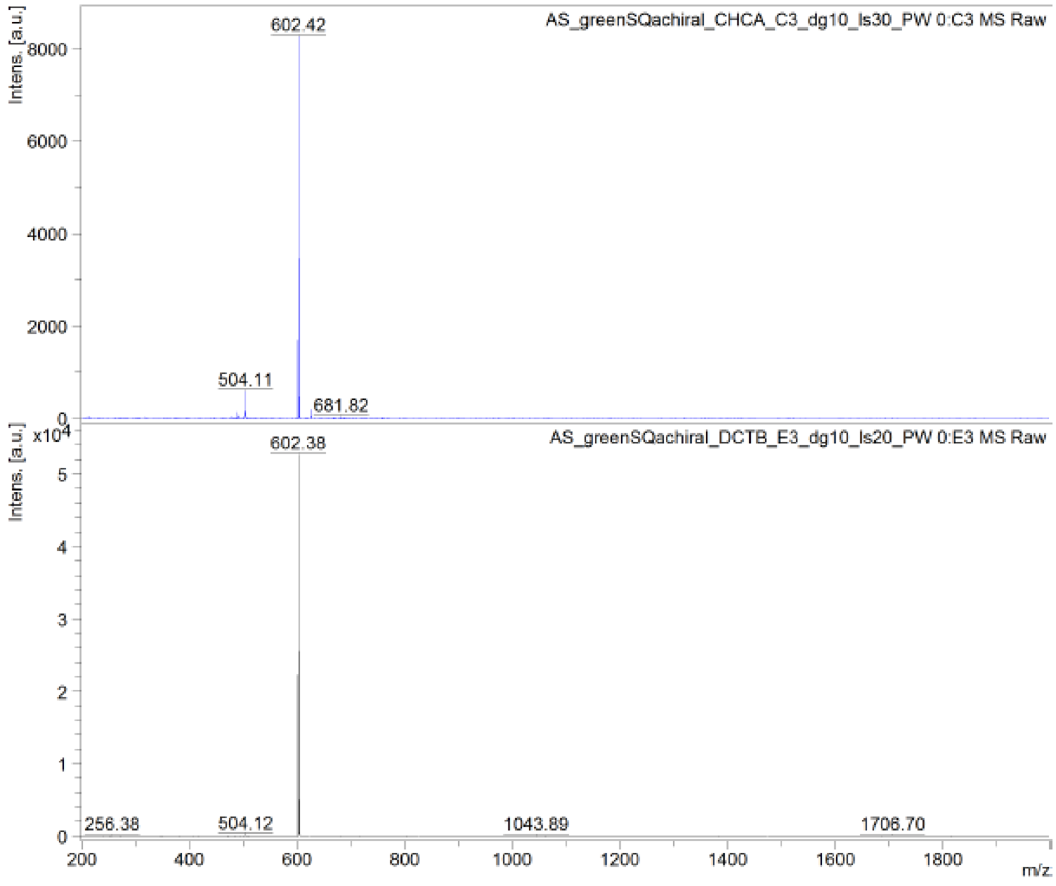

Acquisition Parameter

Date of acquisition	2019-04-24T11:50:21.551+02:00
Acquisition method name	D:\Lou\MALDI_Method\RP_700-3500_Da.par
Aquisition operation mode	Reflector
Voltage polarity	POS
Number of shots	500
Name of spectrum used for calibration	
Calibration reference list used	Cesium tri jodide positive mode

Instrument Info

User	smo379
Instrument	ST-A2130
Instrument type	autoflex

Bruker Daltonics flexAnalysis printed: 24-Apr-19 13:16:34

Figure S48: MALDI-TOF spectrum of a-**SQ-1**.

D:\Data\Users 2018\Ralf_Bovee\May\Jorn_Robben\May232018\JR030_CHCA_C7_ls10_RW\0_C7\1\1Ref

Comment 1 CHCA/DCTB in THF calibration in D:\Data\Users 2018\Ralf_Bovee\May_SFD\Shaji_Varghese\May232018

Comment 2

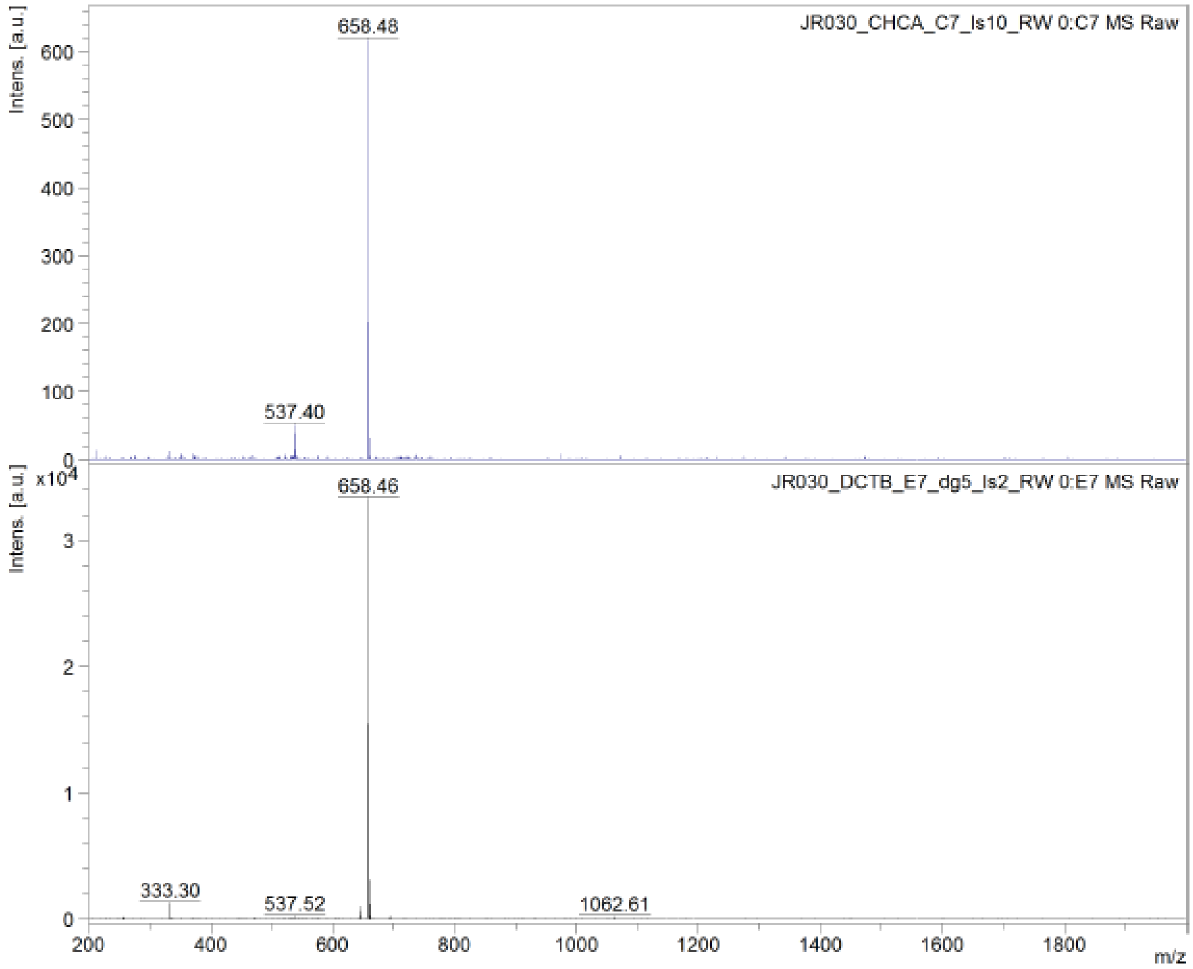

Acquisition Parameter

Date of acquisition	2018-05-23T15:13:57.544+02:00
Acquisition method name	D:\Method_after_Dec0117_Lou\RP_700-3500_Da.par
Aquisition operation mode	Reflector
Voltage polarity	POS
Number of shots	500
Name of spectrum used for calibration	
Calibration reference list used	Cesium tri jodide positive mode

Instrument Info

Bruker Daltonics flexAnalysis printed: 23-May-18 16:51:42

Figure S49: MALDI-TOF spectrum of ***S*-SQ-1**.

D:\Data\Users 2018\Ralf_Bovee\May\Jorn_Robben\May032018\JR027_DCTB_neg_E3_dg5_ls2_RW\0_E3\1\1Ref

Comment 1 CHCA/DCTB in THF calibration in D:\Data\Users 2018\Ralf_Bovee\May\Jorn_Robben\May032018

Comment 2

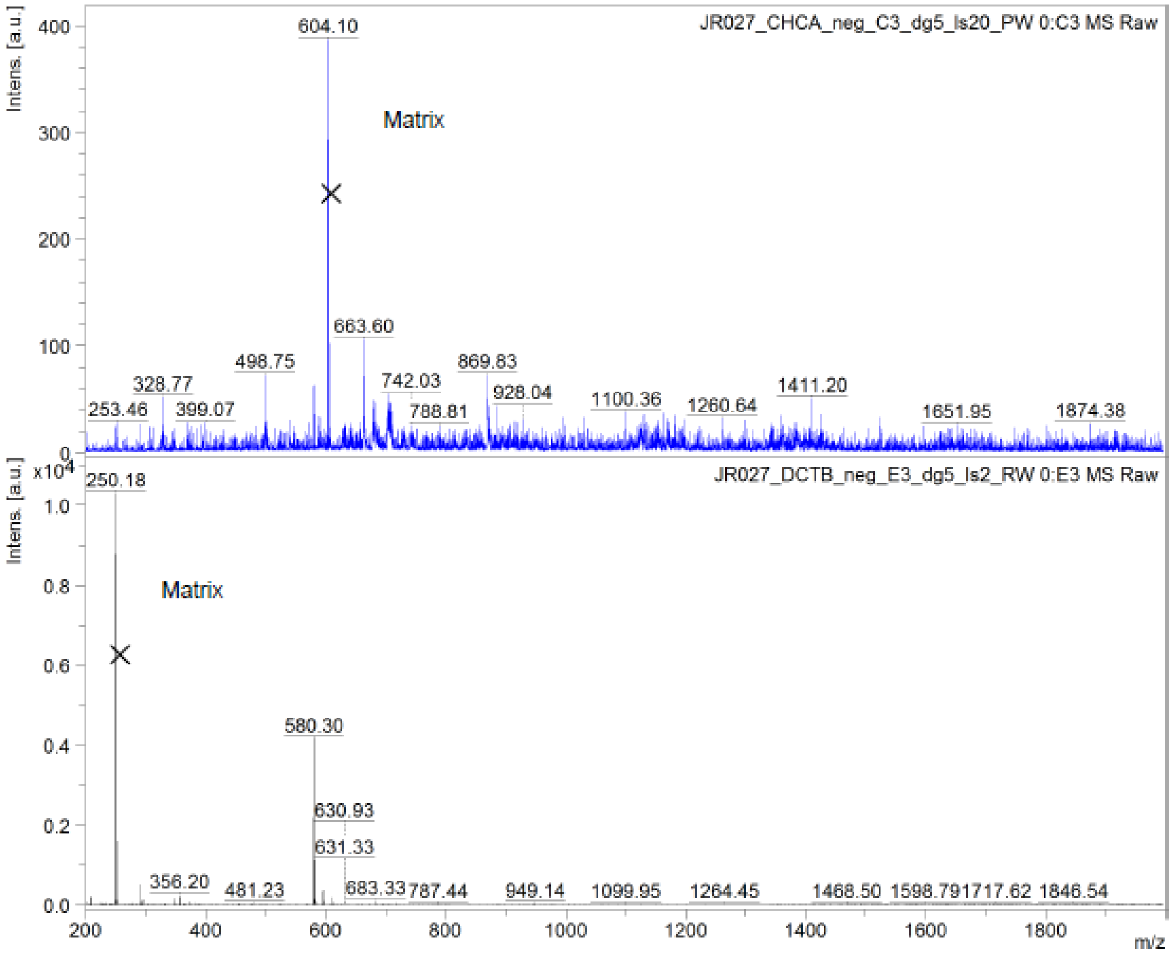

Acquisition Parameter

Date of acquisition	2018-05-03T13:19:38.695+02:00
Acquisition method name	D:\Method_after_Dec0117_Lou\RN_900-4500_Da.par
Aquisition operation mode	Reflector
Voltage polarity	NEG
Number of shots	500
Name of spectrum used for calibration	
Calibration reference list used	Cesium tri jodide negative mode

Instrument Info

Bruker Daltonics flexAnalysis printed: 03-May-18 14:35:00

Figure S50: MALDI-TOF spectrum of **a-SQ-2**.

D:\Data\Users
~~2018\Ralf_Bovee\November\Andreas_Rosch\Nov022018\ATR_SQblue_chiral_DCTB_E4_dg10_ls10_RW\0_E4\1\1Ref~~

Comment 1	CHCA/DCTB in THF calibration in D:\Data\Users
Comment 2	2018\Ralf_Bovee\November\Mathijs_Mabesoone\Nov022018

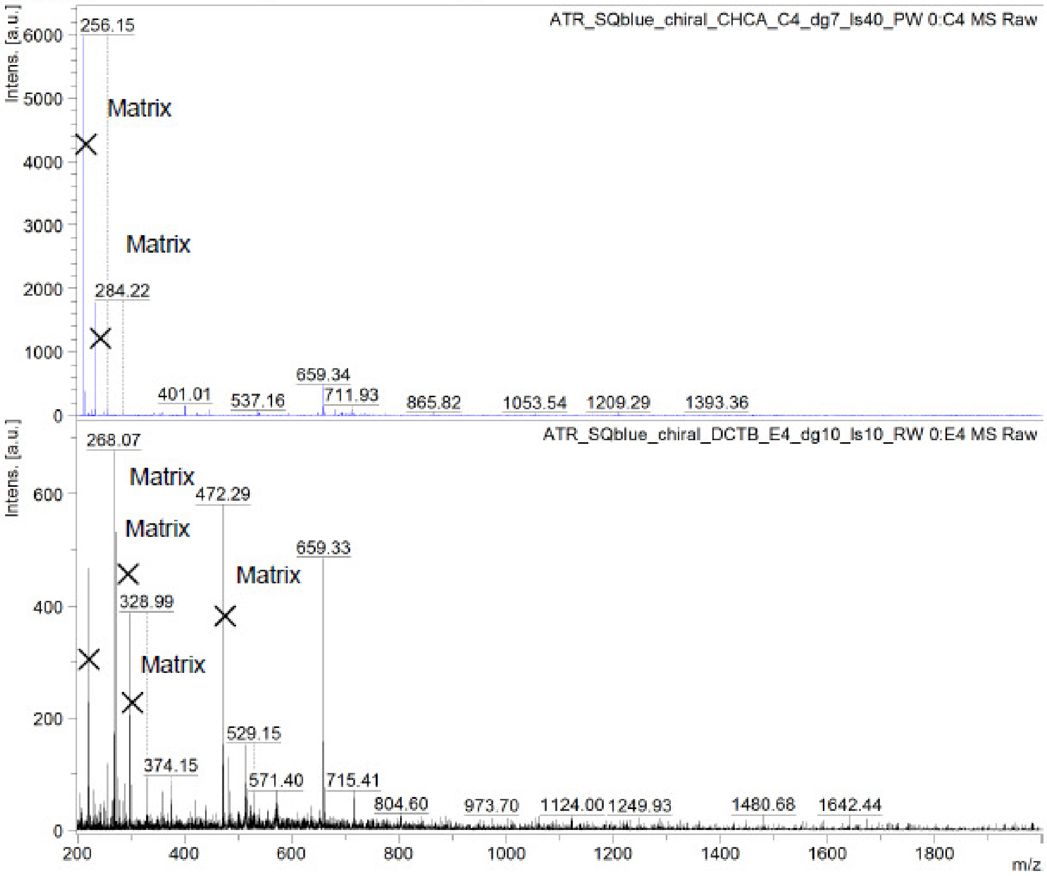

Acquisition Parameter

Date of acquisition	2018-11-02T12:10:27.831+01:00
Acquisition method name	D:\Lou\MALDI_Method\RP_700-3500_Da.par
Aquisition operation mode	Reflector
Voltage polarity	POS
Number of shots	500
Name of spectrum used for calibration	
Calibration reference list used	Cesium tri jodide positive mode

Instrument Info

User	smo379
Instrument	ST-A2130
Instrument type	autoflex

Bruker Daltonics flexAnalysis printed: 02-Nov-18 14:42:48

Figure S51: MALDI-TOF spectrum of ***S*-SQ-2**.

Literature

[1] C. Lambert, F. Koch, S. F. Völker, A. Schmiedel, M. Holzapfel, A. Humeniuk, M. I. S. Röhr, R. Mitric, T. Brixner, *J. Am. Chem. Soc.* **2015**, *137*, 7851.

[2] K. Hunag, A. Rhys, *Proc. R. Soc. Lond. A* **2000**, *204*, 74.

[3] K. Y. Law, *Chem. Rev.* **1993**, *93*, 449.

[4] E. Buncel, A. J. McKerrow, P. M. Kazmaier, *J. Chem. Soc. Chem. Commun.* **1992**, 1242.

[5] J. Griffiths, J. Mama, J. Gri, J. Mama, *Dye. Pigment.* **2000**, *44*, 9.

[6] C. Reichardt, *Chem. Rev.* **1994**, *94*, 2319.

[7] B. Göhler, V. Hamelbeck, T. Z. Markus, M. Kettner, G. F. Hanne, Z. Vager, R. Naaman, H. Zacharias, *Science* **2011**, *331*, 894.

[8] M. Kettner, B. Göhler, H. Zacharias, D. Mishra, V. Kiran, R. Naaman, C. Fontanesi, D. H. Waldeck, S. Sek, J. Pawowski, J. Juhaniewicz, *J. Phys. Chem. C* **2015**, *119*, 14542.

[9] K. M. Alam, S. Pramanik, *Adv. Funct. Mater.* **2015**, *25*, 3210.

[10] S. P. Mathew, P. C. Mondal, H. Moshe, Y. Mastai, R. Naaman, *Appl. Phys. Lett.* **2014**, *105*, 1.

[11] J. M. Abendroth, K. M. Cheung, D. M. Stemer, M. S. El Hadri, C. Zhao, E. E. Fullerton, P. S. Weiss, *J. Am. Chem. Soc.* **2019**, *141*, 3863.

[12] B. P. Bloom, V. Kiran, V. Varade, R. Naaman, D. H. Waldeck, *Nano Lett.* **2016**, *16*, 4583.

[13] M. Suda, Y. Thathong, V. Promarak, H. Kojima, M. Nakamura, T. Shiraogawa, M. Ehara, H. M. Yamamoto, *Nat. Commun.* **2019**, *10*, 1.

[14] M. Á. Niño, I. A. Kowalik, F. J. Luque, D. Arvanitis, R. Miranda, J. J. De Miguel, *Adv. Mater.* **2014**, *26*, 7474.

[15] P. J. M. M. Stals, J. C. Everts, R. De Bruijn, I. A. W. W. Filot, M. M. J. J. Smulders, R. Martín-Rapún, E. A. Pidko, T. F. A. A. De Greef, A. R. A. A. Palmans, E. W. Meijer, *Chem. Eur. J.* **2010**, *16*, 810.

[16] U. Mayerhöffer, F. Würthner, *Chem. Sci.* **2012**, *3*, 1215.

[17] S. Das, K. J. G. Thomas, K. J. G. Thomas, P. V. Kamat, M. V. George, *J. Phys. Chem.* **1994**, *98*, 9291.
